# Second‐Sphere Aromatic Substitutions That Tune H‐Atom Transfer Reactivity Observed for a Series of *S* = 1 Nonheme Fe^IV^=O Complexes: An Enthalpy‐Entropy Compensation Effect

**DOI:** 10.1002/anie.1885847

**Published:** 2026-09-23

**Authors:** Abhishek Das, Jin Xiong, Victor G. Young, Yisong Guo, Marcel Swart, Lawrence Que

**Affiliations:** ^1^ Department of Chemistry University of Minnesota Minneapolis Minnesota USA; ^2^ Department of Chemistry Carnegie Mellon University Pittsburgh Pennsylvania USA; ^3^ IQCC and Department of Chemistry University of Girona Girona Spain; ^4^ ICREA Barcelona Spain

**Keywords:** electronically tuned aromatic substituted NTB ligand backbone, enthalpy‐entropy compensation effect, High‐valent Fe(IV)=O, hydrogen atom transfer (HAT) reactivity, second sphere effect

## Abstract

Synthetic nonheme Fe^IV^═O complexes reported thus far often have *S* = 1 ground states, demonstrating markedly lower oxidative reactivity in C─H bond breakage compared to their *S* = 2 counterparts. Recently, we have identified that incorporating cycloalkyl groups into the second coordination sphere of the nitrilotris(benzimidazole) ligand produce the most reactive Fe^IV^═O complexes within the tripodal ligand family, exhibiting an *S* = 1 ground state. Here, we have synthesized six *S* = 1 complexes having the NTB ligand framework with either electron‐rich or electron‐poor aromatic groups in the secondary coordination sphere of the ligand. These complexes have been characterized by Mössbauer data at 4 K and ^1^H NMR spectra at 193 K with quite similar electronic characteristics despite having distinct electronically tuned ligands. Nevertheless, the complexes show substrate oxidation rates that differ by nearly an order of magnitude from each other, giving rise to a notable linear correlation between activation enthalpies and activation entropies of HAT reactions. These outcomes thus enable further investigation of the Enthalpy‐Entropy Compensation Effect (EECE) in HAT reactions involving these oxoiron(IV) complexes. Finally, the role played by steric attributes of the secondary coordination sphere group in regulating the reactivity of this series of complexes has been explored.

## Graphical abstract

A series of oxoiron(IV) complexes was synthesized to determine the impact of electronically tuned aromatic substitution in the secondary sphere of the ligand. Despite possessing identical electronic properties, they show significant differences in C─H oxidation reactions. A linear correlation between ΔH^‡^ and ΔS^‡^ was found, indicating enthalpy‐entropy compensation in Hydrogen Atom Transfer reactivity, which is influenced by steric features.

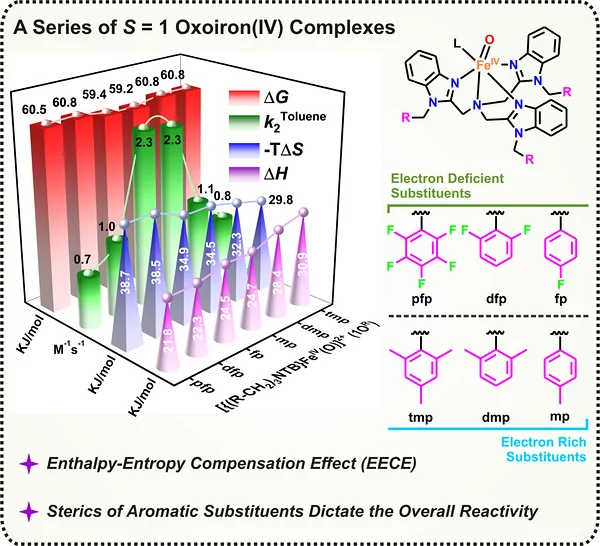

## 1 Introduction

High‐valent nonheme oxoiron(IV) species are used as intermediates in the catalytic processes [1, 2, 3, 4, 5] of many enzymes [6, 7, 8, 9, 10, 11, 12, 13]. The mechanism proposed for this class of enzymes entails the activation of O_2_ within the nonheme iron(II) active site with a bound organic cofactor to generate an oxoiron(IV) species that serves as the active oxidant. This key species has been trapped and characterized for many enzymes [6, 7, 14, 15] and found to carry out an extensive array of difficult metabolic transformations, such as hydroxylation, desaturation of unactivated alkane C─H bonds, and epoxidation or cis‐dihydroxylation of olefins [6, 14, 16, 17, 18, 19, 20, 21, 22, 23, 24, 25, 26]. Most of the nonheme iron enzymes in nature employ *S* = 2 Fe(IV)═O oxidants [6, 14, 27], but Guo and coworkers have recently discovered the first evidence for a nonheme *S* = 1 oxoiron(IV) enzymatic species in biology [13].

The potent oxidative properties associated with these enzymatic Fe^IV^═O intermediates have motivated efforts to develop structural and functional synthetic analogs of the natural enzymes, supported by different polydentate ligands [27, 28, 29, 30]. Contrary to enzymes, most synthetic nonheme oxoiron(IV) complexes have *S* = 1 ground states [29, 30, 31], with just a handful of synthetic *S* = 2 oxoiron(IV) species reported to date [32, 33, 34, 35, 36, 37, 38, 39]. Compared to their *S* = 2 counterparts, the synthetic *S* = 1 complexes tend to display slower reactivities [30, 31]. In their seminal work, Shaik and coworkers provided a theoretical explanation for the observed reactivities of these *S* = 1 model complexes based on a two‐state reactivity (TSR) model [40, 41, 42]. Because of exchange‐enhanced reactivity, the *S* = 1 oxoiron(IV) complex is able to switch to a comparatively low‐lying *S* = 2 excited state during the reaction, which lowers the reaction barrier [41, 43, 44, 45, 46, 47, 48]. Theoretical calculations predicted all *S* = 1 Fe^IV^═O model complexes to exhibit such spin‐switching behavior, with the extent of switching dictating the reactivity of the model complexes [41, 42], thereby giving rise to the evolution of a more complex picture [43]. This idea has recently gained significant relevance by a newly found experimental correlation between hydrogen atom abstraction rates of *S* = 1 Fe^IV^═O model complexes and the energies of their *S* = 2 excited states [49]. Solomon and coworkers have found direct evidence for the mixing of *S* = 2 states into the *S* = 1 ground state via spin−orbit coupling that has been attributed to the relatively weak equatorial field of the [Fe^IV^O(TMC)(CH_3_CN)]^2+^ (TMC = 1,4,8,11‐tetramethyl‐1,4,8,11‐tetraazacyclotetradecane) [50] complex. Interestingly, a limited number of synthetic systems showed HAT rates approaching those seen for taurine oxidation by the nonheme oxygenase TauD‐*J*, which include the *S* = 2 [(TQA)Fe^IV^(O)(MeCN)]^2+^ (TQA = tris(2‐quionolylmethyl)amine) [37] and [(*^t^* Bu_3_tacn)Fe^IV^(O)]^2+^ (*^t^* Bu_3_tacn = 1,4,7‐tri‐tert‐butyl‐1,4,7‐triazacyclononane) [36] complexes, as well as *S* = 1 [Fe^IV^(O){(R‐CH_2_)_3_NTB}(NCMe)]^2+^ [51, 52, 53] and [(L1‐bispidine)Fe^IV^(O)(Cl)]^+^ (L1‐bispidine = 2,4‐di(pyridine‐2‐yl)‐3,7‐diazabicyclo[3.3.1]nonane‐1‐one) [54, 55] complexes.

The primary‐coordination sphere, that is the axial and equatorial ligands that covalently bind to the metal center, has been the primary focus of synthetic nonheme oxoiron (IV) chemistry thus far [27, 29, 56]. On the other hand, enzymatic systems have developed active sites with a web of weaker non‐covalent connections, called the “second‐coordination sphere” [57, 58, 59, 60, 61]. Although not as powerful as “first‐coordination sphere” interactions, these interactions can nevertheless affect the electronic configuration and molecular orbitals used by the reactive high‐valent oxoiron(IV) species. Furthermore, noncovalent interactions like electrostatic and hydrogen bonding are essential for proper arrangement of the oxidant and substrate in the transition states of enzymatic processes [56, 62, 63].

Motivated by these reports and aiming to more accurately replicate the structures and functions of the enzymatic systems, synthetic chemists have developed strategies to utilize various inherent functionalities in the ligand backbone to integrate secondary coordination sphere effects into the design of synthetic nonheme oxoiron(IV) complexes [64, 65, 66, 67, 68]. Extensive studies from the Borovik group have shown how H‐bonding or electrostatic interactions can affect the stability and reactivity of synthetic high‐spin oxoiron(IV) model complexes [33, 35, 69]. In addition, multiple reports have found the presence of secondary coordination sphere groups to alter reaction pathways, and selectively favor one particular iron‐oxygen intermediate over another [70, 71, 72, 73]. Thus, similar to enzymatic reactions, second coordination sphere interactions may also be significant in regulating the reactivity and selectivity of these synthetic nonheme oxoiron(IV) complexes in the course of substrate oxidation [74, 75, 76, 77, 78, 79].

Recently, we identified a series of nonheme Fe(IV)═O complexes supported by (R‐CH_2_)_3_NTB ligands with a range of differently sized cycloalkyl R groups (Scheme 1, bottom left). We were surprised to find that changing the ring size of the different cycloalkyl groups in the second coordination sphere affects the reactivity of the Fe^IV^═O complexes with hydrocarbon substrates with a range of C─H bonds by controlling the entropy of activation [53].

**SCHEME 1:**
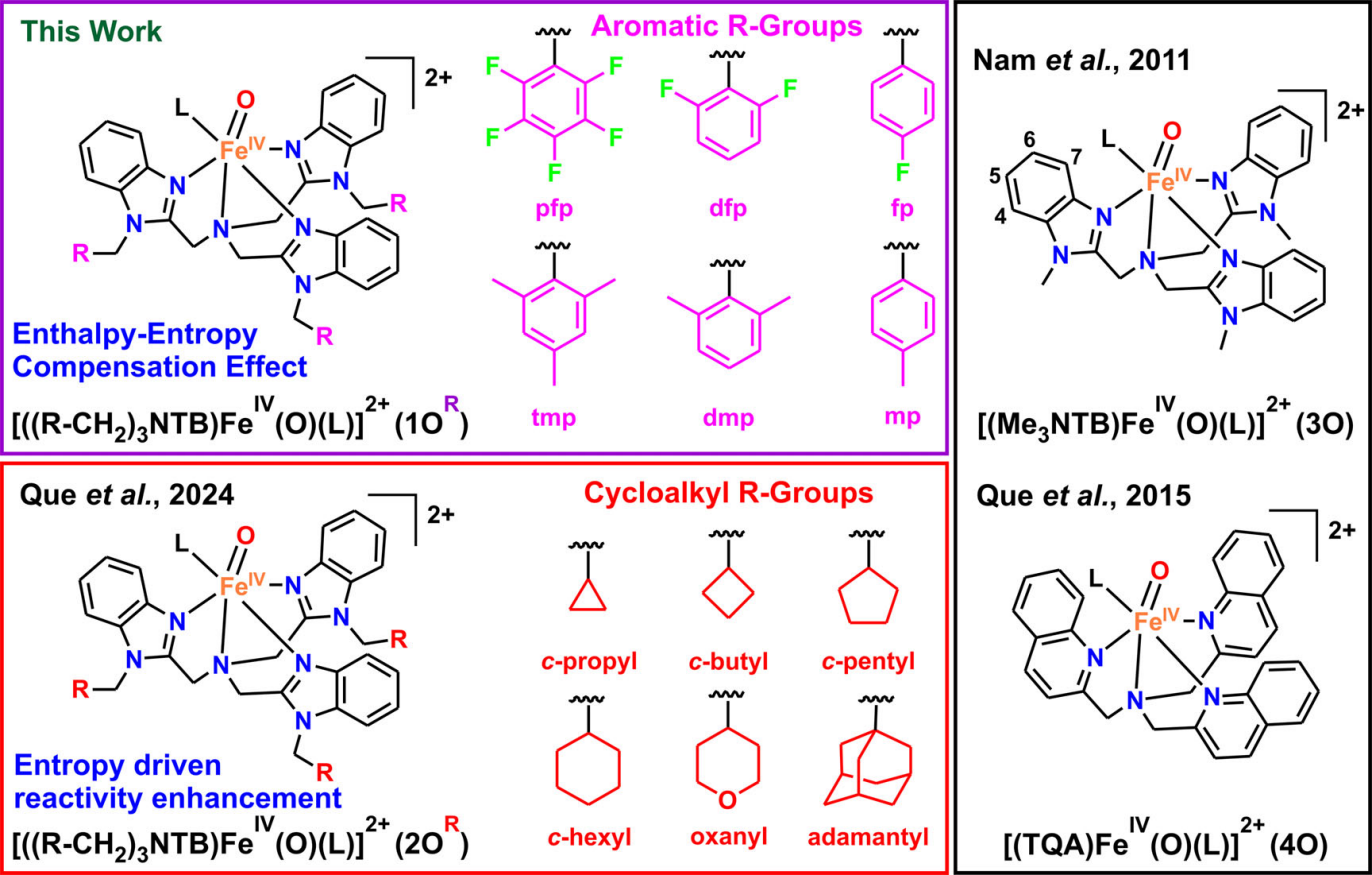
Nonheme oxoiron(IV) complexes discussed in this work (L represents the acetonitrile solvent). The labeling protocol for protons on the benzimidazole rings used is shown on **3O**.

Here, we have extended the series of R‐groups in the second coordination sphere and have assembled additional Fe^IV^═O complexes with aromatic substituents offering different electronic environments with the general formula of [Fe^IV^(O){(R‐CH_2_)_3_NTB} (NCMe)]^2+^ (**1O^R^**, Scheme 1, top left). The reactivity of these Fe^IV^═O complexes with an identical primary coordination sphere is impacted by the R groups, while maintaining their *S* = 1 ground states. Interestingly, we observe different behavior for electron‐poor and electron‐rich substituents. This provides even more compelling evidence for second sphere alterations in ligand geometry that can tune thermodynamic parameters to control reactivity without electronic consequences.

## 2 Results

### 2.1 Synthesis and Characterization of Iron(II) Complexes and corresponding Fe^IV^=O Intermediates

Microcrystalline solids of the precursor iron(II)‐triflate complexes have been generated by reacting iron(II) salt (Fe(OTf)_2_·2CH_3_CN) with their respective ligands in MeCN solvent at ambient temperature under an inert environment (for details, see Experimental Section, Supporting Information). Of the six newly synthesized complexes, diffraction‐quality single crystals have been found for **1^dfp^** (Figure 1; Tables S2 and S3), where the mononuclear iron center is found to be coordinated by two solvent molecules and four nitrogen atoms of the tripodal ligand in a pseudo‐octahedral coordination, unlike most crystal structures of iron(II) complexes with the tris‐benzimidazole‐based supporting ligands that showed a trigonal bipyramidal geometry (Table S4) [51, 53]. Each iron(II)‐precursor complex in this new series of molecules is characterized by standard spectroscopic techniques such as ESI‐mass spectra (positive ion mode), and ^1^H and ^19^F NMR spectra (Figures S1–S3 and Table S1). Solution state Mössbauer spectra obtained from frozen samples of all the iron(II) complexes in this series in MeCN at 4.2 K exhibit isomer shifts (*δ*) values of 1.1‐1.2 mm/s and quadrupole splitting (|Δ*E*_Q_|) of 2.4–2.9 mm/s, typical of pure high‐spin iron(II) species (Figure S5). Based on various spectroscopic data described above for these iron(II) complexes, the electronic properties of the [Fe^II^{(R‐CH_2_)_3_NTB}(CH_3_CN)_2_](OTf)_2_ complexes would appear to be unaffected by the different electronically tuned aromatic R groups. However, slight modifications of the structural parameters in the solution state might be caused by these substitutions, as seen previously for a series of cycloalkyl substituted NTB systems [53].

**FIGURE 1:**
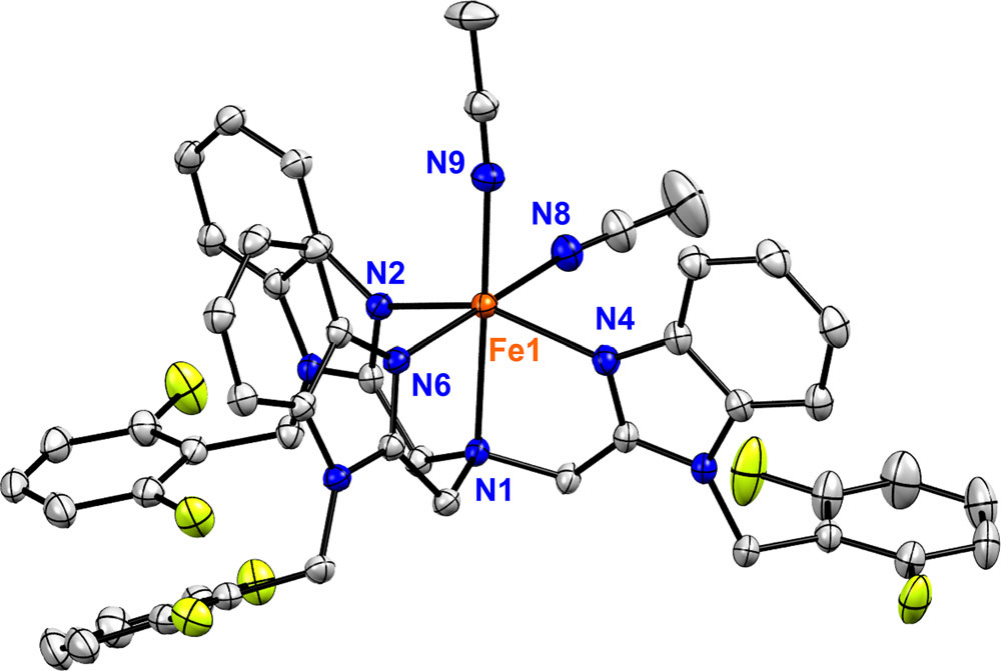
ORTEP plots of complex cation of **1^dfp^** with 40% thermal ellipsoid parameters; all H‐atoms have been omitted for clarity.

The corresponding oxoiron(IV) species are formed upon the reaction of the precursor iron(II)‐complexes in acetonitrile with 2‐(*^t^* BuSO_2_)‐C_6_H_4_IO (*s*‐ArIO) in either 2,2,2‐trifluoroethanol (TFE) or dichloromethane‐*d*_2_ at 233K. To evaluate the impact of the various aromatic substitutions on the NTB ligand backbone, the physical characteristics of the six new oxoiron(IV) complexes have been compared. An intermittent green species with an absorption band attributed to the ligand‐field transition characteristic of a nonheme oxoiron(IV) species [80] is produced instantly upon addition of an oxidant to the iron(II) precursor complex for all six novel complexes (Figure 2) in this series of oxoiron(IV) complexes with various electronically tuned aromatic substituted NTB ligands (**1O^R^**). The absorption bands show *λ*_max_ values within a narrow range of 764–774 nm, similar to that of the parent [(Me_3_NTB)Fe^IV^(O)]^2+^ complex [51] and the related **2O^R^** complexes [53] (Figure 2 and Table S5), which shows that the electronic structure of the iron‐oxo unit and its immediate surroundings remain mostly unchanged across either the **1O^R^** and **2O^R^** series. However, the absorption bands for the oxoiron(IV) complexes diminish progressively over time in different ways (Figures 2 and Figures S5–S10): the half‐life of the complexes ranges from 30 s for **1O^mp^** to 219 s for **1O^pfp^** (see Table S5). Hence, the stability within this series appears to be affected by variations in aromatic substituents in the ligand backbone (Figure 2).

**FIGURE 2:**
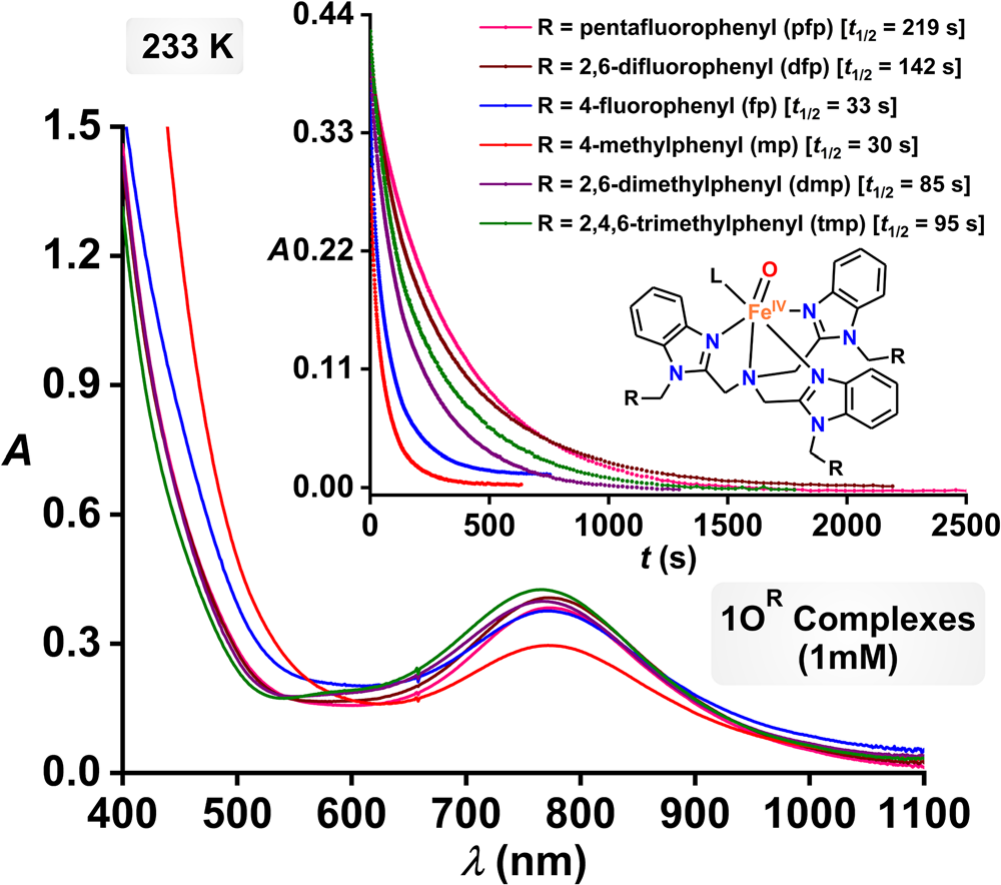
Electronic spectral features at 233 K of 1 mM solutions of Fe(IV) = O complexes **1O^pfp^** (pink), **1O^dfp^** (wine), **1O^fp^** (blue), **1O^mp^** (red), **1O^dmp^** (purple), and **1O^tmp^** (green) generated in acetonitrile upon treatment of the iron(II) precursors with *s*‐ArIO. Inset: time traces for the bands at their corresponding *λ*_max_ values.

Mössbauer spectroscopy has been used to characterize the oxo‐iron(IV) complexes in MeCN at 4.2 K using ^57^Fe‐enriched samples in a 0.1 T magnetic field to learn more about their spin states and electronic environments. Like **3O**, **1O^pfp^** exhibits a quadrupole doublet (51%) with *δ* (isomer shift) = 0.05 mm/s and |Δ*E*_Q_| (quadrupole splitting) = 1.52 mm/s, consistent with an *S* = 1 oxoiron(IV) complex (Figure S11) [27, 31]. In fact, the Mössbauer quadrupole doublets of complexes **1O^dfp^**, **1O^fp^**, **1O^tmp^**, **1O^dmp^**, and **1O^mp^** show quite comparable isomer shifts and quadrupole splitting values at 4.2 K (Table S5 and Figures S12–S16). Moreover, the Mössbauer parameters for the (aromatic substituted) **1O^R^** series are very similar to those observed for the (cycloalkyl substituted) **2O^R^** series [53] (Table S5). It should also be mentioned that signals for mononuclear ferric or diferric species can be found in every instance, likely resulting from the decay of the nascent oxoiron(IV) complex (Figures S11–S16).

To further check the spin‐state of this **1O^R^** series in solution, ^1^H NMR data have been collected at 193 K and compared with the proton NMR signals of previously reported NTB‐based oxoiron(IV) complexes (**2O^R^** and **3O**, the Me_3_NTB parent ligand). For the **1O^R^** complexes in solution, ^1^H NMR data at 193 K reflect an iron center with an exclusive *S* = 1 intermediate spin state. Consequently, an alteration in spin state cannot be the cause of the notable change in stability exhibited by this series of novel compounds. Similar to the other NTB‐based oxoiron(IV) complexes (**2O^R^** and **3O**) [53, 81], the paramagnetically shifted ^1^H NMR spectra of these complexes show seven distinct features for the benzimidazole protons in the 0–25 ppm region (Figures 3 and Figures S30–S35; Table S9), which indicates that the molecules in this series have six‐coordinate structures with bound solvent as the main species. While the proton signals corresponding to the benzimidazole H4 and H5/H6 protons fall within a common region in their ^1^H‐NMR spectra (Table S9), they exhibit slightly different splitting patterns (Figure 3) that show the effect of the different *R* groups on the environments of the benzimidazole protons.

**FIGURE 3:**
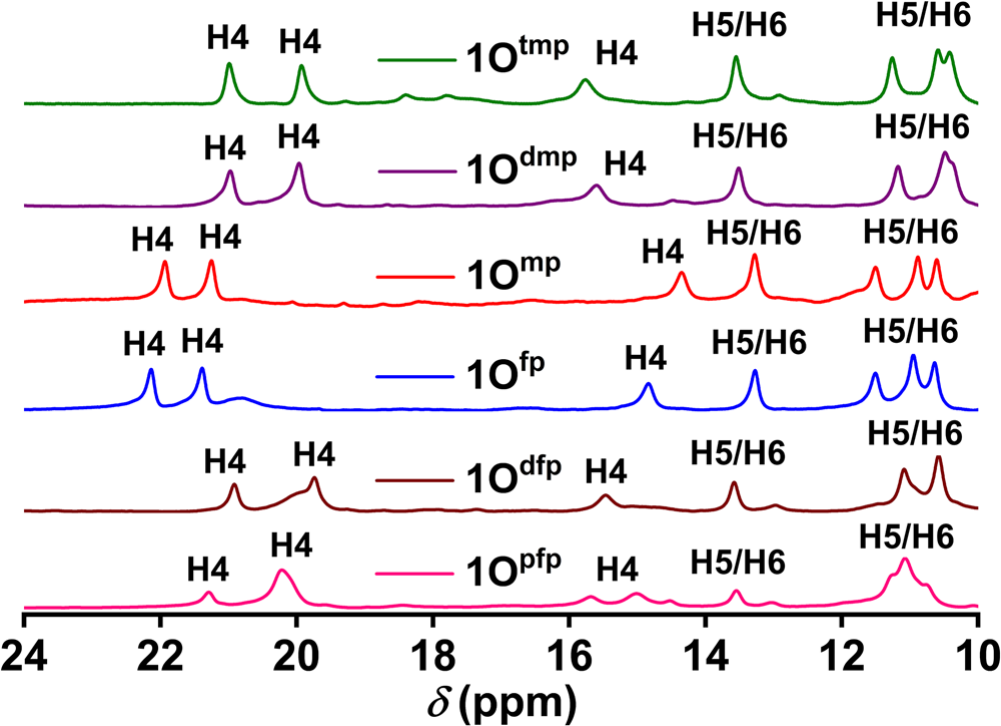
The ^1^H NMR spectra of **1O^R^** complexes (24 to 10 ppm) in acetone‐*d*_6_: acetonitrile‐*d*_3_ (9:1) mixture at 193 K.

An examination of the ^1^H NMR data of the **1O^R^** complexes clearly identifies the H4 proton signals of the benzimidazole heterocycles as the most dramatically influenced (Figure 3). Shifts in the H4 proton signal can be readily observed to correlate with substrate oxidation rate of the corresponding NTB‐based oxoiron(IV) complexes (Figures S36, 37). A similar trend has also been found for the recently reported series of oxoiron(IV) complexes with cycloalkyl substituted NTB systems (Figures S36 and S37) [53, 81]. Note that the shift in the H4 proton signals are more dramatic for the aromatic substituted NTB‐supported oxoiron(IV) complexes than for its cycloalkyl‐substituted counterparts. However, it is worth noting that **2O^adamantyl^** from the **2O^R^** series and **1O^pfp^** from the **1O^R^** series behave somewhat differently than the other complexes in each series. This variation might result from its more intricate structure resulting from the **2O^adamantyl^**’s larger adamantyl group and **1O^pfp^**’s bigger pentafluorophenyl group compared to the other substituents, making them more rigid in low‐temperature solutions used for this study.

### 2.2 Reactivity

The oxidative potential of this new series of oxoiron(IV) complexes toward hydrogen atom transfer (HAT) processes at 233 K has been evaluated using substrates with C─H bond dissociation energies (BDEs) ranging from 87.0–99.3 kcal/mol. Substrate addition to MeCN solutions of **1O^R^** complexes at 233 K shows accelerated pseudo first‐order decay of the absorption bands of the oxoiron(IV) chromophore vs self‐decay (Figures S17–S22). Values of the second‐order rate constants (*k*_2_) for these oxidations are derived from the linear dependence of the decay rate of the oxoiron(IV) chromophore absorption band versus substrate concentration (Figures S23–S28). Despite having similar electronic characteristics, the second‐order rate constant for **1O^R^** complexes show a clear spread in their reactivity values (Figure 4 and Table S6), which span close to an order of magnitude.

**FIGURE 4:**
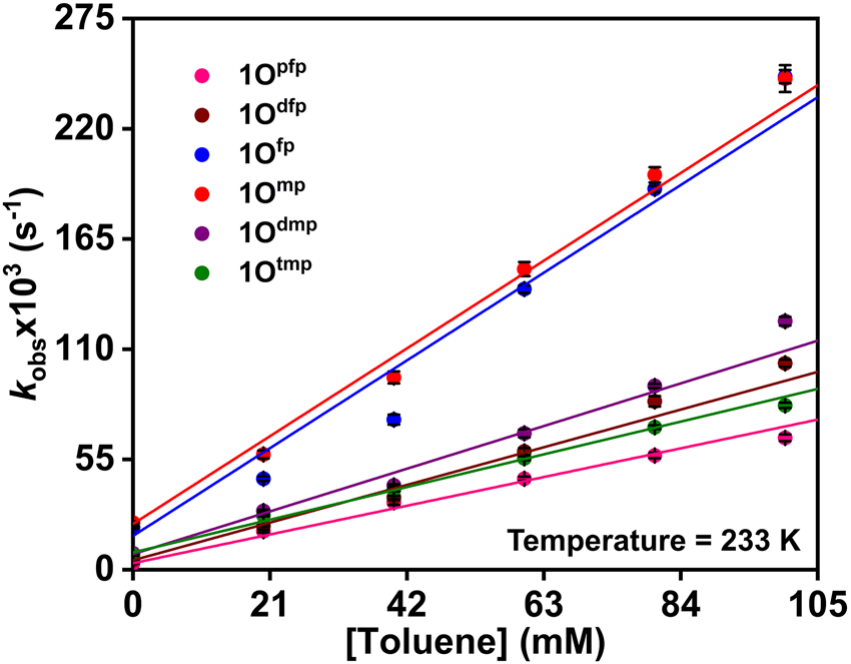
Plot of *k*_obs_ (at 233 K) vs toluene concentration for reactions with **1O^pfp^** (pink line), **1O^dfp^** (wine line), **1O^fp^** (blue line), **1O^mp^** (red line), **1O^dmp^** (purple line), and **1O^tmp^** (green line). Error bars represent ± SD (𝑛 = 3).

Comprehensive reactivity analyses indicate that these *S* = 1 **1O^R^** complexes exhibit greater reactivity than the previously documented reactivity for the parent *S* = 1 **3O** complex [51] as well as the previously most reactive synthetic nonheme *S* = 2 [(TQA)Fe^IV^(O)(CH_3_CN)]^2+^ complex [37] (**4O**) (Table S6). The strengths of the C─H BDE values are found to be correlated linearly with log*k*_2_' for this **1O^R^** series (*k*_2_' is the observed *k*_2_ value at 233 K divided by the number of identical C─H bonds in a substrate, e.g., 12 for cyclohexane); hence, the reaction rates are dictated by the C─H bond that is cleaved (Figure 5). In most cases for a specific substrate, the reaction rate at 233 K increases in the following order: **1O^pfp^** ∼ **1O^tmp^** < **1O^dfp^** ∼ **1O^dmp^** < **1O^fp^** ∼ **1O^mp^**, as shown in Figure 5. The two most reactive complexes in the series, **1O^fp^** and **1O^mp^**, show ethylbenzene oxidation rates that are five‐fold and seven‐fold faster than **3O** and four‐fold faster than **4O**, respectively. With the more inert cyclohexane substrate, **1O^fp^** and **1O^mp^** respectively react four‐fold and three‐fold faster than **3O** and **4O**.

**FIGURE 5:**
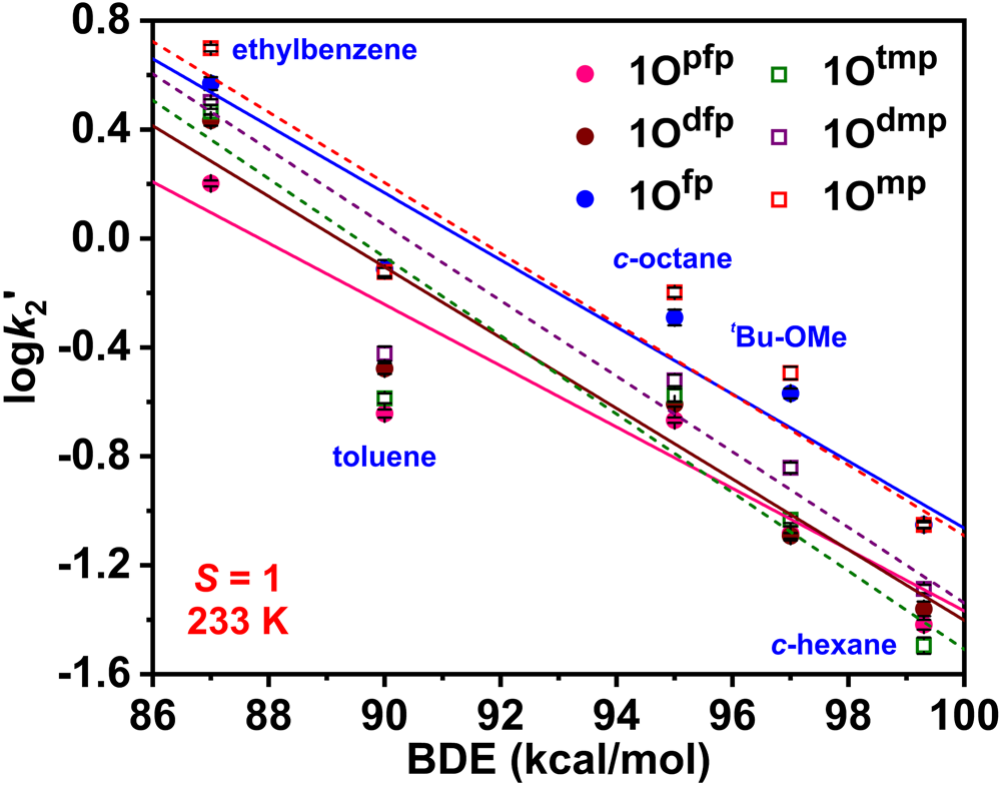
Plot of log*k*_2_’ (at 233 K) vs. the C─H BDE of substrates in the reactions of **1O^pfp^** (pink solid line), **1O^dfp^** (wine solid line), **1O^fp^** (blue solid line), **1O^tmp^** (green dash line), **1O^dmp^** (purple dash line) and **1O^mp^** (red dash line) with various substrates. The *k*_2_’ values are calculated from the second‐order rate constant of substrate oxidation reactions (*k*_2_). The *k*_2_ values for the entire series of substrates from Figures S23–S28 are listed in Table S6 (error bars are also listed in Table S6). Error bars represent ± SD (𝑛 = 3).

To check the kinetic isotope effect for the reaction of this series of oxoiron(IV) molecules, reactivity studies of these molecules have been carried out with deuterated substrate. KIE values for the **1O^R^** have been obtained by direct comparison of second‐order rate constant values for toluene and toluene‐*d*_8_ oxidation reactions of the corresponding oxoiron(IV) complexes. For complexes **1O^pfp^**, **1O^dfp^**, **1O^fp^**, **1O^tmp^**, **1O^dmp^**, and **1O^mp^**, the corresponding KIE values are 28, 26, 25, 26, 19, and 28, respectively (Figure S29), suggesting the existence of quantum tunneling effects as indicated by these high non‐classical KIE values [51, 82]. These results also point to the C─H bond breaking as the rate‐determining step in the HAT reactions involving this **1O^R^** series. We also analyzed the reaction mixture post‐reaction with cyclohexane and obtained a sub‐stoichiometric quantity of products (both cyclohexanol and cyclohexanone, see Table S8). No notable alteration in yield or selectivity (alcohol vs. ketone) has been detected for this **1O^R^** series of compounds.

A more comprehensive view could possibly be obtained by comparing the oxidative capabilities of the most reactive systems of the new series of molecules (**1O^fp^** and **1O^mp^**) with those of previously reported for other synthetic oxoiron(IV) complexes (Table S7). Reactivity studies demonstrate that **1O^fp^** and **1O^mp^** are approximately 6,000x more reactive for cyclohexane oxidation than [Fe^IV^(O)(TPA)(CH_3_CN)]^2+^ [83], the first reported oxoiron(IV) complex in the tripodal ligand family. Compared to the reactivity of the most reactive molecule of the N4Py family of ligands, that is, [Fe^IV^(O)(N2Py2Pz)]^2+^ complex [84], the cyclohexane oxidation rate of **1O^fp^** and **1O^mp^** are approximately 350 times higher. Similarly, upon comparing the cyclohexane oxidation rate of **1O^fp^** and **1O^mp^** with the most reactive oxoiron(IV) complex of the family of macrocyclic ligands, that is, [(TMCO)Fe^IV^(O)(OTf)]^+^ complex [85], we observe that the new complexes react around 50 times faster at 233 K than the latter. In addition, **1O^fp^** and **1O^mp^** react with toluene ∼5x more rapidly than [(*^t^* Bu_3_tacn)Fe^IV^(O)]^2+^, thus far the sole documented instance of an *S* = 2 pseudo‐tetrahedral Fe^IV^═O complex [36]. However, the **1O^R^** series is less reactive than the [Fe^IV^(O)(L1‐bispidine)(Cl)]^+^ complex [54, 55], the most reactive oxoiron(IV) complex from the bispidine ligand family. In contrast, the acetonitrile‐bound analogue of the same molecule, [(L1‐bispidine)Fe^IV^(O)(CH_3_CN)]^2+^ [55, 86], exhibits significantly slower reactivity than **1O^fp^** and **1O^mp^**, according to the reported reactivity data. A comparison with the reactivity of **2O*^c^* ^‐hexyl^**, the most reactive cycloalkyl‐substituted NTB oxoiron(IV) complex [53], reveals that **1O^fp^** and **1O^mp^** exhibit a *k*_2_ value for cyclohexane oxidation approximately 60% that of **2O*^c^* ^‐hexyl^**. In addition, **1O^fp^** and **1O^mp^** oxidize 1 M cyclohexane at a rate of 1.07 s^−1^ at 233 K, twice as fast as the rate for taurine oxidation of TauD‐*J* [87] after temperature correction.

## 3 Discussion

We investigated a possible correlation between the reactivity of the six new complexes with the electronic nature of the substituents used. However, no linear correlation is found for the **1O^R^** series with different substrates (Figure S44). As one switches from **1O^fp^** to **1O^dfp^** to **1O^pfp^**, the substituents become progressively more electron‐poor due to the increasing number of fluorine atoms on the phenyl ring, which leads to a decrease of the oxidative potency (**1O^fp^** most reactive, **1O^pfp^** least reactive). On the other hand, going from **1O^mp^** to **1O^dmp^** to **1O^tmp^** leads gradually to more electron‐rich substituents. If the electronic tuning of the phenyl were to dictate reactivity, one would have anticipated that reactivity will progressively improve from **1O^mp^** to **1O^dmp^** to **1O^tmp^**. However, the reactivity observed in fact decreases gradually from **1O^mp^** to **1O^dmp^** to **1O^tmp^** (Figure S44). Furthermore, **1O^mp^** and **1O^fp^** exhibit nearly identical reactivity, despite having substituents with an electron‐rich 4‐methylbenzyl group or an electron‐poor 4‐fluorobenzyl group. Hence, the reactivity is not determined by the electronic properties of substituents on the aromatic ring in the secondary coordination sphere. Even more unexpectedly, the reactivity decreases both if we make the aromatic rings more electron‐poor and if we make them more electron‐rich.

We have performed temperature‐dependent kinetic studies (193–233K) to obtain activation parameters for the toluene oxidation reactions with all six oxoiron(IV) intermediates, as derived from the decay rates in Eyring plots (Figures S38–S43). This produces a positive Δ*H* ^‡^ and a negative Δ*S* ^‡^ for all complexes in the **1O^R^** series, which confirms that the transition state is associative in nature (Table S10). From the Δ*H* ^‡^ and Δ*S* ^‡^ values we obtain the corresponding activation free energy (Δ*G* ^‡^) with toluene at 233K (Table S10). Despite the modest difference in activation free energy values, the Δ*G* ^‡^ values start to decrease from **1O^dfp^** in the following order: **1O^dfp^** ∼ **1O^tmp^** ∼ **1O^dmp^** > **1O^pfp^** > **1O^fp^** > **1O^mp^**. This has been confirmed by our DFT barriers (Table S12) that corroborate these trends for **1O^fp^**, **1O^dfp^**, **1O^mp^**, and **1O^dmp^**, with **1O^fp^** and **1O^mp^** being most reactive. Furthermore, we have computed the Δ*G* ^‡^ values from the *k*_2_ values using the following equation [88]:

(1) $$\;\Delta G^{‡}=\left[ln\left(\kappa \frac{k_{B}}{h}T\right)-\ln\left(k_{2}\right)\right]\;\times \mathrm{RT}$$

The obtained Gibbs free energy values from eq. 1 (Table S10) closely match the Eyring‐determined Δ*G* ^‡^ values, exhibiting nearly the same pattern. The only exception is **1O^pfp^** that shows a 1.0 kJ mol^−1^ larger Δ*G* ^‡^ value from eq. 1 when compared to the corresponding Eyring‐determined Δ*G* ^‡^ value.

We can now combine the data for the **1O^R^** and **2O^R^** [53, 81] series, which are all supported by the same primary coordination sphere but differ only in the nature of the secondary sphere R groups attached to the benzimidazole moiety. We have plotted the *k*_2_ value at 233K for toluene oxidation against their Eyring derived Δ*G* ^‡^, Δ*H* ^‡^, and Δ*S* ^‡^ values for all complexes (Figure 6a–c). To our surprise, we observed for *k*_2_ vs Δ*G* ^‡^ that these show two different linear relationships, and that the **1O^R^** series deviates more from linearity (lower R^2^ value). Interestingly, no good linear correlation between *k*_2_ and Δ*H* ^‡^/Δ*S* ^‡^ covering all complexes within the **1O^R^** series has been observed. Instead, we find two sub‐groups of the **1O^R^** series, one involving complexes with an electron‐poor group (EPG), and the other with an electron‐rich group (ERG). The slopes for the EPG and ERG sub‐groups are completely opposite: the Δ*H* ^‡^/Δ*S* ^‡^ of EPGs show a positive slope (similar to **2O^R^**), while ERGs show a negative slope. Furthermore, the slopes are larger for **1O^R^** series than for **2O^R^** series; the latter are much more constant.

**FIGURE 6:**
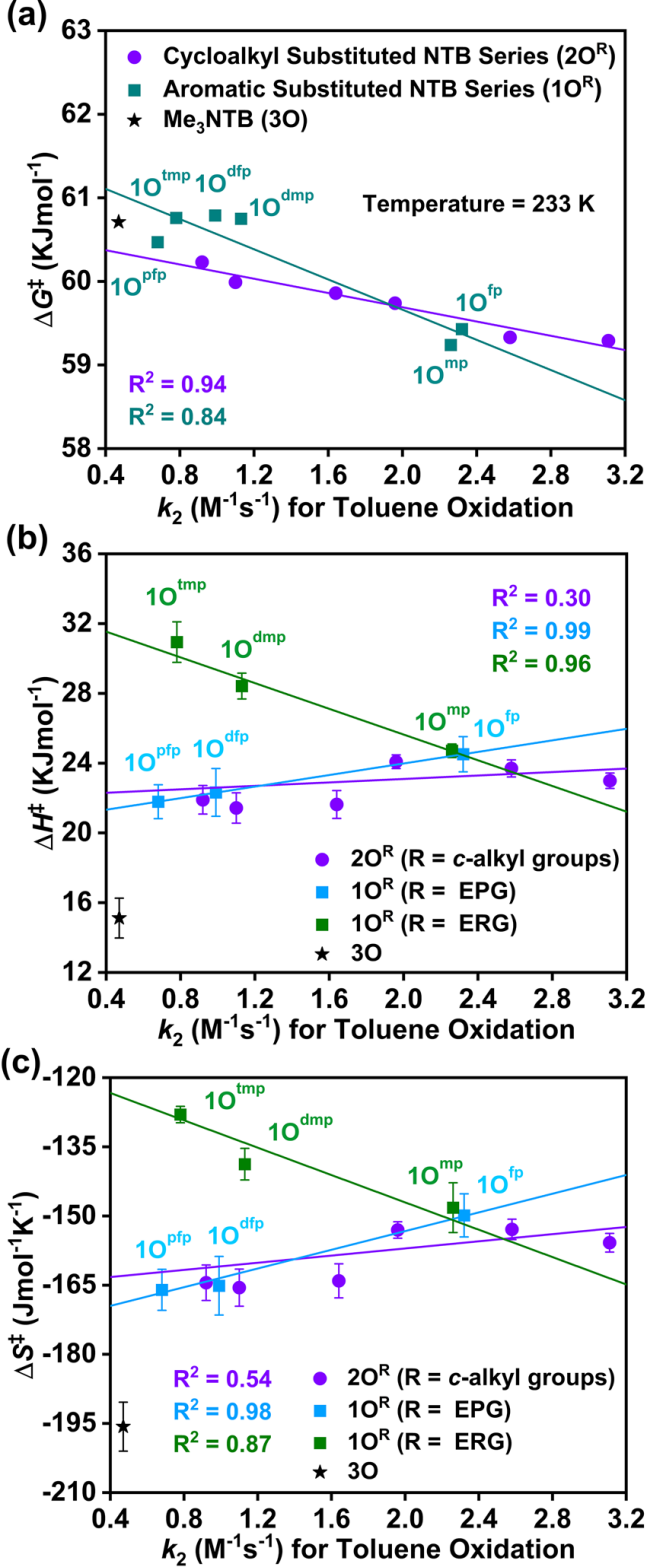
Correlation of Eyring plot derived thermodynamic parameters of the toluene oxidation reaction [(a) Δ*G* ^‡^, (b) Δ*H* ^‡^ and (c) Δ*S* ^‡^] for the series of [{R‐CH_2_)_3_NTB}Fe^IV^(O)(L)]^2+^ complexes with the toluene oxidation rates of the corresponding oxoiron(IV) complexes. Error bars represent ± SD (𝑛 = 3).

These correlations indicate that in the **1O^R^** series the electronic features of the substituent influence the activation enthalpy and entropy of the substrate oxidation reaction more strongly, hence directly regulating the substrate oxidation rates. Therefore, we explored the “Enthalpy‐Entropy Compensation Effect” (EECE) for our **1O^R^** series. Within biochemistry, this EECE has been widely debated [89, 90, 91, 92, 93, 94] and disputed [95, 96, 97] but within the bioinorganic literature it has received not so much attention, until recently [98, 99, 100]. The first comprehensive study on EECE in biomimetic oxidation processes was presented [100] in 2021 by Nam and coworkers on a series of oxo‐Mn(IV) complexes supported by electron‐rich and electron‐poor porphyrins, where it was found that different reaction temperatures can cause the reactivities of electron‐rich and electron‐poor Mn(IV)‐oxo porphyrins to flip. They discovered a linear connection between Δ*H* ^‡^ and Δ*S* ^‡^, which allowed them to determine a compensation temperature, T*_comp_*, where the reactivities of complexes with EPGs and ERGs were almost ideally equal.

Following the EECE approach we have plotted Eyring‐derived Δ*H* ^‡^ and Δ*S* ^‡^ values (Table S10) against each other (Figure S45). Remarkably, we observe a consistent linear correlation between Δ*H* ^‡^ and Δ*S* ^‡^ values in the reactions of all six oxoiron(IV) complexes in the **1O^R^** series:

(2) $$\Delta H^{‡}=0.243\Delta S^{‡}+61.293$$

With the regular Gibbs free energy equation (Δ*G* ^‡^ = Δ*H* ^‡^ − TΔ*S* ^‡^), and some rearrangements, we can determine the relative activation Gibbs energy (ΔΔ*G* ^‡^), which now depends only on enthalpy:

(3) $$\;\Delta \Delta G^{‡}=\left(1-4.115\times 10^{-3}T\right)\;\Delta \Delta H^{‡}$$

This equation shows that a value of zero for ΔΔ*G* ^‡^ is obtained at a temperature of 243 K (‐30°C), which is known as the “compensation temperature” (T_comp_). That is, at this temperature changes in enthalpy cancel out differences in entropy, making the rate constant values totally invariant [98, 100]. Consequently, at 243K the reactivities of all **1O^R^** complexes, both with electron‐rich and electron‐poor groups, are essentially identical. Moreover, if T>T_comp_, then ΔΔ*G* ^‡^ <0, and the ERG subgroup of **1O^R^** would be more reactive than the EPG subgroup of **1O^R^**; vice versa for T<T_comp_, ΔΔ*G* ^‡^ >0, and the EPG subgroup would exhibit greater reactivity than the ERG subgroup of **1O^R^**. Nevertheless, this does not concur with our data at 233 K: based on EECE one would expect the EPG subgroup to have greater reactivity (because of the smaller Δ*H* ^‡^, Table S10), but in fact **1O^dfp^** (EPG) is slightly less reactive than **1O^dmp^** (Δ*G* ^‡^ 60.79 kJ mol^−1^ vs. 60.75 kJ mol^−1^). It can be reconciled by considering T_comp_ for pairs of complexes in the **1O^R^** series: the reactivity of **1O^tmp^** and **1O^pfp^** for the C─H activation of toluene is inverted at a temperature of 241 K (T_cross_, Figure 7a), which is quite close to the calculated T_comp_ (243K). Moreover, at 233 K (Table S10) the electron‐poor **1O^pfp^** has a smaller Gibbs free energy of activation than electron‐rich **1O^tmp^**, which is consistent with the EECE. The same is true for **1O^dmp^** and **1O^dfp^**, for which we find a T_comp_ of 231 K (Figure 7b). Since this value is lower than the 233 K of our experiments, according to EECE the electron‐rich **1O^dmp^** is expected to be (a little) more reactive than the electron‐poor **1O^dfp^**, which is indeed the case. Surprisingly, no crossover in reactivity has been observed for **1O^mp^** and **1O^fp^** in the range of 193–243 K (Figure 7c). Moreover, this pair exhibits a Δ*H* ^‡^/Δ*S* ^‡^ relation that does not fall exactly on the line of Figure S45. Instead, the Δ*H* ^‡^ as predicted from equation 2 is 0.8‐1.0 kJ mol^−1^ larger (25.3 and 25.7 kJ mol^−1^, respectively, for **1O^fp^** and **1O^mp^**) than in fact observed from the Eyring‐data (Table S10).

**FIGURE 7:**
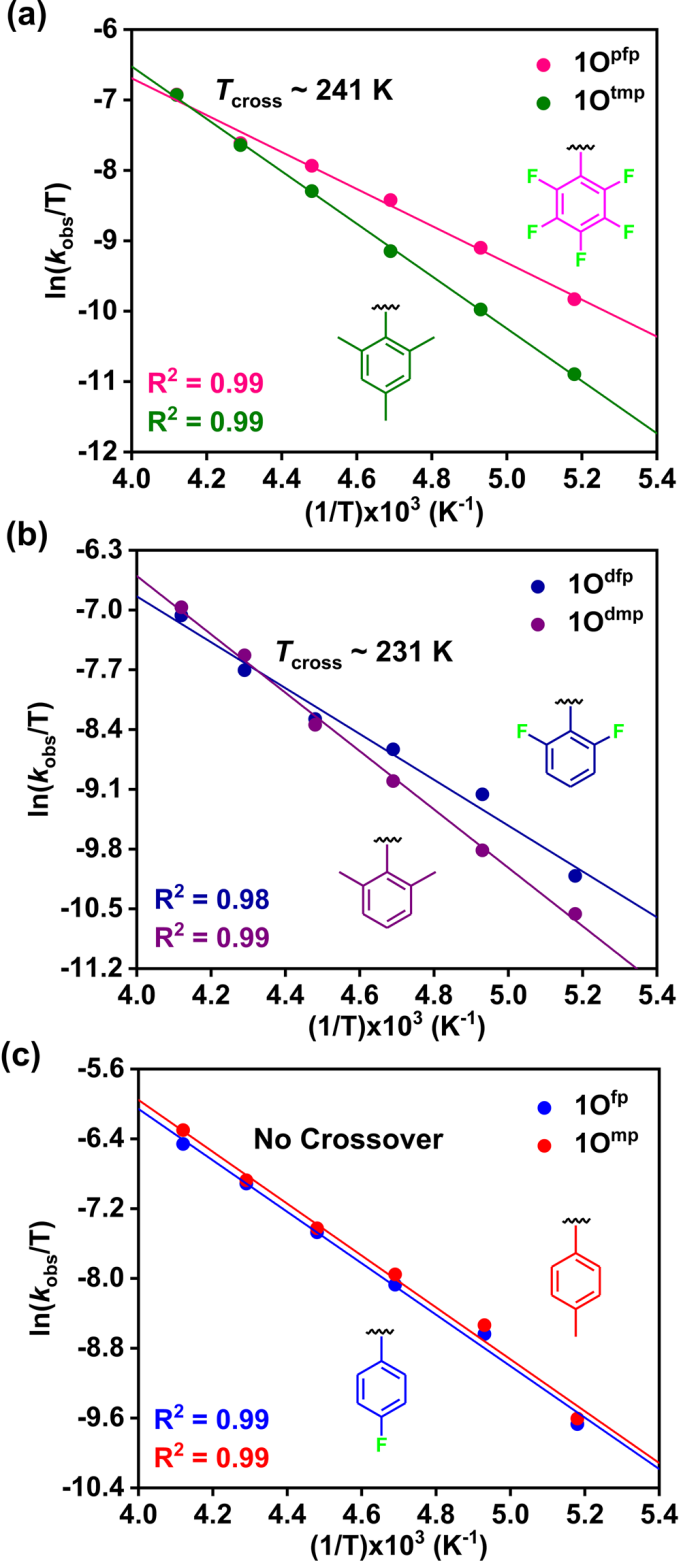
Comparison of Eyring plots for the oxidation reactions of toluene by (a) 1O^pfp^ and 1O^tmp^, (b) 1O^dfp^ and 1O^dmp^, (c) 1O^fp^ and 1O^mp^.

To assess the broad application of EECE in biomimetic oxoiron(IV) chemistry, we plotted the Eyring‐derived Δ*H* ^‡^ and Δ*S* ^‡^ values for the toluene oxidation reaction (233 K) against each other for the **1O^R^** and **2O^R^** series as well as parent **3O**. Incredibly, the following equation (4) was derived for the 13 oxoiron(IV) complexes of NTB based supporting ligands, where the Δ*H* ^‡^ and Δ*S* ^‡^ values display a linear association (Figure 8).

(4) $$\Delta H^{‡}=0.224\Delta S^{‡}+58.231$$

**FIGURE 8:**
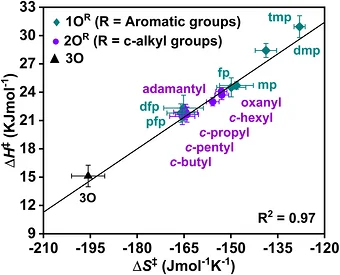
Plot of Δ*H* ^‡^ vs Δ*S* ^‡^ in the C─H activation of toluene by mononuclear nonheme oxoiron(IV) complexes within the NTB ligand family. Error bars represent ± SD (𝑛 = 3).

From the wide range of activation parameter values for all 13 molecules, it should be noted that *c*‐alkyl substituents show less impact on the Δ*H* ^‡^ and Δ*S* ^‡^ parameters when compared to electronically adjusted aromatic substituents. Nonetheless, the linear correlation between Δ*H* ^‡^ and Δ*S* ^‡^ unequivocally indicates that the EECE is applicable to all complexes within this series.

Steric effects of substituents are another potential element that can influence the reactivity. As previously noted, the NMR signals from benzimidazole protons exhibit significant paramagnetic shifts, which could be influenced by the presence of bulky groups in the substituents. Hence, we tried to correlate the observed reactivity of these complexes with the steric properties of the R groups. Figure 9 shows the top view of the computed DFT structures for the six *S* = 1 oxoiron(IV) complexes, when the aromatic rings are in most cases parallel to the Fe = O axis (the most favored orientation is shown, corroborated by the computed Mössbauer parameters, see Table S11).

**FIGURE 9:**
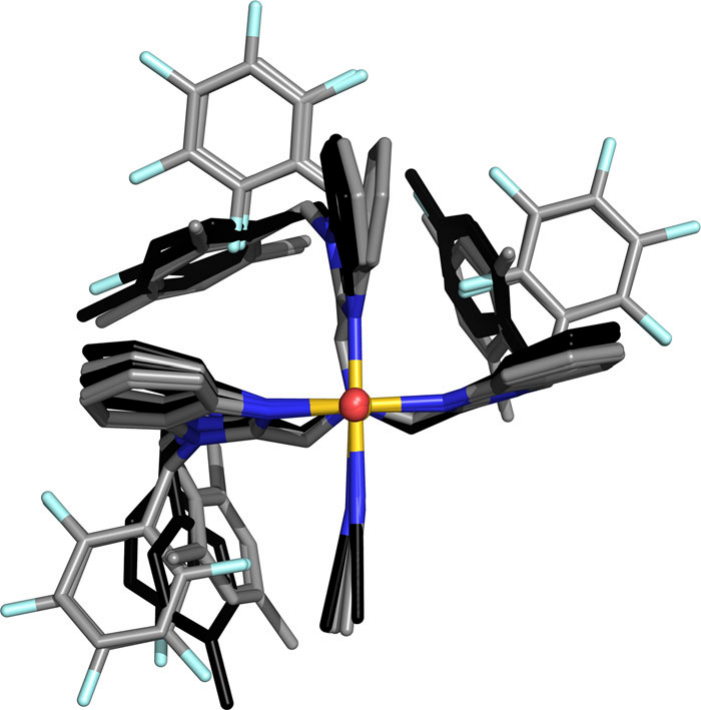
Top view of the DFT structures (obtained at S12g/TZ2P, COSMO/ZORA) for the oxoiron(IV) complexes. Shown with black carbons are the two most reactive complexes, **1O^fp^** and **1O^mp^**, while the less reactive complexes are shown with carbons in light gray.

The two most reactive complexes, **1O^fp^** and **1O^mp^** (shown with carbons in black), are observed to be in the middle (by looking at the benzimidazole trans to MeCN, at the top of the figure), with the four less reactive complexes showing greater distortion toward the left or toward the right. The number of substituents on the aromatic **1O^R^** groups differs among the complexes (Scheme 1), that is, **1O^tmp^** contains three methyls, **1O^pfp^** five fluors, **1O^dmp^** and **1O^dfp^** have methyls or fluors at the 2 and 6 position, and **1O^mp^** and **1O^fp^** have one methyl or fluor at the para(4) position. Hence, the number of substituents in the aromatic rings are 1, 2, 3 for **1O^mp^**, **1O^dmp^**, **1O^tmp^**, and 1, 2, and 5 for **1O^fp^**, **1O^dfp^**, and **1O^pfp^**, respectively. Comparing these numbers to the reactivity shows that the oxidation rates decrease as the number of substituents increases (Figure 10).

**FIGURE 10:**
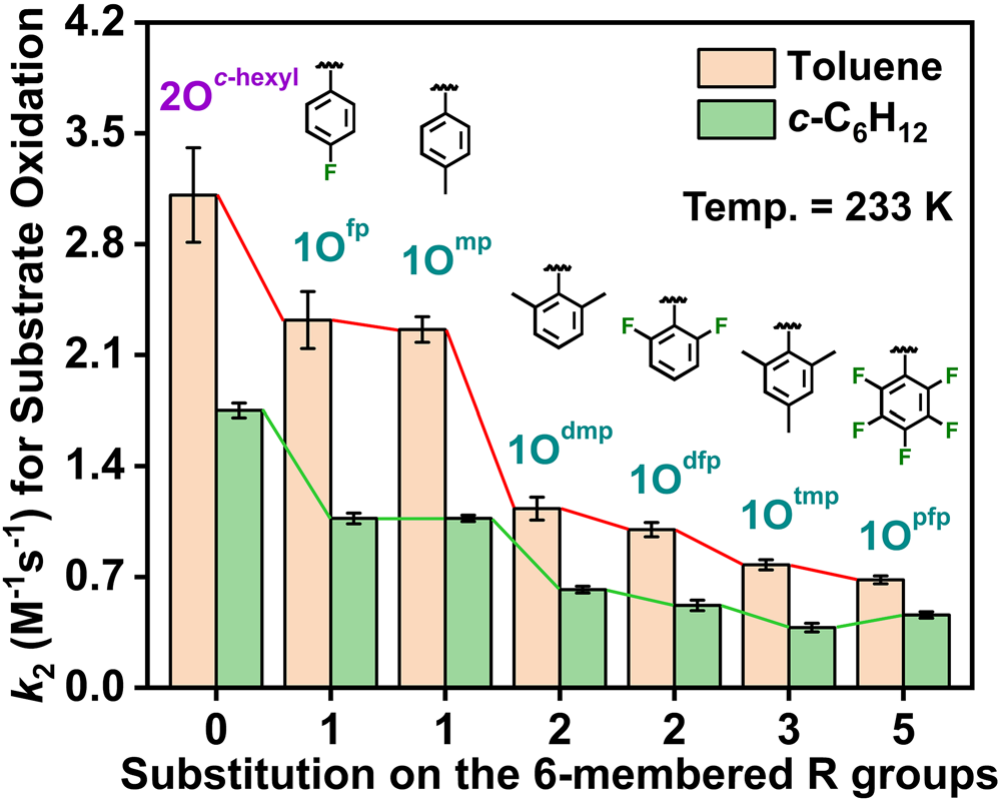
Plot of *k*_2_ for substrate oxidation reactions of the [{R‐CH_2_)_3_NTB}Fe^IV^(O)(CH_3_CN)]^2+^ complexes versus the number of substituents on the 6‐membered R groups in corresponding oxoiron(IV) complex. Error bars represent ± SD (𝑛 = 3).

This observation clearly demonstrates that an increased number of substituents on the aromatic phenyl groups alters the reactivity of these complexes in alkane oxidation. We also note that the reactivity is sensitive to the presence of ortho (2,6) substituents on the phenyls, perhaps due to added steric factors near the Fe═O center. To further understand this, we computed the reaction barriers for the four most reactive **1O^R^** complexes (**1O^fp^**, **1O^mp^**, **1O^dmp^**, **1O^dfp^**) at the S12g/TZ2P level, which corroborated our experimental results (see Figure 11; Tables S11 and S12). The computed barriers are lowest for the para‐substituted aromatic rings (**1O^fp^**, **1O^mp^**), but higher than for the **2O*^c^* ^‐hexyl^** complex [53], while the 2,6‐disubstituted ligands are least reactive. Plotting the computed Δ*H* ^‡^ and Δ*S* ^‡^ parameters (see Figure S46) shows a similar linear trend as seen in Figure 8, although the correlation is a little bit lower because of the largest cycloalkyl substituents rings from our previous study [53], probably due to conformational flexibility of the latter in our calculations.

**FIGURE 11:**
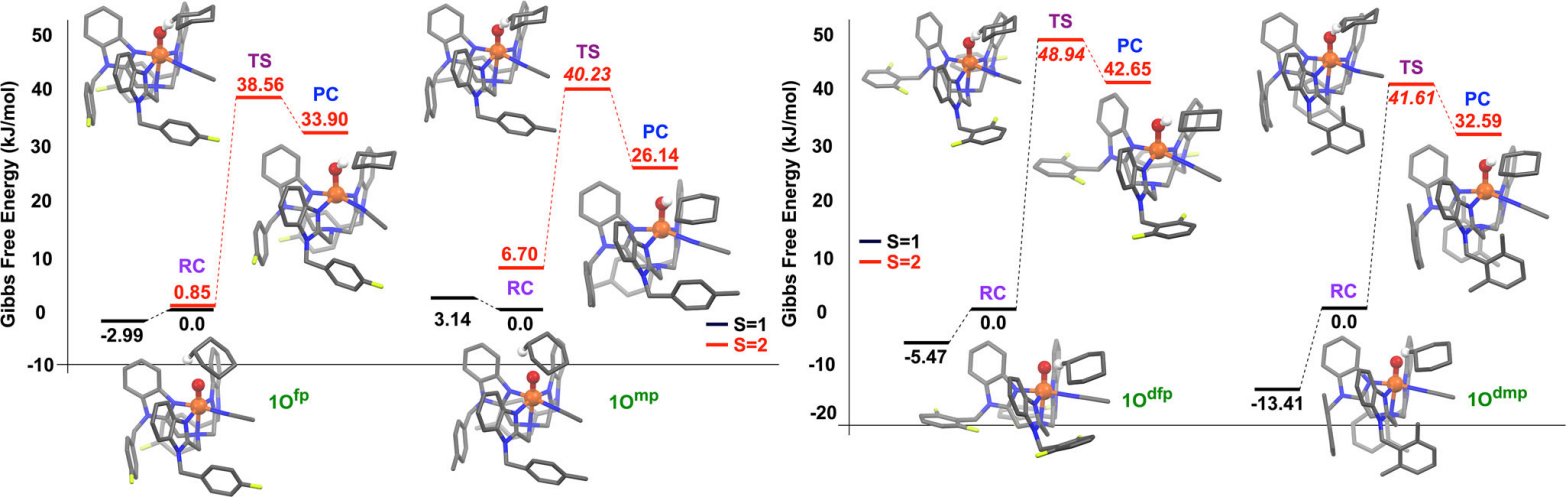
Computed Gibbs free energy profiles (S12g/TZ2P (COSMO, ZORA), kJ·mol^−1^).

## 4 Conclusion

This study illustrates the effects of imbuing the tetradentate tripodal NTB ligand scaffold with various electron‐rich and electron‐poor benzyl groups at the secondary‐sphere 1‐position of the benzimidazole moiety, giving rise to a new series of *S* = 1 oxoiron(IV) complexes that exhibit reactivities that are among the highest observed to date for the oxidation of C─H bonds by synthetic nonheme complexes in the tripodal ligand family. Indeed, the oxidations performed by this family of complexes are faster than those reported for the most reactive *S* = 2 [(TQA)Fe^IV^(O)(CH_3_CN)]^2+^ complex and the parent *S* = 1 [(Me_3_NTB)Fe^IV^(O)(CH_3_CN)]^2+^ complex.

According to our ^1^H‐NMR experiments in acetone solution at 193 K as well as Mössbauer measurements at 4 K, the **1O^R^** complexes maintain the *S* = 1 spin state. A range of spectroscopic measurements indicates that the overall electronic features of all six complexes in the series are quite similar. Nevertheless, the oxidation reaction rates of C─H bond cleavage are shown to differ significantly within this group of compounds. This is corroborated by thermodynamic variables (Δ*H* ^‡^, Δ*S* ^‡^, and Δ*G* ^‡^) for the HAT reaction of **1O^R^** complexes with substrates that correlate with these reaction rates.

Kinetic studies of the C─H activation processes show that the reactivity of electron‐rich and electron‐poor oxoiron(IV) complexes in this series can be turned around depending on the reaction temperatures. This alteration in reactivity can be explained by the EECE. In the C−H activation mediated by [{(R‐CH_2_)_3_NTB}Fe^IV^(O)(CH_3_CN)]^2+^ complexes, a consistent linear relationship between Δ*H* ^‡^ and Δ*S* ^‡^ was observed. This relationship allows the determination of a compensation temperature, T_comp_, where the reactivities are ideally identical. So, the results show that EECE is present in oxidation reactions involving these nonheme oxoiron(IV) complexes.

This study represents the first comprehensive investigation of EECE for a series of oxoiron(IV) complexes with electronically tuned groups in the second coordination sphere of the supporting ligand that we are aware of. In addition, this study also reveals the effect of sterics on these R groups in tuning the rates of substrate oxidation reactions by **1O^R^** complexes. This series of complexes highlights the significance of slight modifications in the second sphere ligand scaffold in successfully tuning the reactivity of synthetic nonheme oxoiron(IV) oxidants.

## Author Contributions

**Abhishek Das**: investigation, visualization, writing – review and editing, writing – original draft, validation. **Jin Xiong**: writing – review and editing, investigation, validation, visualization. **Victor G. Young**: investigation, data curation, validation. **Yisong Guo**: writing – review and editing, investigation, supervision, funding acquisition, validation. **Marcel Swart**: writing – review and editing, supervision, conceptualization, investigation, resources, visualization, validation, funding acquisition. **Lawrence Que**: conceptualization, writing – review and editing, supervision, resources, validation, funding acquisition.

## Conflicts of Interest

The authors declare no conflicts of interest

## Supporting information



The data that support the findings of this study are available in the supplementary material of this article including experimental details for the synthesis and characterization of all compounds, full spectroscopic analysis and DFT data.
The authors have cited additional references within the Supporting Information [36, 37, 51, 53, 54, 55, 81, 83, 84, 85, 86, 88, 101, 102, 103, 104, 105, 106, 107, 108, 109, 110, 111, 112, 113, 114, 115, 116, 117, 118, 119, 120, 121, 122, 123, 124].**Supporting File**: anie74891‐sup‐0001‐SuppMat.pdf.

## Data Availability

The data that supports the findings of this study are available in the Supporting Information of this article
