# Supplementary material for: Second‐Sphere Aromatic Substitutions That Tune H‐Atom Transfer Reactivity Observed for a Series of S = 1 Nonheme FeIV=O Complexes: An Enthalpy‐Entropy Compensation Effect
Source: Angew Chem Int Ed Engl. 2026 Sep 23;65(45):e1885847. doi: 10.1002/anie.1885847 (PMC13725618; doi:10.1002/anie.1885847)
Supplement: Supplementary file 1 [file ANIE-65-e1885847-s001.pdf]

Supporting information

for

Second-Sphere Aromatic Substitutions That Tune H-Atom Transfer Reactivity Observed for a Series of *S* = 1 Fe^{IV}=O Complexes: An Enthalpy-Entropy Compensation Effect

Abhishek Das,[a] Jin Xiong,[d] Victor G. Young, Jr.,[a] Yisong Guo,[d] Marcel Swart[b,c] and Lawrence Que, Jr.*[a]*

[a] Department of Chemistry, University of Minnesota, Minneapolis, Minnesota 55455, United States.

[b] IQCC and Department of Chemistry, University of Girona, Girona 17003, Spain.

[c] ICREA, Barcelona 08010, Spain.

[d] Department of Chemistry, Carnegie Mellon University, Pittsburgh, Pennsylvania 15213, United States.

* To whom correspondence should be addressed: Marcel Swart (marcel.swart@gmail.com), or Lawrence Que, Jr. (larryque@umn.edu)

Table of Contents

Section		Page No.
**1.**	Instrumentation	**S3**
**2.**	Materials	**S3**
**3.**	Synthesis and Characterization of Ligands, Oxidants, and Iron(II) Complexes	**S4-15**
	A) General synthetic procedures for ligands reported in this study<br>B) General synthetic procedures for iron(II)-complexes reported in this study<br>C) General synthetic procedures for oxidant used in this study<br>D) ^{1}H NMR spectra of iron(II)-complexes<br>E) ^{19}F NMR spectra of iron(II)-complexes<br>F) ESI-Ms spectra of iron(II)-complexes<br>G) Mössbauer spectroscopy of iron(II) complexes<br>H) X-ray crystallographic data of the iron(II)-complexes	
**4.**	Synthesis and Characterization of Oxoiron(IV) Complexes	**S15-43**
	A) Synthesis and/or in-situ generation of oxoiron(IV) complexes<br>B) UV-vis spectroscopy of oxoiron(IV) complexes<br>C) Mössbauer spectroscopy of oxoiron(IV) complexes<br>D) Reactivity and kinetics studies of oxoiron(IV) complexes<br>E) NMR spectroscopy of oxoiron(IV) complexes<br>F) Analysis of thermodynamic parameters using Eyring plots	
**5.**	Figures Related to Discussion	**S44**
**6.**	Computational Details	**S45-48**
**7.**	References	**S49-51**

1. Instrumentation

NMR (nuclear magnetic resonance) spectra for iron(II) complexes as well as iron(IV) complexes were recorded on a Bruker 400 or 500 MHz spectrometer at temperatures stated. NMR spectra to characterize the ligands were obtained at 298 K. 8" Wilmad - LabGlass 528-PP-7-5 NMR tubes were used to collect NMR spectra for iron(IV) and iron(II) complexes. For all the variable temperature spectra, the lower temperatures were calibrated using a sealed NMR tube containing a solution of tetramethylsilane (TMS) and methanol as a standard.

UV-vis spectra and reactivity studies were recorded on a HP8453A diode array spectrometer equipped with a cryostat from Unisoku Scientific Instruments (Osaka, Japan). Mössbauer spectra were recorded with two spectrometers, using Janis Research (Wilmington, MA) SuperVaritemp dewars that allow studies in applied magnetic fields of up to 7.5 T in the temperature range from 1.5 to 200 K. A LakeShore Model 331A temperature controller was used to control the temperature in experiments. Mössbauer spectral simulations were performed using the WMOSS software package (SEE Co, Edina. Minnesota). Isomer shifts are quoted relative to α-Fe metal at 298 K. The spectra were plotted by SpinCount developed by Prof. Michael Hendrich at Carnegie Mellon University.[1]

2. Materials

All materials were bought from Sigma Aldrich, Fischer Scientific or TCI Chemicals, unless otherwise noted. The substrates cyclohexane, *tert*-butyl methyl ether, cyclooctane, toluene, ethylbenzene and toluene-d_8 used in kinetic experiments were passed through a plug of silica before use. All experiments to determine rate constants were carried out under nitrogen. Commercially available solvents like anhydrous acetonitrile, 2,2,2-trifluoroethanol (TFE) and deuterated dichloromethane, acetone and acetonitrile were used without further purification, unless otherwise noted. All oxygen- and moisture-sensitive compounds were synthesized in a nitrogen-filled glovebox.

3. Synthesis and Characterization of Ligands, Oxidants, and Iron(II) Complexes

A) <u>General synthetic procedures for ligands reported in this study.</u>

1. Ligand names and structures

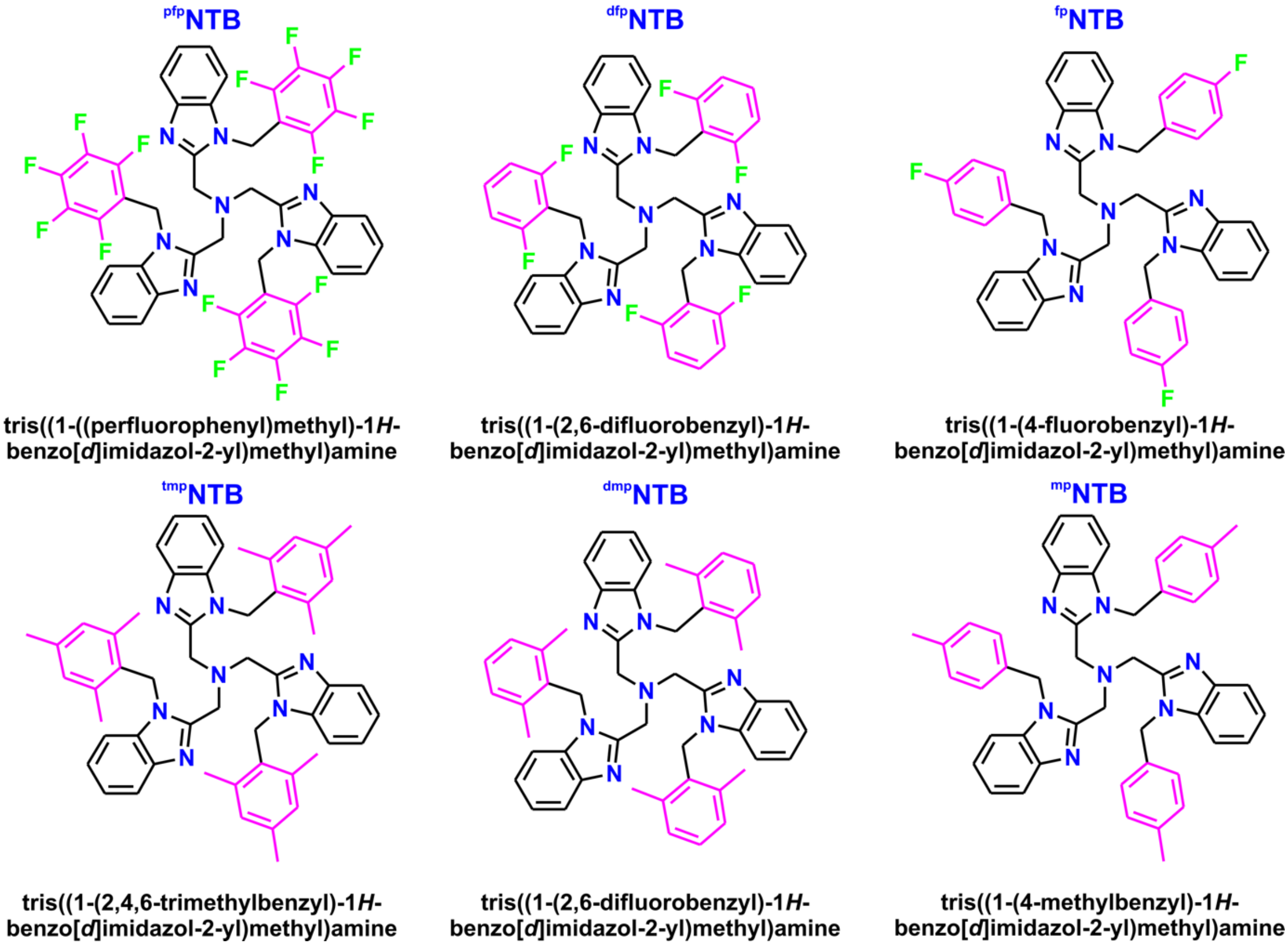

Scheme S1 Names of ligands used in this study.

NTB [tris((1*H*-benzo[*d*]imidazol-2-yl)methyl)amine] ligand has been synthesized according to the literature procedure[2] as a precursor to all the six new ligands.

2. Synthesis of ligand ^{pfp}NTB

To a 100 ml Schlenk flask protected with N_2, NTB (683 mg, 1.68 mmol), ^{t}BuOK (623 mg, 5.55 mmol) and 25 mL dry DMF were added. The resulting mixture was stirred at 50°C for 15 minutes. Then pentafluorobenzyl bromide (1.36 g, 5.21 mmol) was added to the mixture and the whole solution was stirred at 50°C for overnight maintaining the nitrogen atmosphere. Next morning, the solution was cooled down and extracted with water (50 mL) and dichloromethane (50 mL). The aqueous part was again extracted with DCM twice. The combined organic part was washed with water for three times and dried over anhydrous $MgSO_4$. Finally, the organic part was evaporated to dryness to obtain the crude product. Then it was washed with diethyl ether to get the desired ligand as white pure solid. Yield: 700 mg (44 %). ^{1}H NMR (400 MHz, $CDCl_3$, 298 K): δ (ppm) 7.53 (m, 3H), 7.15 (m, 6H), 7.07 (m, 3H), 5.48 (s, 6H), 4.49 (s, 6H). ESI-MS (+ve ion mode, methanol): m/z = 948.5 ($[M+H]^+$) and 970.4 ($[M+Na]^+$).

3. Synthesis of ligand ^{dfp}NTB

To a 100 ml Schlenk flask protected with N_2, NTB (664 mg, 1.63 mmol), ^{t}BuOK (658 mg, 5.4 mmol) and 25 mL dry DMF were added. The resulting mixture was stirred at 50°C for 15 minutes. Then 2,6-difluorobenzyl bromide (1.05 g, 5.05 mmol) was added to the mixture and the whole solution was stirred at 50°C for overnight maintaining the nitrogen atmosphere. Next morning, the solution was cooled down and extracted with water (50 mL) and dichloromethane (50 mL). The aqueous part was again extracted with DCM twice. The combined organic part was washed with water for three times and dried over anhydrous $MgSO_4$. Finally, the organic part was evaporated to dryness to obtain the crude product. Then it was washed with diethyl ether to get the desired ligand as white pure solid. Yield: 600 mg (47 %). ^{1}H NMR (400 MHz, $CDCl_3$, 298 K): δ (ppm) 7.5 (m, 3H), 7.16 (m, 6H), 7.08 (m, 6H), 6.75 (m, 6H), 5.5 (s, 6H), 4.47 (s, 6H). ESI-MS (+ve ion mode, methanol): *m/z* = 786.2 ($[M+H]^+$).

4. Synthesis of ligand ^{fp}NTB

To a 100 ml Schlenk flask protected with N_2, NTB (473 mg, 1.16 mmol), ^{t}BuOK (430 mg, 3.83 mmol) and 25 mL dry DMF were added. The resulting mixture was stirred at 50°C for 15 minutes. Then 4-fluorobenzyl bromide (679 mg, 3.59 mmol) was added to the mixture and the whole solution was stirred at 50°C for overnight maintaining the nitrogen atmosphere. Next morning, the solution was cooled down and extracted with water (50 mL) and dichloromethane (50 mL). The aqueous part was again extracted with DCM twice. The combined organic part was washed with water for three times and dried over anhydrous $MgSO_4$. Finally, the organic part was evaporated to dryness to obtain the crude product. Then it was washed with diethyl ether to get the desired ligand as white pure solid. Yield: 650 mg (77 %). ^{1}H NMR (400 MHz, CD_2Cl_2, 298 K): δ (ppm) 7.6 (m, 3H), 7.25 (m, 6H), 7.15 (m, 3H), 6.71 (t, 6H), 6.58 (t, 6H), 5.02 (s, 6H), 4.19 (s, 6H). ESI-MS (+ve ion mode, methanol): *m/z* = 732.3 ($[M+H]^+$).

5. Synthesis of ligand ^{tmp}NTB

To a 100 ml Schlenk flask protected with N_2, NTB (403 mg, 1.0 mmol), ^{t}BuOK (366 mg, 3.26 mmol) and 25 mL dry DMF were added. The resulting mixture was stirred at 50°C for 15 minutes. Then 2,4,6-trimethylbenzyl bromide (654 mg, 3.07 mmol) was added to the mixture and the whole solution was stirred at 50°C for overnight maintaining the nitrogen atmosphere. Next morning, the solution was cooled down and extracted with water (50 mL) and dichloromethane (50 mL). The aqueous part was again extracted with DCM twice. The combined organic part was washed with water for three times and dried over anhydrous $MgSO_4$. Finally, the organic part was evaporated to dryness to obtain the crude product. Then the desired ligand was obtained as white crystalline solid from acetonitrile-ether mixture. Yield: 300 mg (37 %). ^{1}H NMR (400 MHz, $CDCl_3$, 298 K): δ (ppm) 7.55 (d, 3H), 7.06 (m, 3H), 6.91 (m, 3H), 6.76 (s, 6H), 6.48 (d, 3H), 5.27 (s, 6H), 4.44 (s, 6H), 2.23 (s, 9H), 1.92 (s, 18H). ESI-MS (+ve ion mode, methanol): *m/z* = 804.8 ($[M+H]^+$) and 826.7 ($[M+Na]^+$).

6. Synthesis of ligand ^{dmp}NTB

To a 100 ml Schlenk flask protected with N_2, NTB (576 mg, 1.41 mmol), ^{t}BuOK (523 mg, 4.66 mmol) and 25 mL dry DMF were added. The resulting mixture was stirred at 50°C for 15 minutes. Then 2,6-dimethylbenzyl bromide (872 mg, 4.37 mmol) was added to the mixture and the whole solution was stirred at 50°C for overnight maintaining the nitrogen atmosphere. Next morning, the solution was cooled down and extracted with water (50 mL) and dichloromethane

(50 mL). The aqueous part was again extracted with DCM twice. The combined organic part was washed with water for three times and dried over anhydrous $MgSO_4$. Finally, the organic part was evaporated to dryness to obtain the crude product. Then it was washed with diethyl ether to get the desired ligand as white pure solid. Yield: 650 mg (60 %). ^{1}H NMR (400 MHz, $CDCl_3$, 298 K): δ (ppm) 7.54 (d, 3H), 7.08 (m, 6H), 6.9 (m, 9H), 6.45 (d, 3H), 5.33 (s, 6H), 4.44 (s, 6H), 1.96 (s, 18H). ESI-MS (+ve ion mode, methanol): *m/z* = 762.4 ($[M+H]^+$).

7. **Synthesis of ligand ^{mp}NTB**

To a 100 ml Schlenk flask protected with N_2, NTB (432 mg, 1.06 mmol), ^{t}BuOK (396 mg, 3.53 mmol) and 25 mL dry DMF were added. The resulting mixture was stirred at 50°C for 15 minutes. Then 4-methylbenzyl bromide (609 mg, 3.28 mmol) was added to the mixture and the whole solution was stirred at 50°C for overnight maintaining the nitrogen atmosphere. Next morning, the solution was cooled down and extracted with water (50 mL) and dichloromethane (50 mL). The aqueous part was again extracted with DCM twice. The combined organic part was washed with water for three times and dried over anhydrous $MgSO_4$. Finally, the organic part was evaporated to dryness to obtain the crude product. Then it was washed with diethyl ether to get the desired ligand as white pure solid. Yield: 500 mg (66 %). ^{1}H NMR (400 MHz, $CDCl_3$, 298 K): δ (ppm) 7.59 (m, 3H), 7.15-7.25 (m, 9H), 6.82 (d, 6H), 6.47 (d, 6H), 5.00 (s, 6H), 4.17 (s, 6H), 2.21 (s, 9H). ESI-MS (+ve ion mode, methanol): *m/z* = 720.4 ($[M+H]^+$).

B) <u>General synthetic procedures for iron(II)-complexes reported in this study.</u>

1. **Synthesis of iron(II) complex $[(^{pfp}NTB)Fe^{II}](OTf)_2$ (1^{pfp})**

^{pfp}NTB ligand (0.144 g, 0.15 mmol) was taken in a vial and acetonitrile (2 mL) was added to it. Then an acetonitrile solution (1 mL) of $Fe(OTf)_2{\cdot}2MeCN$ (0.067 g, 0.15 mmol) was added to the suspension and the resulting solution was stirred for overnight inside glove box. Next morning, the solution was filtered using syringe filter, and the filtrate was concentrated under vacuum. After that diethyl ether was added to the concentrated filtrate and the mixture was allowed to stand. A beige solid appeared and then the solution was decanted. The obtained solid was further washed with diethyl ether and dried to isolate the complex as beige crystalline solid. Yield: 170 mg (87 %). ESI-MS (+ve ion mode in CH_3CN): *m/z* = 1152.2 for $[Fe(^{pfp}NTB)(OTf)]^+$ and 501.6 for $[Fe(^{pfp}NTB)]^{2+}$.

2. **Synthesis of iron(II) complex $[(^{dfp}NTB)Fe^{II}](OTf)_2$ (1^{dfp})**

^{dfp}NTB ligand (0.100 g, 0.13 mmol) was taken in a vial and acetonitrile (2 mL) was added to it. Then an acetonitrile solution (1 mL) of $Fe(OTf)_2{\cdot}2MeCN$ (0.056 g, 0.13 mmol) was added to the suspension and the resulting solution was stirred for overnight inside glove box. Next morning, the solution was filtered using syringe filter, and the filtrate was concentrated under vacuum. After that diethyl ether was added to the concentrated filtrate and the mixture was allowed to stand. A white solid appeared and then the solution was decanted. The obtained solid was further washed with diethyl ether and dried to isolate the complex as white crystalline solid. Yield: 130 mg (90 %). ESI-MS (+ve ion mode in CH_3CN): *m/z* = 990.3 for $[Fe(^{dfp}NTB)(OTf)]^+$ and 420.7 for $[Fe(^{dfp}NTB)]^{2+}$. X-ray quality single crystals of complex were grown by vapour diffusion of the diethyl ether to a saturated acetonitrile solution of complex at room temperature under inert condition.

3. Synthesis of iron(II) complex [(^{fp}NTB)Fe^{II}](OTf)$_2$ (1^{fp})

^{fp}NTB ligand (0.110 g, 0.15 mmol) was taken in a vial and acetonitrile (2 mL) was added to it. Then an acetonitrile solution (1 mL) of Fe(OTf)$_2$·2MeCN (0.066 g, 0.15 mmol) was added to the suspension and the resulting solution was stirred for overnight inside glove box. Next morning, the solution was filtered using syringe filter, and the filtrate was concentrated under vacuum. After that diethyl ether was added to the concentrated filtrate and the mixture was allowed to stand. A white solid appeared and then the solution was decanted. The obtained solid was further washed with diethyl ether and dried to isolate the complex as white crystalline solid. Yield: 150 mg (89 %). ESI-MS (+ve ion mode in CH$_3$CN): *m/z* = 936.2 for [Fe(^{fp}NTB)(OTf)]$^+$ and 393.6 for [Fe(^{fp}NTB)]$^{2+}$.

4. Synthesis of iron(II) complex [(^{tmp}NTB)Fe^{II}](OTf)$_2$ (1^{tmp})

^{tmp}NTB ligand (0.110 g, 0.137 mmol) was taken in a vial and acetonitrile (2 mL) was added to it. Then an acetonitrile solution (1 mL) of Fe(OTf)$_2$·2MeCN (0.060 g, 0.137 mmol) was added to the suspension and the resulting solution was stirred for overnight inside glove box. Next morning, the solution was filtered using syringe filter, and the filtrate was concentrated under vacuum. After that diethyl ether was added to the concentrated filtrate and the mixture was allowed to stand. A light yellow solid appeared and then the solution was decanted. The obtained solid was further washed with diethyl ether and dried to isolate the complex as light yellow crystalline solid. Yield: 130 mg (82 %). ESI-MS (+ve ion mode in CH$_3$CN): *m/z* = 1008.7 for [Fe(^{tmp}NTB)(OTf)]$^+$, 450.4 for [Fe(^{tmp}NTB)(MeCN)]$^{2+}$ and 429.8 for [Fe(^{tmp}NTB)]$^{2+}$.

5. Synthesis of iron(II) complex [(^{dmp}NTB)Fe^{II}](OTf)$_2$ (1^{dmp})

^{dmp}NTB ligand (0.124 g, 0.16 mmol) was taken in a vial and acetonitrile (2 mL) was added to it. Then an acetonitrile solution (1 mL) of Fe(OTf)$_2$·2MeCN (0.071 g, 0.16 mmol) was added to the suspension and the resulting solution was stirred for overnight inside glove box. Next morning, the solution was filtered using syringe filter, and the filtrate was concentrated under vacuum. After that diethyl ether was added to the concentrated filtrate and the mixture was allowed to stand. A yellow-coloured solid appeared and then the solution was decanted. The obtained solid was further washed with diethyl ether and dried to isolate the complex as light-yellow coloured crystalline solid. Yield: 140 mg (78 %). ESI-MS (+ve ion mode in CH$_3$CN): *m/z* = 408.8 for [Fe(^{dmp}NTB)]$^{2+}$.

6. Synthesis of iron(II) complex [(^{mp}NTB)Fe^{II}](OTf)$_2$ (1^{mp})

^{mp}NTB ligand (0.109 g, 0.152 mmol) was taken in a vial and acetonitrile (2 mL) was added to it. Then an acetonitrile solution (1 mL) of Fe(OTf)$_2$·2MeCN (0.066 g, 0.152 mmol) was added to the suspension and the resulting solution was stirred for overnight inside glove box. Next morning, the solution was filtered using syringe filter, and the filtrate was concentrated under vacuum. After that diethyl ether was added to the concentrated filtrate and the mixture was allowed to stand. An off-white solid appeared and then the solution was decanted. The obtained solid was further washed with diethyl ether and dried to isolate the complex as white crystalline solid. Yield: 140 mg (86 %). ESI-MS (+ve ion mode in CH$_3$CN): *m/z* = 924.2 for [Fe(^{mp}NTB)(OTf)]$^+$ and 387.6 for [Fe(^{mp}NTB)]$^{2+}$.

All the $^{57}Fe^{II}$ -labelled complexes were synthesized using the same synthetic procedures as above, except that $^{57}Fe^{II}(CH_3CN)_2(OTf)_2$ was used on a 10-20 mg scale as the iron(II) triflate starting material. Details for the preparation of oxoiron(IV) solutions for Mössbauer spectroscopy are detailed in the Mössbauer section.

C) <u>General synthetic procedures for oxidant used in this study.</u>

Synthesis of 2-(tert-butylsulfonyl)iodosylbenzene [2-(^{t}BuSO$_2$)C$_6$H$_4$IO)/*s*-PhIO]

2-(^{t}BuSO$_2$)C$_6$H$_4$IO has been prepared following the previously described procedures.[3-5]

Table S1 ^{1}H and ^{19}F NMR assignment table for the iron(II)-complexes at 298 K in CD_3CN.

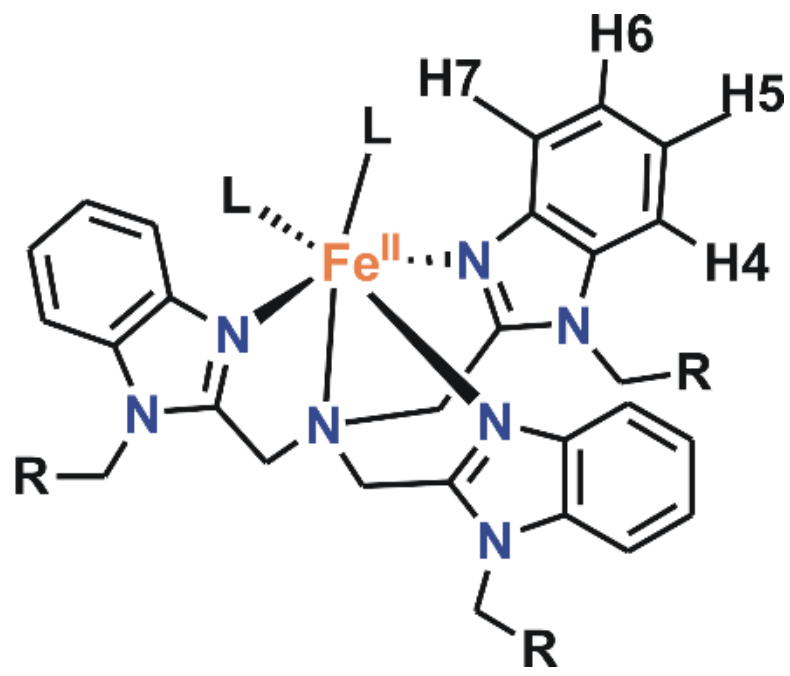

Complex	H4 (ppm)	H5/H6 (ppm)	N-CH$_2$-R (ppm)	$^{19}F_{OTf}$ (ppm)	$^{19}F_{Ligand}$ (ppm)
**3**	37.3	-0.6, 0.5	22.8	-	N.A.
**1^{pfp}**	27.1	4.2, 2.2	14.0	-75.6	-135.1, -153.9, 162.8
**1^{dfp}**	26.8	4.4, 1.1	14.5	-77.3	-107.4
**1^{fp}**	26.3	4.6, 1.7	15.1	-76.5	-115.1
**1^{tmp}**	26.0	4.3, 1.7	21.4	-76.2	N.A.
**1^{dmp}**	26.1	4.2, 1.8	21.0	-76.6	N.A.
**1^{mp}**	25.9	4.3, 1.6	14.7	-77.3	N.A.

In Table S1, the proton signals are assigned based on the reported[6] ^{1}H NMR of **3** ($[Fe^{II}(Me_3NTB)(L)]^{2+}$ complex).

Upon comparing the proton NMR spectra of iron(II) complexes for the series of molecules with different aromatic substituents, significant shifts in the proton signal patterns can be observed only for benzylic (N-CH$_2$-R) protons with the change in aromatic substituents, showing the benzylic position to be the most sensitive to the aromatic substituents. Furthermore, a shift in the benzimidazole H4 proton signal is also observed when comparing the results to those of complex **3**, but no such change occurs across the series. This H4 is the proton is closest to the aromatic substituents and therefore most affected by the magnetic field of the π-cloud of the (substituted) phenyl rings.

D) ^{1}H NMR spectra of iron(II)-complexes.

Paramagnetically shifted proton resonances observed in the ^{1}H NMR spectra of all the iron(II) complexes in CD_3CN solution reflect the high-spin nature of the iron(II) center. A broad peak at roughly within 80-90 ppm is seen in the proton NMR spectra of the complexes in CD_3CN and assigned easily to coordinated MeCN molecules, indicating that all of the complexes in solutions have bound acetonitrile solvent molecules in their coordination sphere.

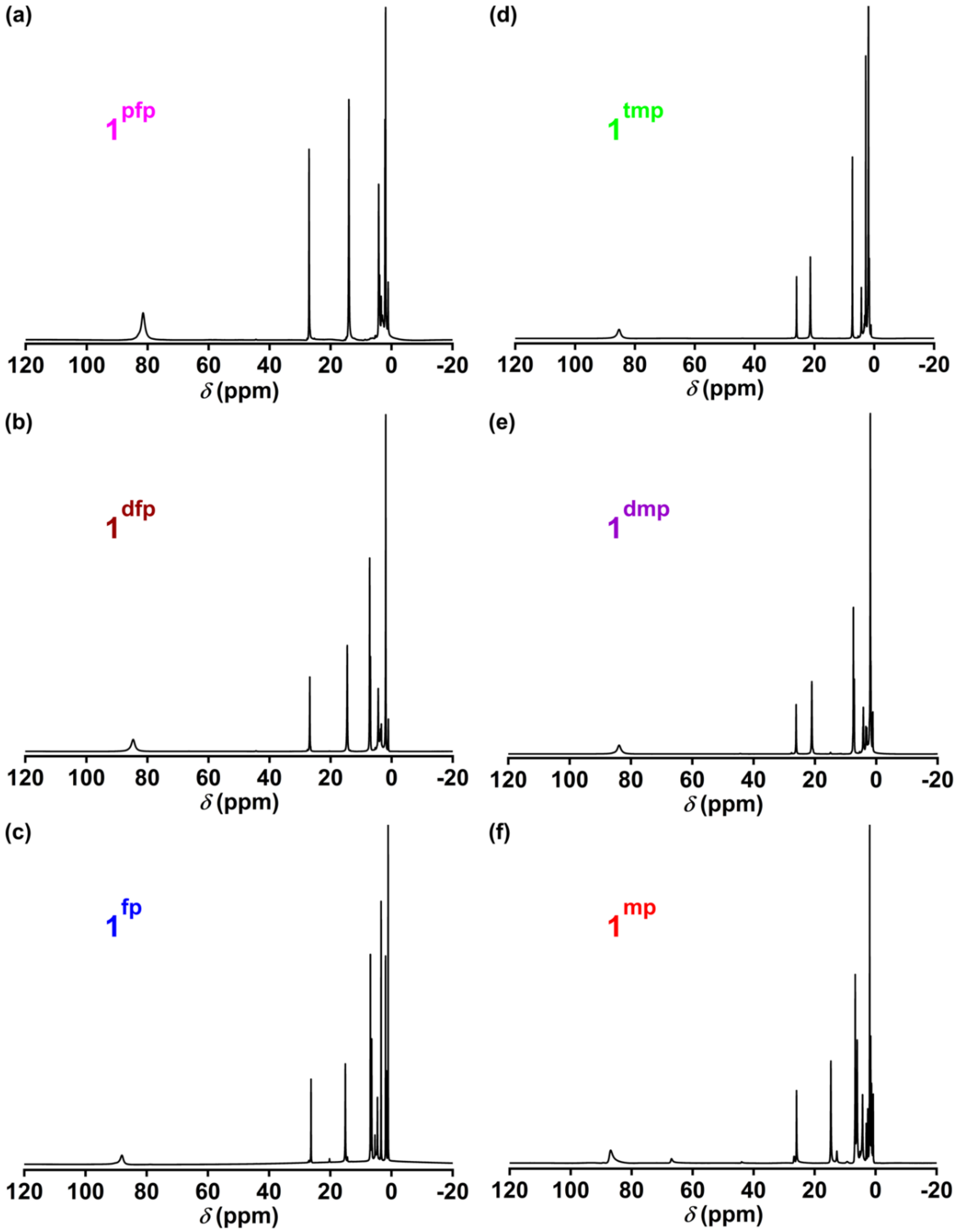

Figure S1 ^{1}H NMR spectrum (400 MHz, CD_3CN, 298 K) of complex (a) $\mathbf{1^{pfp}}$, (b) $\mathbf{1^{dfp}}$, (c) $\mathbf{1^{fp}}$, (d) $\mathbf{1^{tmp}}$, (e) $\mathbf{1^{dmp}}$ and (f) $\mathbf{1^{mp}}$.

E) ^{19}F NMR spectra of iron(II)-complexes.

A sharp peak at around -77 ppm in their ^{19}F NMR spectra is observed, which likely originates from the two non-coordinated triflate counter ions. Besides the triflate counter-anion peaks, other distinct signals are detected in the upfield regions of the ^{19}F NMR spectra of **1**$^{\mathbf{fp}}$, **1**$^{\mathbf{dfp}}$, and **1**$^{\mathbf{pfp}}$, originating from the fluoro-atoms of fluorine-containing aromatic substitutions.

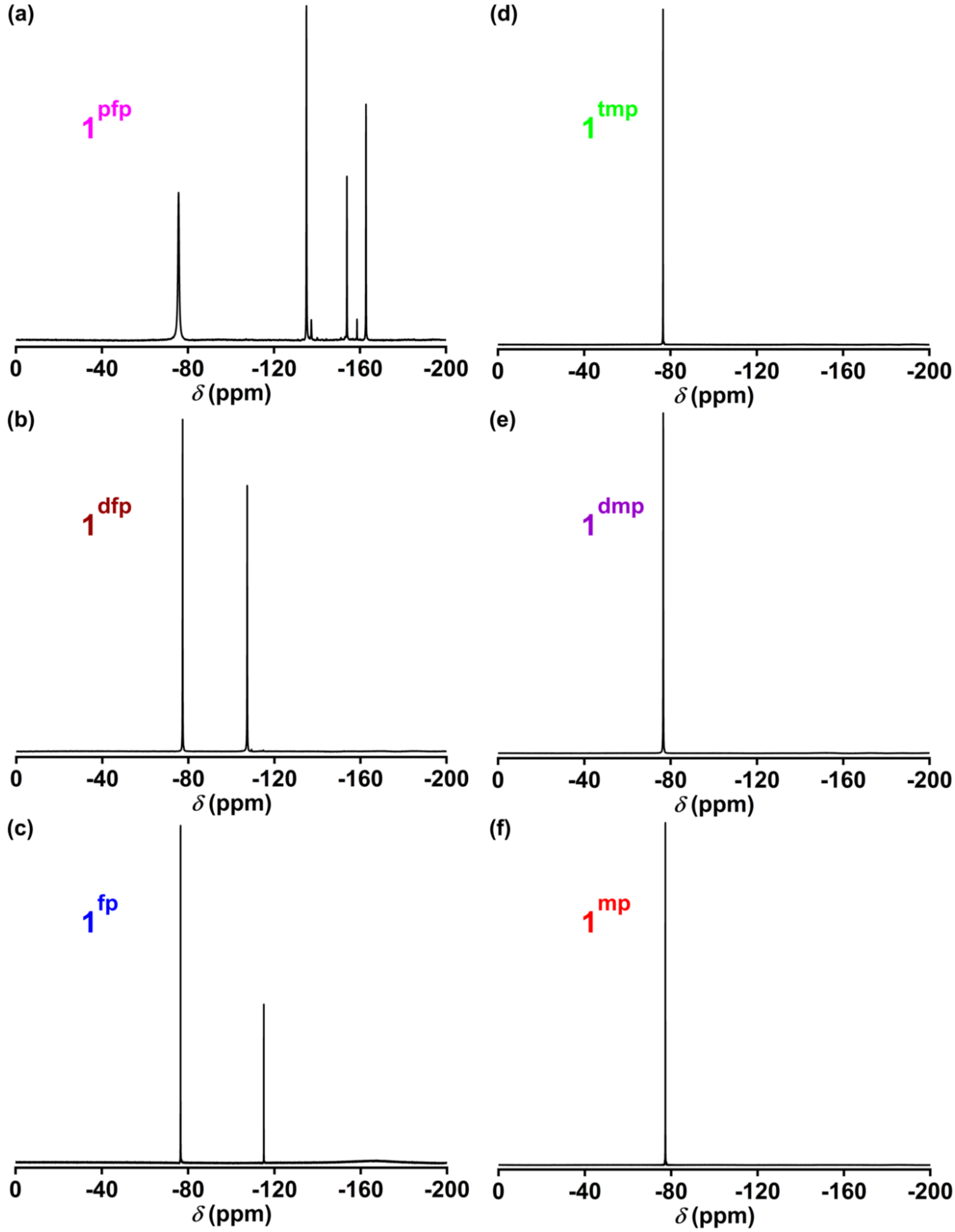

Figure S2 ^{19}F NMR spectrum (400 MHz, CD_3CN, 298 K) of complex (a) **1**$^{\mathbf{pfp}}$, (b) **1**$^{\mathbf{dfp}}$, (c) **1**$^{\mathbf{fp}}$, (d) **1**$^{\mathbf{tmp}}$, (e) **1**$^{\mathbf{dmp}}$ and (f) **1**$^{\mathbf{mp}}$.

F) <u>ESI-Ms spectra of iron(II)-complexes.</u>

The ESI-mass spectra (positive ion mode) of iron(II)-complexes in MeCN reveals ion peaks in agreement with isotope distribution patterns that are compatible with $[(L)Fe]^{2+}$ and $[(L)Fe(OTf)]^{+}$ ions.

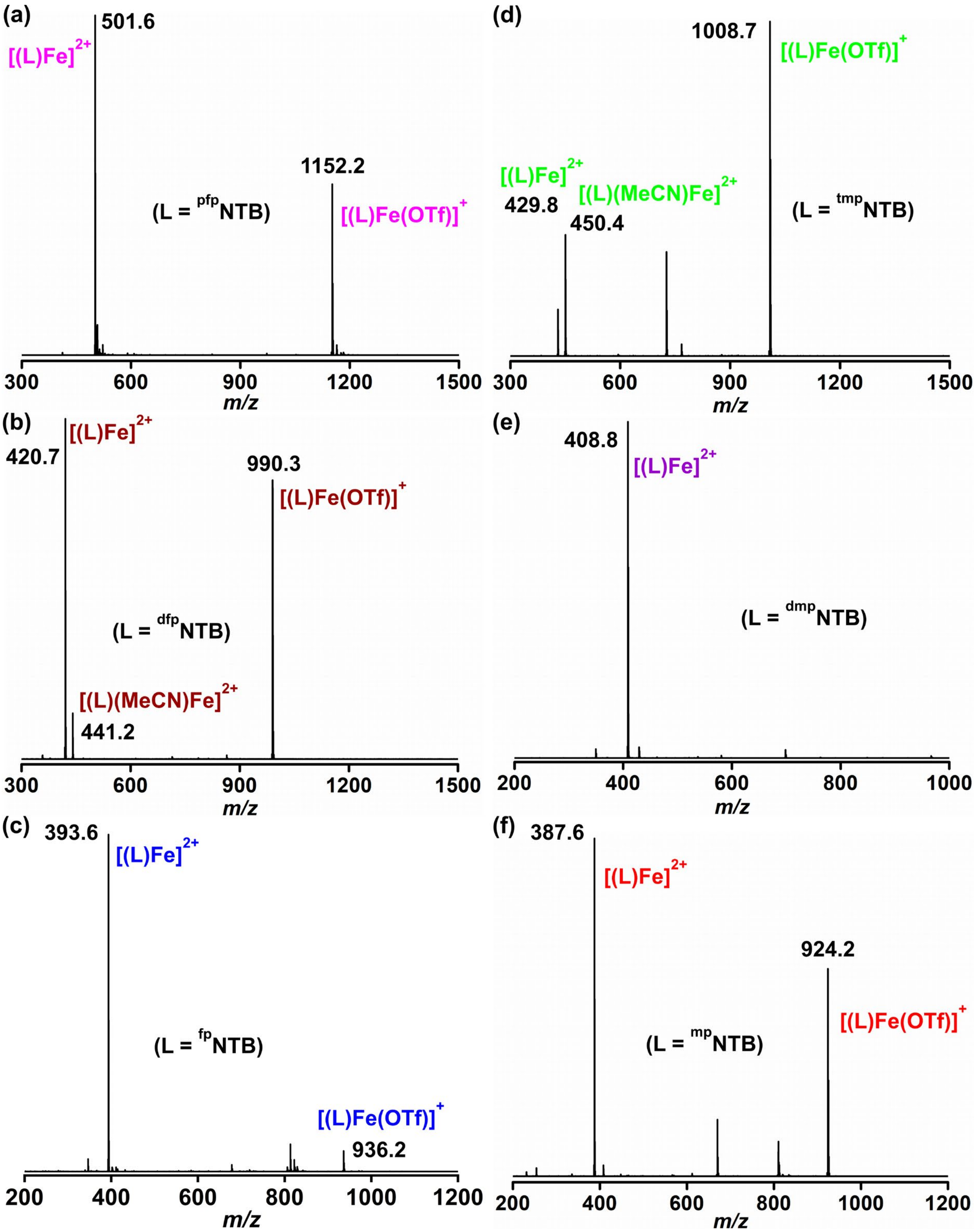

Figure S3 ESI-mass spectrum (positive ion mode in acetonitrile) of complex (a) $\mathbf{1^{pfp}}$, (b) $\mathbf{1^{dfp}}$, (c) $\mathbf{1^{fp}}$, (d) $\mathbf{1^{tmp}}$, (e) $\mathbf{1^{dmp}}$ and (f) $\mathbf{1^{mp}}$.

G) <u>Mössbauer spectroscopy of iron(II) complexes.</u>

A Two-sites model is employed for a better simulation of the line shape, which could come from the variation of the solvent ligand in the samples.

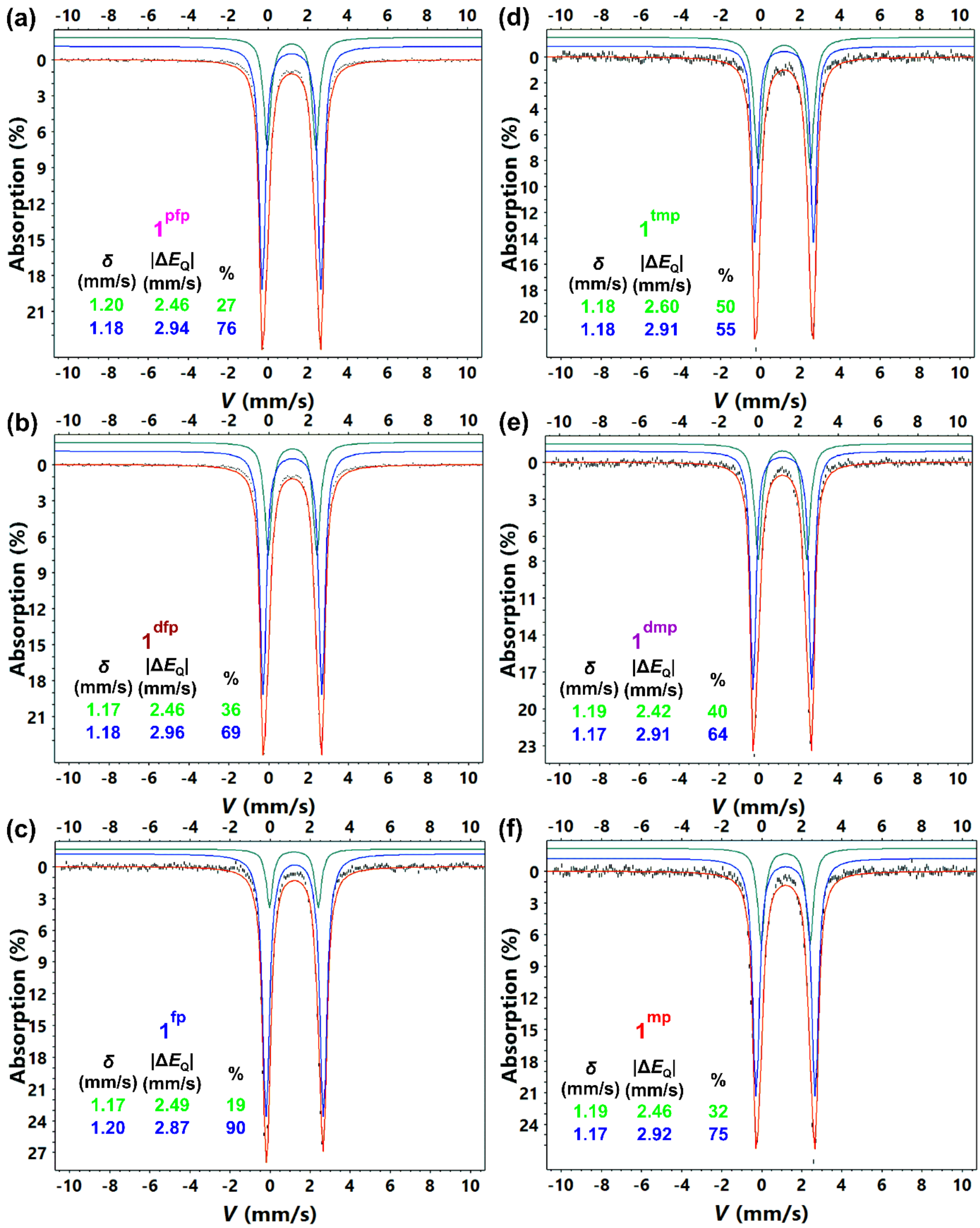

Figure S4 Mössbauer spectrum of ^{57}Fe enriched sample of (a) $\mathbf{1}^{\mathbf{pfp}}$, (b) $\mathbf{1}^{\mathbf{dfp}}$, (c) $\mathbf{1}^{\mathbf{fp}}$, (d) $\mathbf{1}^{\mathbf{tmp}}$, (e) $\mathbf{1}^{\mathbf{dmp}}$ and (f) $\mathbf{1}^{\mathbf{mp}}$ (in acetonitrile) recorded at 4.2 K.

H) X-ray crystallographic data of the iron(II)-complexes.

Data collection

A crystal (approximate dimensions 0.140 x 0.140 x 0.035 mm^3) was placed onto the tip of a 200 µm MiTeGen Dual-Thickness Microloop and mounted on a Bruker PHOTON-III CPAD diffractometer for a data collection at 130(2) K.[7] A preliminary set of cell constants was calculated from reflections harvested from three sets of frames. These initial sets of frames were oriented such that orthogonal wedges of reciprocal space were surveyed. This produced an initial orientation matrix determined from 1639 reflections. The data collection was carried out using MoKα radiation (parabolic mirrors) with a frame time of 20 seconds and a detector distance of 5.0 cm. A strategy program was used to assure complete coverage of all unique data to a resolution of 0.75 Å. All major sections of frames were collected with 1.30° steps in ω or ϕ at different detector positions in 2θ. The intensity data were corrected for absorption and decay (SADABS).[8] Final cell constants were calculated from 2903 strong reflections from the actual data collection after integration (SAINT).[9] Please refer to Table S2 for additional crystal and refinement information.

Structure solution and refinement

The structure was solved using SHELXT-2018/2 (Sheldrick, 2018)[10-11] and refined using SHELXL-2019/2 (Sheldrick, 2019).[10-11] The space group *P*-1 was determined based on systematic absences and intensity statistics. A direct-methods solution was calculated which provided most non-hydrogen atoms from the E-map. Full-matrix least squares / difference Fourier cycles were performed which located the remaining non-hydrogen atoms. All non-hydrogen atoms were refined with anisotropic displacement parameters. All hydrogen atoms were placed in ideal positions and refined as riding atoms with relative isotropic displacement parameters. The final full matrix least squares refinement converged to the $R1 = 0.0388$ and $wR2 = 0.0983$ (F^2, obs. data).

Result Interpretation

A comparison of structural properties (Table S4, SI) of all five structures of NTB based iron(II)-complexes shows very similar Fe-N_{Axial} bond distances in all cases, but for the $\mathbf{1}^{\mathbf{dfp}}$ complex, which exhibits larger average Fe-$N_{Equatorial}$ bond distances than the other four previously reported complexes. The deformed octahedron of $\mathbf{1}^{\mathbf{dfp}}$ has three benimidazole nitrogens (N2, N4 and N6), and one nitrogen from one acetonitrile molecule (N8) that occupy its equatorial sites, while the axial positions are occupied by the amine nitrogen of the tripodal ligand (N1) and the nitrogen atom from a second acetonitrile molecule, giving rise to the largest angle (∠N1-Fe1-N9 = 174.45 (5)°) of the distorted octahedral structure (Table S3, SI). As can be seen from the structural comparison, the substituted benzimidazole ligand in this family of iron(II)-complexes supported by substituted NTB ligands favor an octahedral geometry over trigonal bipyramidal geometry when aromatic substitution is incorporated.

Table S2 Crystallographic data for complex **1^dfp^**.

**Parameters**	**Complex 1^{dfp}**
Empirical formula	$C_{53} H_{42.83} F_{12} Fe N_{10} O_{6.41} S_2$
Formula weight	1270.38
Temperature/K	130(2)
Crystal system	Triclinic
Space group	*P*-1
a (Å)	12.4458(11)
b (Å)	13.4563(12)
c (Å)	17.3249(12)
α (deg.)	85.404(3)
β (deg.)	71.016(3)
γ (deg.)	88.079(3)
Volume ($Å^3$)	2734.8(4)
Z	2
$D_{calcd.}$ (mg/m^3)	1.543
μ Mo-K$_\alpha$ (mm^{-1})	0.454
F(000)	1296
Crystal size/mm^3	0.140 x 0.140 x 0.035
Radiation	MoKα (λ = 0.71073)
2θ range for data collection/°	2.034 to 28.325
Index ranges	$-16 \leq h \leq 16$, $-17 \leq k \leq 17$, $-21 \leq l \leq 23$
Reflections collected	91056
Independent reflections	13591 [R_{int} = 0.0331]
Data/restraints/parameters	13591 / 27 / 798
Goodness-of-fit on F^2	1.052
Final R indexes [I>=2σ (I)]	R_1 = 0.0388, wR_2 = 0.0983
Final R indexes [all data]	R_1 = 0.0496, wR_2 = 0.1086
Largest diff. peak/hole / e Å^{-3}	1.012 / -0.548

Table S3 Selected bond distances (Å) and angles (°) for **1**$^{\mathbf{dfp}}$.

Bond distances (Å) /angles (°)	Complex **2**$^{\mathbf{dfp}}$
Fe1-N1	2.3370(13)
Fe1-N2	2.1178(13)
Fe1-N4	2.1353(14)
Fe1-N6	2.1390(14)
Fe1-N8	2.2621(16)
Fe1-N9	2.1121(15)
N9-Fe1-N2	105.42(6)
N9-Fe1-N4	103.88(6)
N2-Fe1-N4	147.82(5)
N9-Fe1-N6	97.71(6)
N2-Fe1-N6	87.47(5)
N4-Fe1-N6	101.54(5)
N9-Fe1-N8	86.16(6)
N2-Fe1-N8	86.61(5)
N4-Fe1-N8	82.35(6)
N6-Fe1-N8	173.62(5)
N9-Fe1-N1	174.45(5)
N2-Fe1-N1	76.96(5)
N4-Fe1-N1	75.10(5)
N6-Fe1-N1	77.27(5)
N8-Fe1-N1	99.05(5)

Table S4 Comparison of structural parameters of **1**$^{\mathbf{dfp}}$, **2**$^{\mathbf{\mathit{c}\text{-}propyl}}$,**2**$^{\mathbf{\mathit{c}\text{-}hexyl}}$, **2**$^{\mathbf{oxanyl}}$ and **3**.

Parameters	**3**	**2**$^{\mathbf{\mathit{c}\text{-}propyl}}$	**2**$^{\mathbf{\mathit{c}\text{-}hexyl}}$	**2**$^{\mathbf{oxanyl}}$	**1**$^{\mathbf{dfp}}$
Fe-$N_{Equatorial}$	2.0647 (3) Å	2.0702 (17)	2.0706 (16)	2.0787 (10)	2.1307(14)
Fe-N_{Axial}	2.2990 (3) Å	2.3469 (18)	2.3090 (15)	2.3140 (10)	2.3370(13)
Geometry	TBP	TBP	TBP	TBP	Oh
5th / 6th Ligand	MeCN	Triflate	Perchlorate	Triflate	MeCN

4. Synthesis and Characterization of Oxoiron(IV) Complexes

A) <u>Synthesis and/or in-situ generation of oxoiron(IV) complexes.</u>

All oxoiron(IV) complexes supported by tripodal ligands in this study were generated at given temperatures by treatment of solutions of their iron(II) precursors of given concentration in appropriate solvents with 1-2 equivalents of oxidant ArIO. These solutions were used for reactivity studies or to obtain NMR, UV-visible or Mössbauer spectra. Details are given in the relevant section for sample preparation and each spectroscopic measurement.

B) UV-vis spectroscopy of oxoiron(IV) complexes.

All samples were generated in 1 cm quartz cells at appropriate temperatures using 1 mL of 1-mM iron(II) solutions in acetonitrile, to which 2 equivalents of ArIO dissolved in either 2,2,2-trifluoroethanol or dichloromethane-d_2 was added. Their generation was followed using UV-vis spectroscopy at given temperatures using the cryostat-equipped UV-visible spectrophotometer, to stabilize and lower the temperature using liquid nitrogen from a dewar.

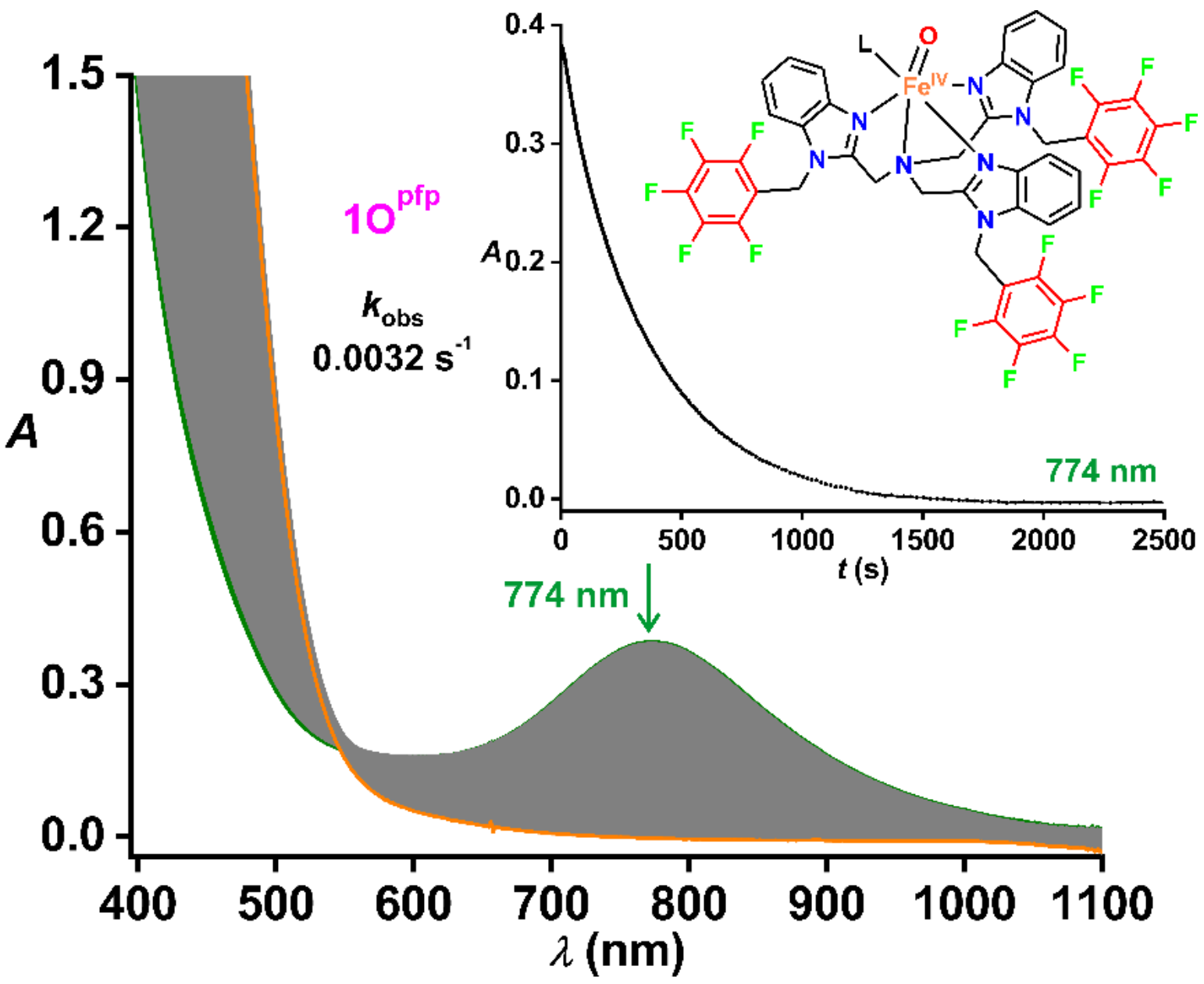

Figure S5 Decay of $\mathbf{1O^{pfp}}$, generated from $\mathbf{1^{pfp}}$ (1.0 mM) with ^{S}PhIO in CH_3CN at 233 K. Inset: time trace for the band at 774 nm.

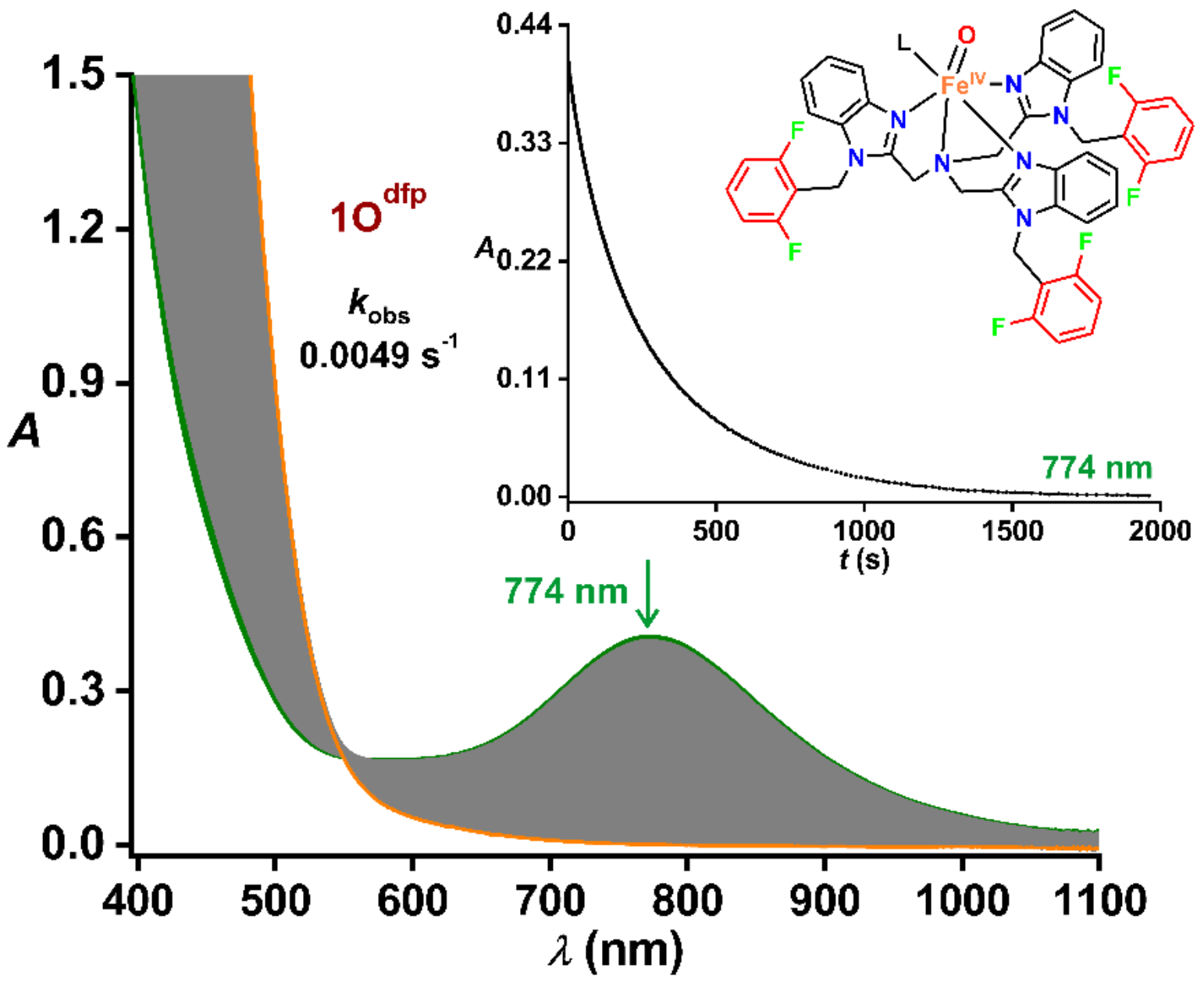

Figure S6 Decay of $\mathbf{1O^{dfp}}$, generated from $\mathbf{1^{dfp}}$ (1.0 mM) with ^{S}PhIO in CH_3CN at 233 K. Inset: time trace for the band at 774 nm.

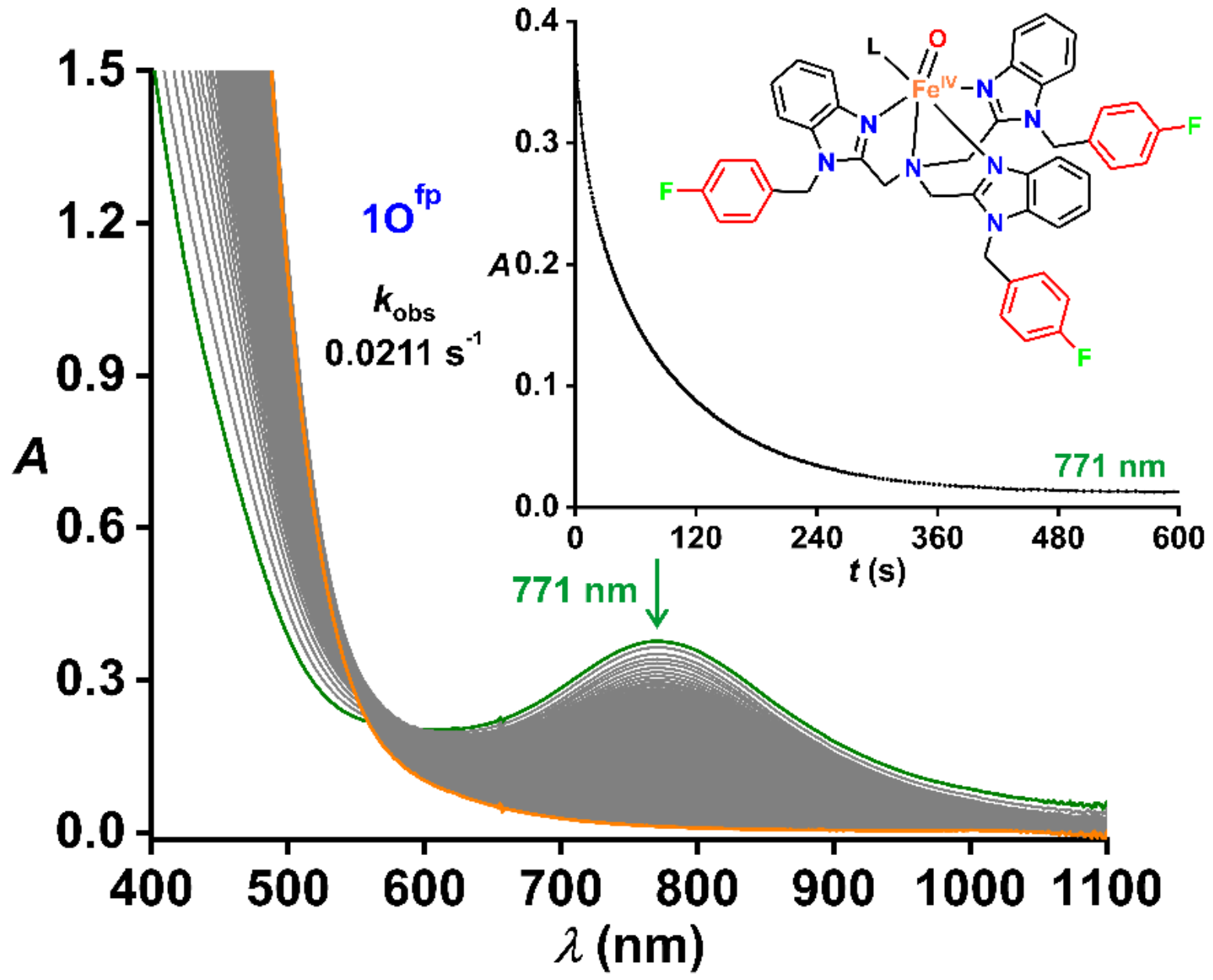

Figure S7 Decay of $\mathbf{1O^{fp}}$, generated from $\mathbf{1^{fp}}$ (1.0 mM) with ^{S}PhIO in CH_3CN at 233 K. Inset: time trace for the band at 771 nm.

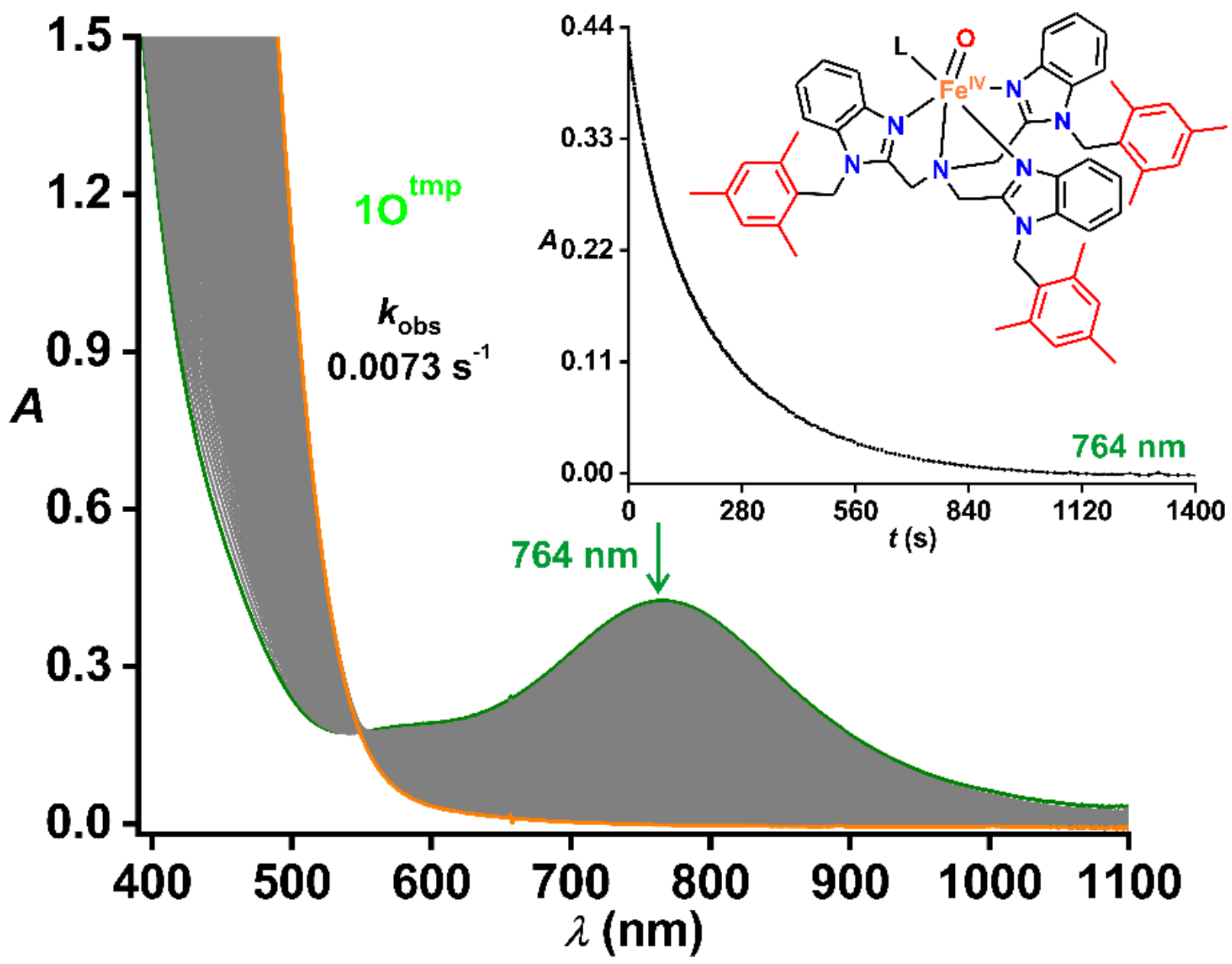

Figure S8 Decay of $\mathbf{1O^{tmp}}$, generated from $\mathbf{1^{tmp}}$ (1.0 mM) with ^{S}PhIO in CH_3CN at 233 K. Inset: time trace for the band at 764 nm.

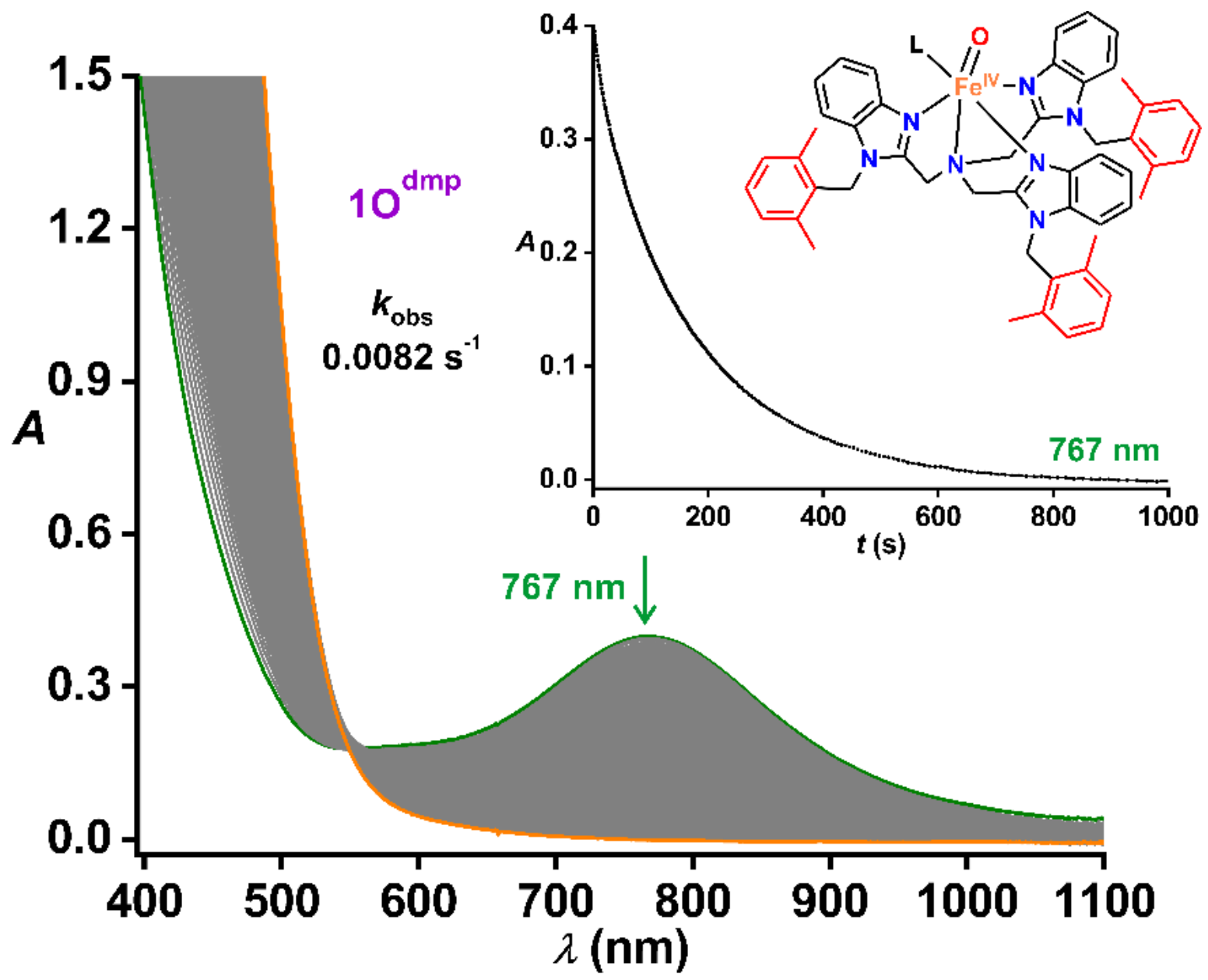

Figure S9 Decay of **$1O^{dmp}$**, generated from **1^{dmp}** (1.0 mM) with ^{S}PhIO in CH_3CN at 233 K. Inset: time trace for the band at 767 nm.

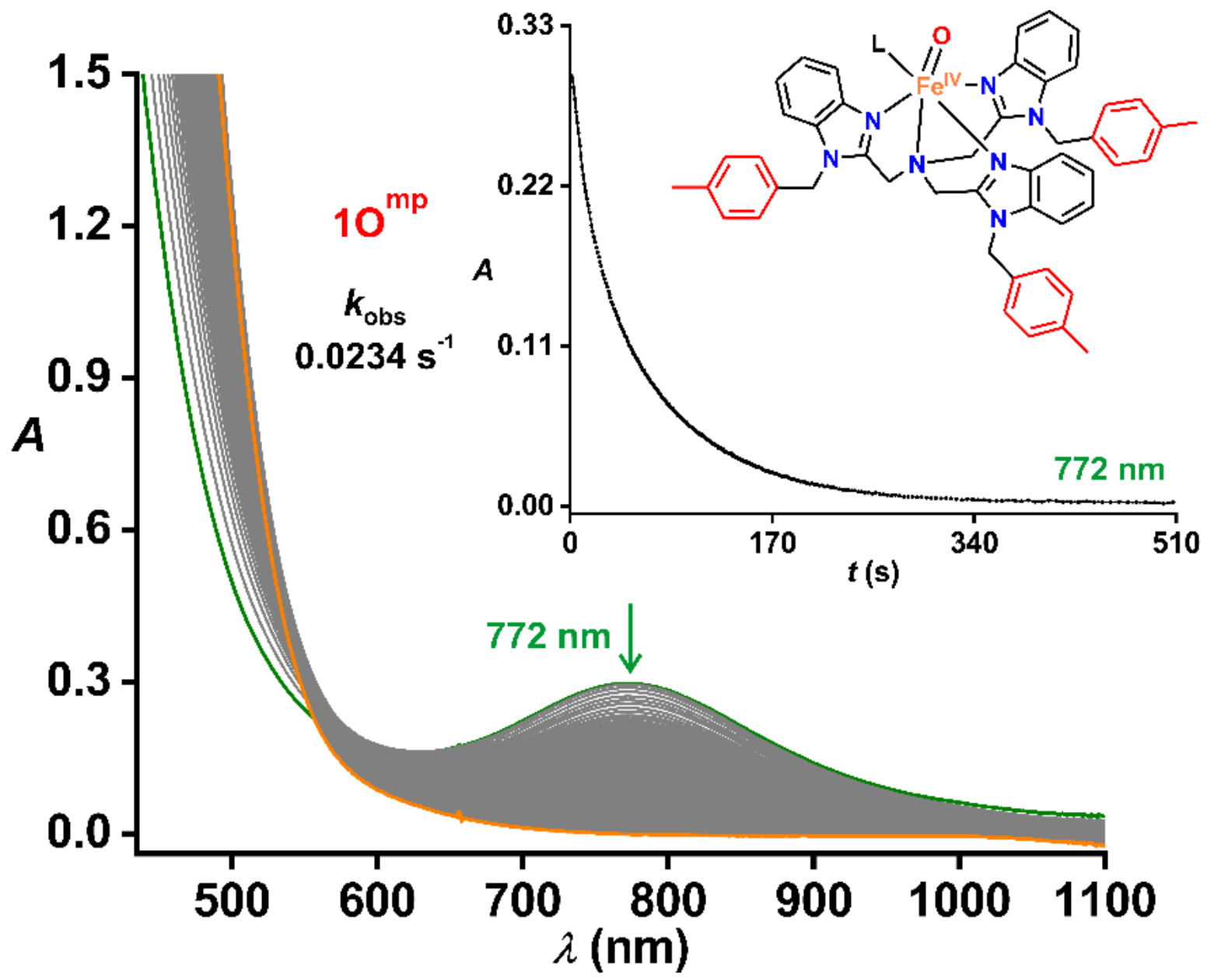

Figure S10 Decay of **$1O^{mp}$**, generated from **1^{mp}** (1.0 mM) with ^{S}PhIO in CH_3CN at 233 K. Inset: time trace for the band at 772 nm.

C) Mössbauer spectroscopy of oxoiron(IV) complexes.

1-mM / 2-mM acetone-acetonitrile (8:2) solutions of labelled (^{57}Fe) iron(II) complexes were precooled in a quartz cell at 193 K. To this stirred solution, 2 equivalents of the oxidant *s*-PhIO in DCM-d_2 were added. The formation of the oxoiron(IV) complex was instantaneous in each case. Each sample was then transferred using cryo-cooled 9” glass pipettes into a precooled Mössbauer cup suspended on top of liquid nitrogen in a dewar.

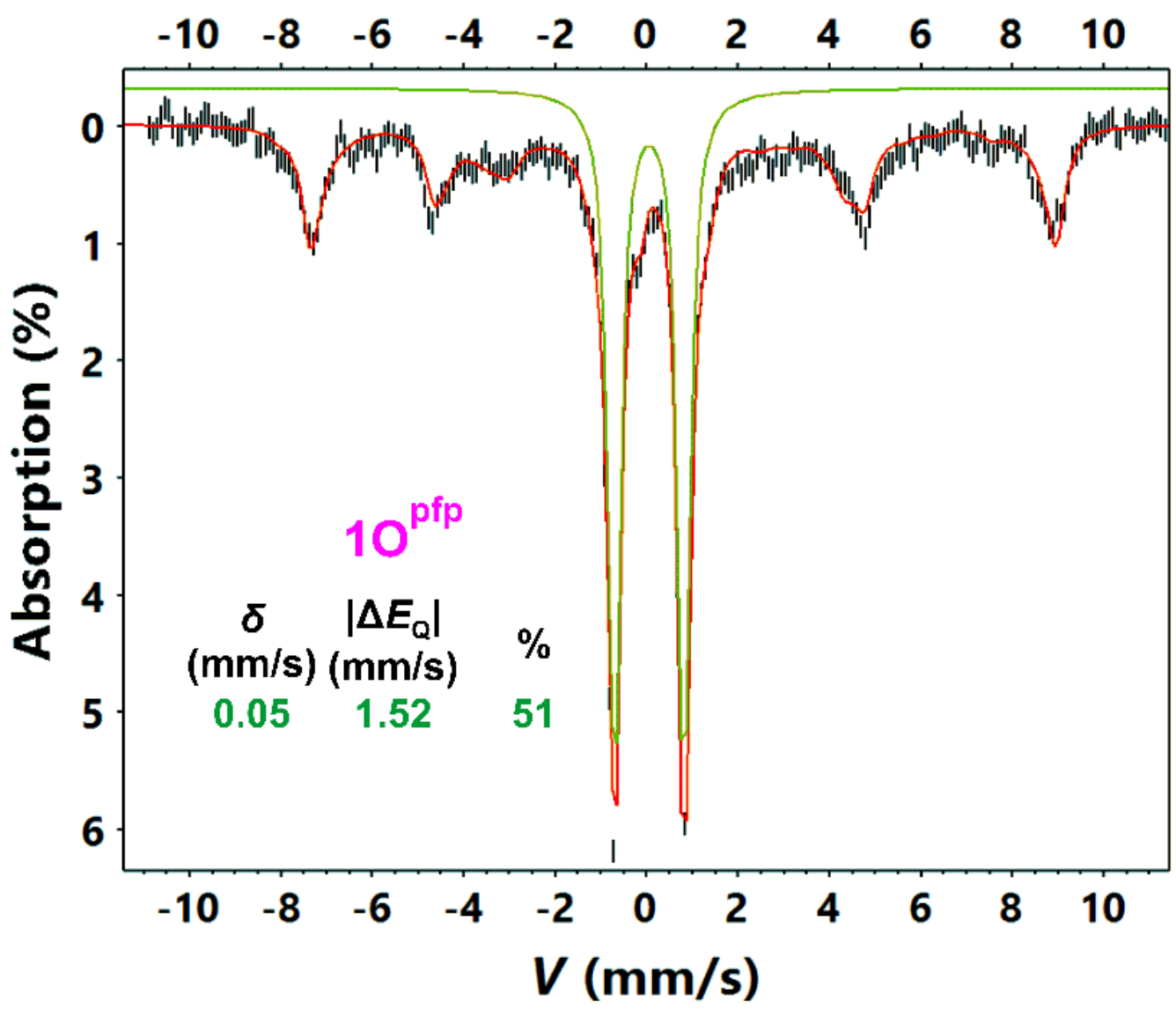

Figure S11 ^{57}Fe Mössbauer spectrum of ^{57}Fe enriched sample of $\mathbf{1O^{pfp}}$ recorded at 4.2 K under an external filed of 0.1 T parallel to the radiation. Black bars represent experimental error, red line indicates overall simulations, green line shows the Fe^{IV} component. In order to obtain better estimates of Fe^{IV}, one high-spin Fe^{III} species and one diamagnetic Fe^{III} species (e.g. ferric dimer) were included into the simulation to cover the area, the parameters of which are tentative: $S = 5/2$, $D = -1.2$ cm^{-1}, $E/D = 0.31$, $\delta = 0.43$ mm/s, $\Delta E_Q = 1.38$ mm/s, $\eta = 0.1$, $A/g_n\beta_n = [-20.7, -21.1, -20.0]$ T, Area = 45%; $S = 0$, $\delta = 0.42$ mm/s, $|\Delta E_Q| = 1.10$ mm/s, Area = 2%.

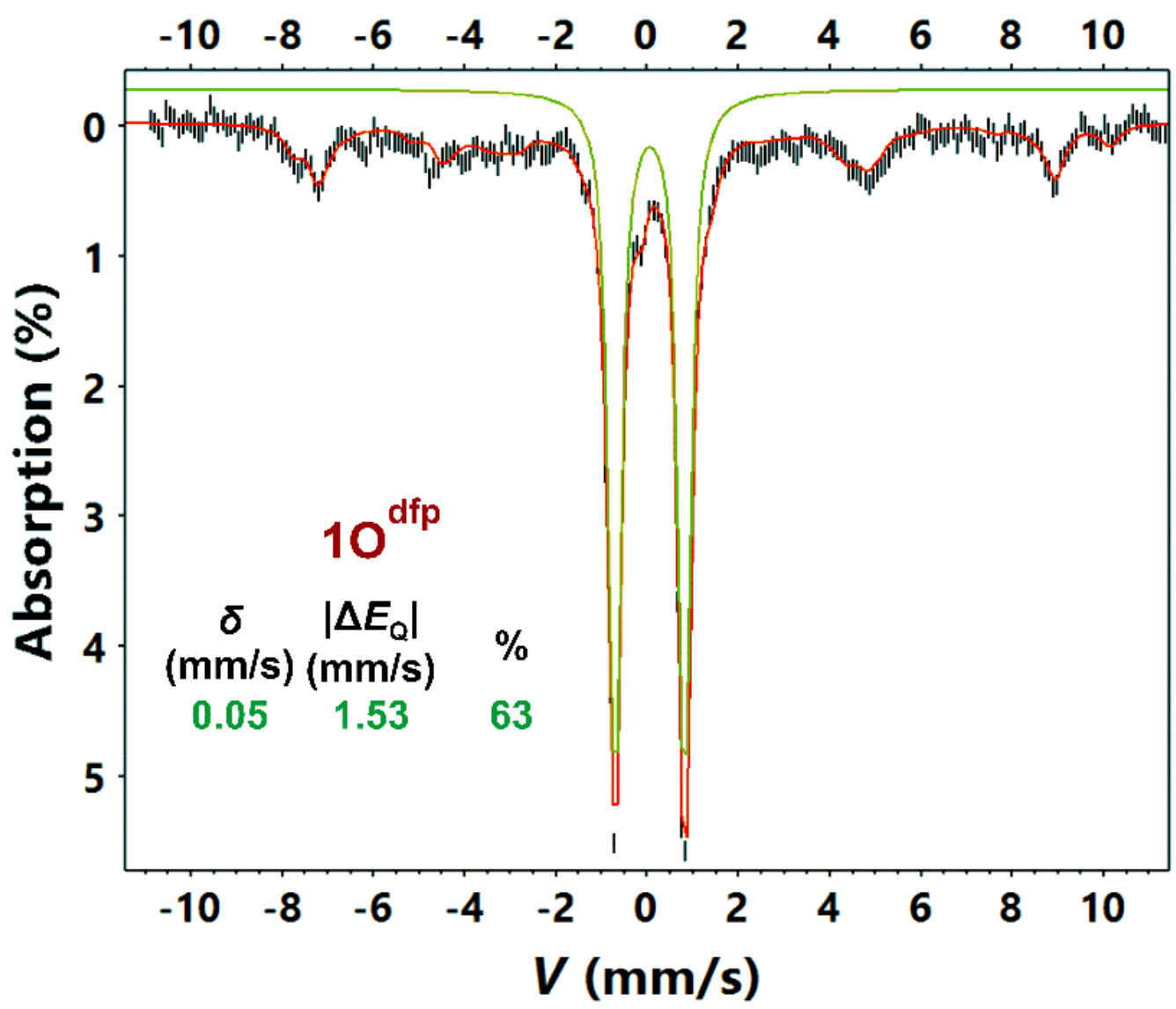

Figure S12 ^{57}Fe Mössbauer spectrum of ^{57}Fe enriched sample of $\mathbf{1O^{dfp}}$ recorded at 4.2 K under an external filed of 0.1 T parallel to the radiation. Black bars represent experimental error, red line indicates overall simulations, green line shows the Fe^{IV} component. In order to obtain better estimates of Fe^{IV}, two high-spin Fe^{III} species and one diamagnetic Fe^{III} (e.g. ferric dimer) were included into the simulation to cover the area, the parameters of which are tentative: $S = 5/2$, $D = -1.0$ cm^{-1}, $E/D = 0.30$, $\delta = 0.52$ mm/s, $\Delta E_Q = 1.36$ mm/s, $\eta = 0.0$, $A/g_n\beta_n = [-20.47, -21.0, -19.9]$ T, Area = 26%; $S = 5/2$, $D = -3.2$ cm^{-1}, $E/D = 0.33$, $\delta = 0.58$ mm/s, $\Delta E_Q = 1.27$ mm/s, $\eta = 0.4$, $A/g_n\beta_n = [-19.8, -22.7, -23.1]$ T, Area = 6%; $S = 0$, $\delta = 0.42$ mm/s, $|\Delta E_Q| = 1.10$ mm/s, Area = 4%.

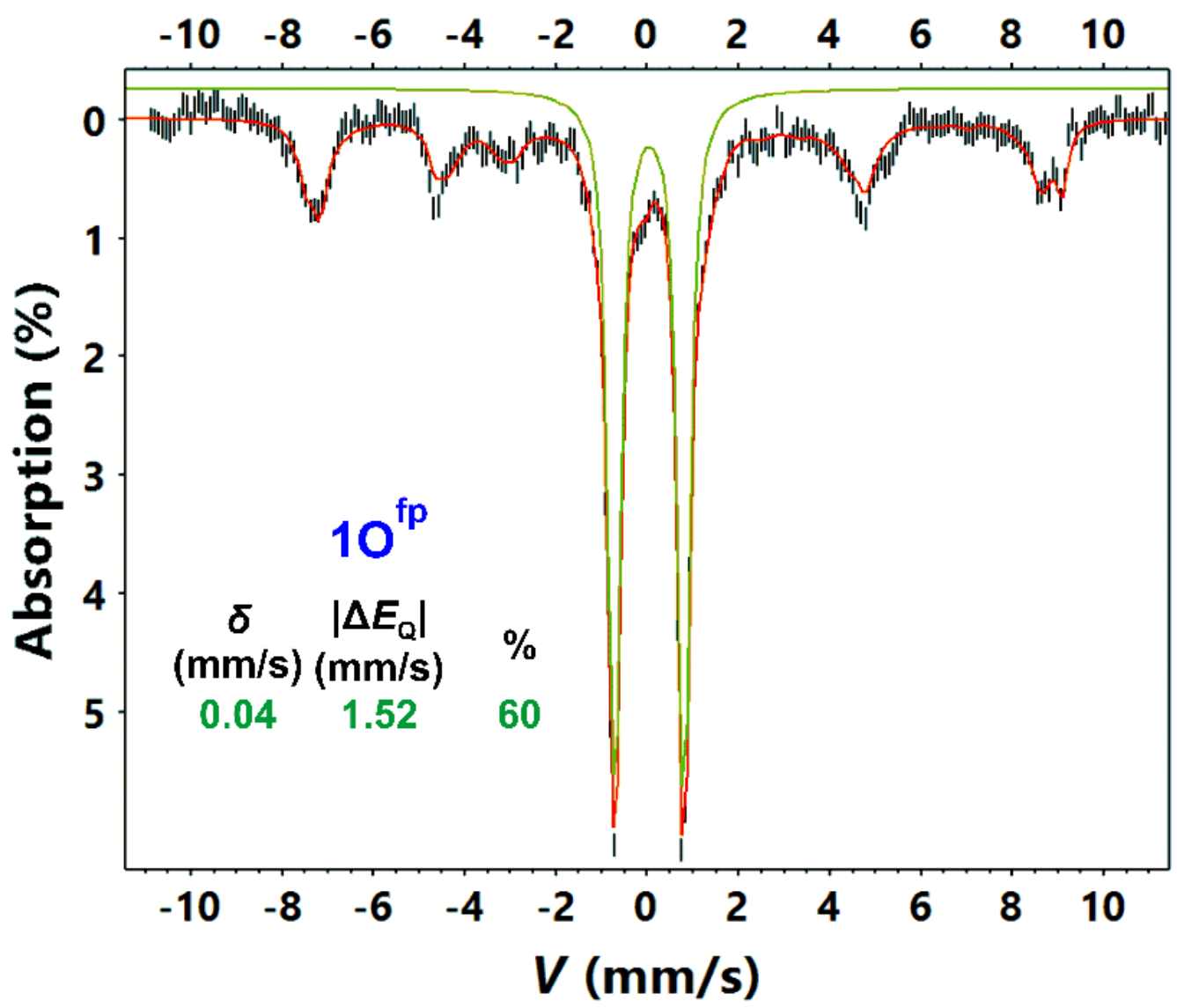

Figure S13 ^{57}Fe Mössbauer spectrum of ^{57}Fe enriched sample of **1O^{fp}** recorded at 4.2 K under an external filed of 0.1 T parallel to the radiation. Black bars represent experimental error, red line indicates overall simulations, green line shows the Fe^{IV} component. In order to obtain better estimates of Fe^{IV}, two high-spin Fe^{III} species and one diamagnetic Fe^{III} (e.g. ferric dimer) were included into the simulation to cover the area, the parameters of which are tentative: S = 5/2, D = -1.0 cm^{-1}, E/D = 0.32, δ = 0.50 mm/s, ΔE_Q = 1.48 mm/s, η = 0.3, $A/g_n\beta_n$ = [-20.5, -21.0, -18.6] T, Area = 19%; S = 5/2, D = -2.3 cm^{-1}, E/D = 0.32, δ = 0.43 mm/s, ΔE_Q = 0.55 mm/s, η = 0.2, $A/g_n\beta_n$ = [-20.7, -20.9, -19.9] T, Area = 21%; S = 0, δ = 0.59 mm/s, $|\Delta E_Q|$ = 1.29 mm/s, Area = 2%.

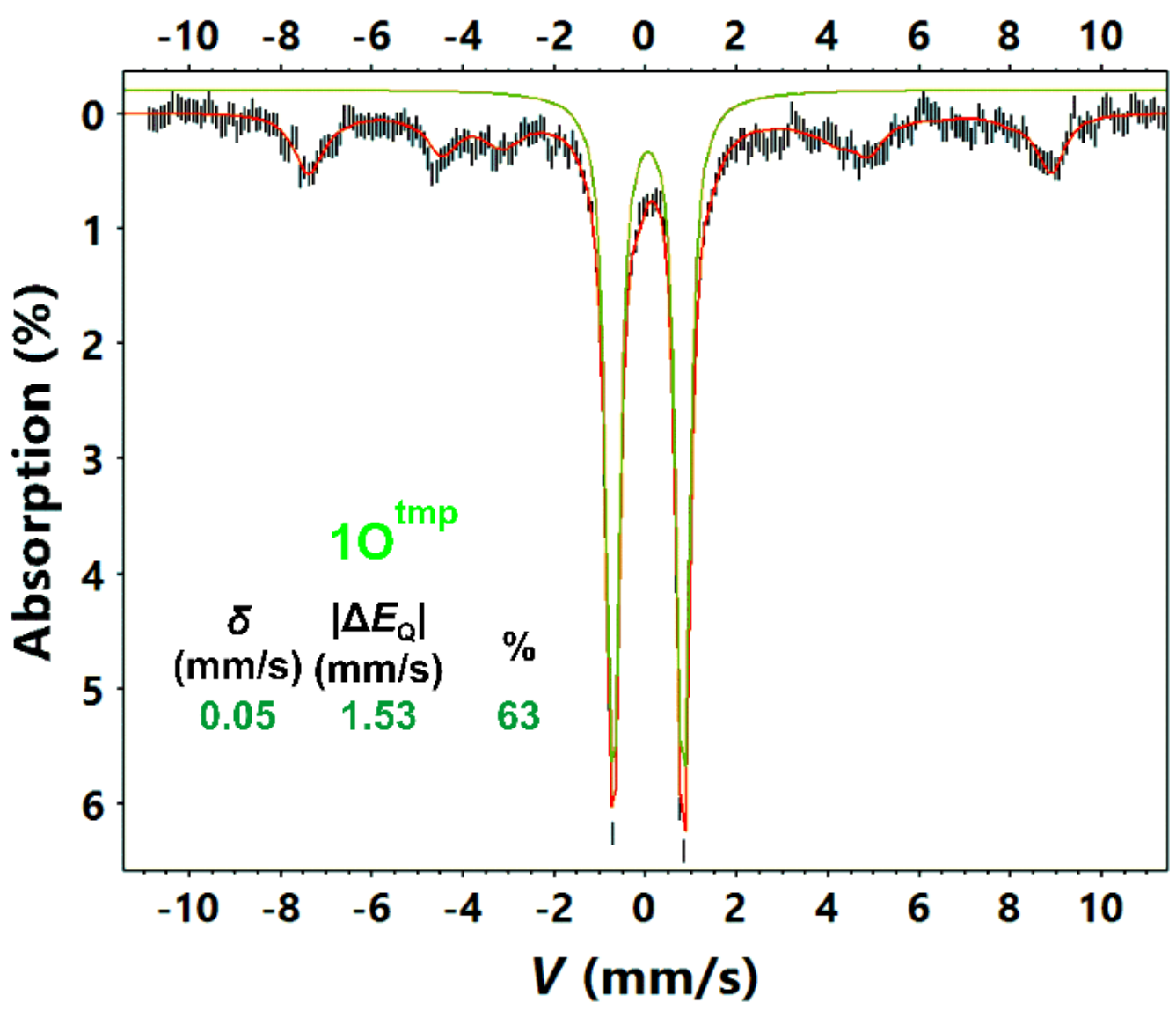

Figure S14 ^{57}Fe Mössbauer spectrum of ^{57}Fe enriched sample of **1O^{tmp}** recorded at 4.2 K under an external filed of 0.1 T parallel to the radiation. Black bars represent experimental error, red line indicates overall simulations, green line shows the Fe^{IV} component. In order to obtain better estimates of Fe^{IV}, one high-spin Fe^{III} species and one diamagnetic Fe^{III} (e.g. ferric dimer) were included into the simulation to cover the area, the parameters of which are tentative: S = 5/2, D = -1.2 cm^{-1}, E/D = 0.33, δ = 0.47 mm/s, ΔE_Q = 0.92 mm/s, η = 0.2, $A/g_n\beta_n$ = [-20.7, -21.1, -20.2] T, Area = 33%; S = 0, δ = 0.41 mm/s, $|\Delta E_Q|$ = 1.07 mm/s, Area = 3%.

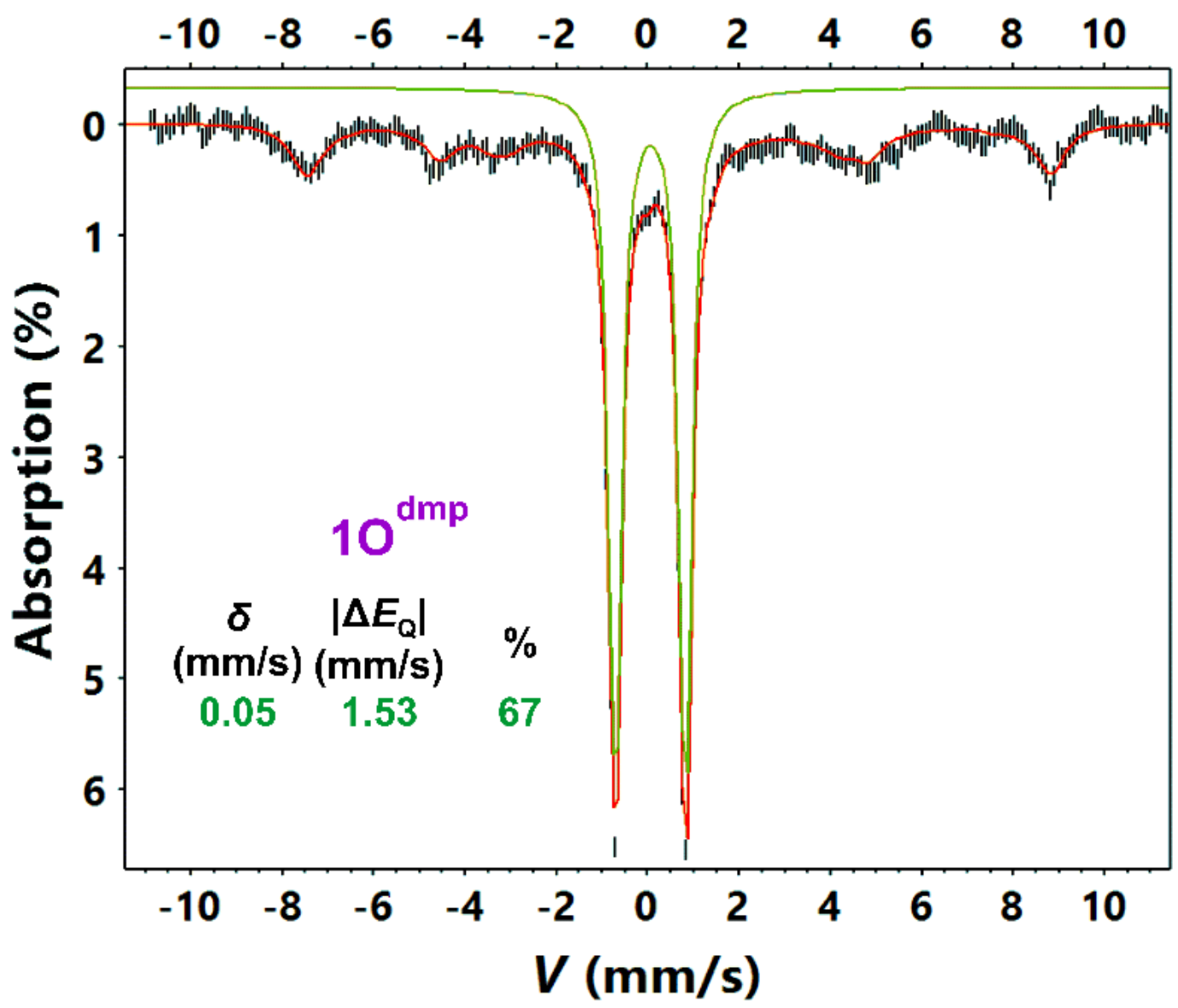

Figure S15 ^{57}Fe Mössbauer spectrum of ^{57}Fe enriched sample of **1O^{dmp}** recorded at 4.2 K under an external filed of 0.1 T parallel to the radiation. Black bars represent experimental error, red line indicates overall simulations, green line shows the Fe^{IV} component. In order to obtain better estimates of Fe^{IV}, one high-spin Fe^{III} species and one diamagnetic Fe^{III} (e.g. ferric dimer) were included into the simulation to cover the area, the parameters of which are tentative: $S = 5/2$, $D = -1.0$ cm^{-1}, $E/D = 0.33$, $\delta = 0.40$ mm/s, $\Delta E_Q = 1.12$ mm/s, $\eta = 0.0$, $A/g_n\beta_n$ = [-20.3, -21.1, -20.1] T, Area = 33%; $S = 0$, $\delta = 0.47$ mm/s, $|\Delta E_Q| = 0.95$ mm/s, Area = 1%.

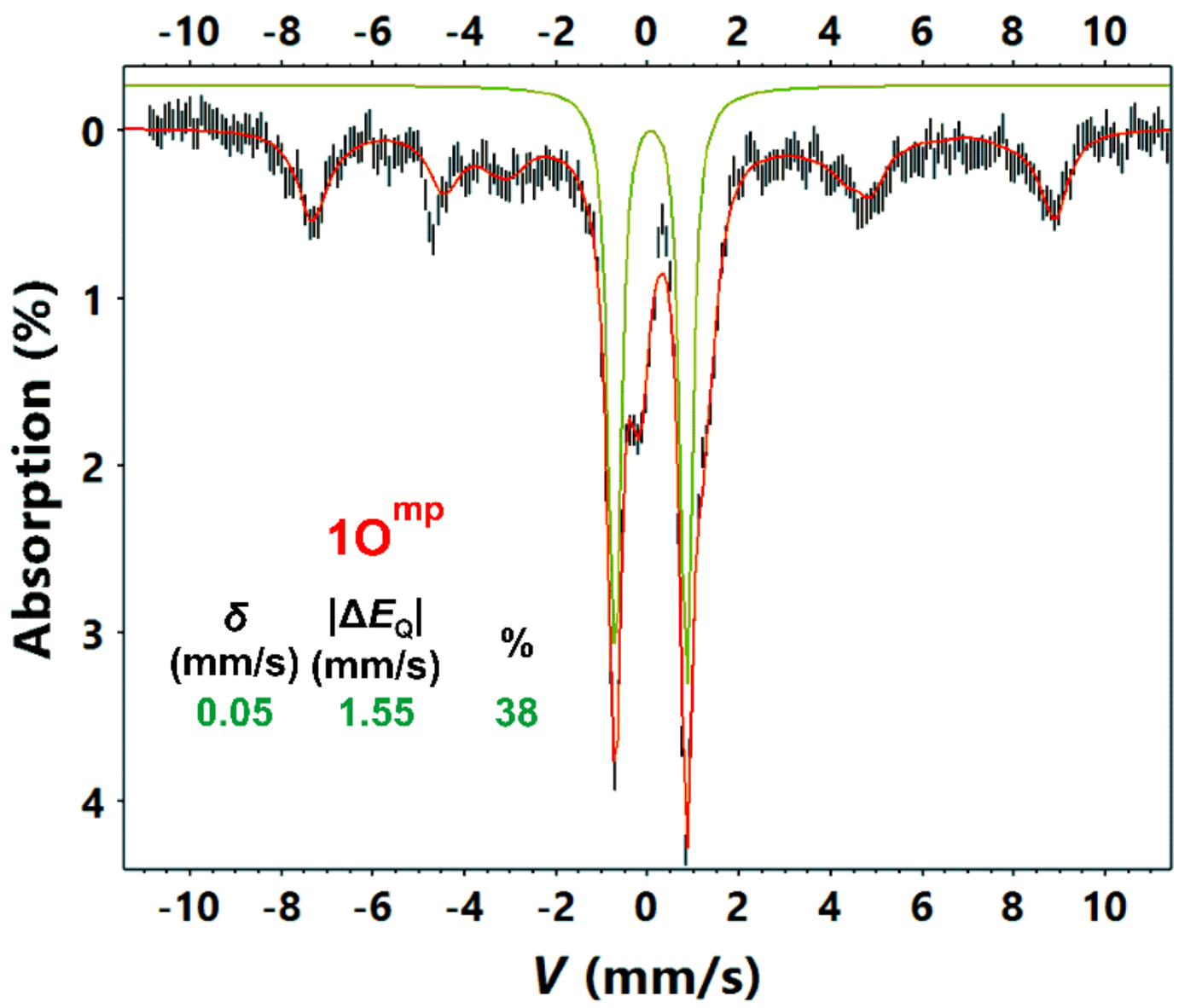

Figure S16 ^{57}Fe Mössbauer spectrum of ^{57}Fe enriched sample of **1O^{mp}** recorded at 4.2 K under an external filed of 0.1 T parallel to the radiation. Black bars represent experimental error, red line indicates overall simulations, green line shows the Fe^{IV} component. In order to obtain better estimates of Fe^{IV}, one high-spin Fe^{III} species and one diamagnetic Fe^{III} (e.g. ferric dimer) were included into the simulation to cover the area, the parameters of which are tentative: S = 5/2, D = -1.2 cm^{-1}, E/D = 0.32, δ = 0.49 mm/s, ΔE_Q = 1.11 mm/s, η = 0.1, $A/g_n\beta_n$ = [-20.7, -21.0, -20.2] T, Area = 39%; $S = 0$, $\delta = 0.50$ mm/s, $\Delta E_Q = 1.37$ mm/s, Area = 24%.

Table S5. Properties of the New $Fe^{IV}(O)$ Complexes Evaluated in This Work as well as Previously Reported Similar $Fe^{IV}(O)$ Complexes (Generated by Treating Iron(II) Precursors with 1-2 equiv. *s*-ArIO)

Complex	λ_{max}, nm	ε, $M^{-1}cm^{-1}$	δ, mm/s	(ΔE_Q), mm/s	S at 4.2 K	S at 193 K	$t_{1/2}$ (CH_3CN) at 233 K	k_2, ($M^{-1}s^{-1}$) c-C_6H_{12}	k_2, ($M^{-1}s^{-1}$) Toluene	Reference
**4O**	650; 900	300; 75	0.24	-1.05	2	2	15 min	0.37	0.64	[3]
**3O**	770	200	0.03	1.62	1	1	2 min	0.24	0.47	[12]
$\mathbf{2O}^{c\text{-propyl}}$	772	418	0.04	1.60	1	1	43 s	0.53	0.92	[5]
$\mathbf{2O}^{c\text{-butyl}}$	771	387	0.04	1.60	1	1	38 s	0.62	1.10	[5]
$\mathbf{2O}^{c\text{-pentyl}}$	773	342	0.04	1.60	1	1	33 s	1.08	1.64	[5]
$\mathbf{2O}^{c\text{-hexyl}}$	765	310	0.08	1.56	1	1	30 s	1.75	3.11	[5]
$\mathbf{2O}^{c\text{-oxanyl}}$	773	389	0.05	1.55	1	1	36 s	1.56	2.58	[5]
$\mathbf{2O}^{\mathbf{adamantyl}}$	767	387	0.04	1.60	1	1	37 s	1.22	1.96	[5]
$\mathbf{1O}^{\mathbf{pfp}}$	774	383	0.05	1.52	1	1	219 s	0.46	0.68	This Work
$\mathbf{1O}^{\mathbf{dfp}}$	774	407	0.05	1.53	1	1	142 s	0.52	0.99	This Work
$\mathbf{1O}^{\mathbf{fp}}$	771	376	0.04	1.52	1	1	33 s	1.07	2.32	This Work
$\mathbf{1O}^{\mathbf{mp}}$	772	296	0.05	1.55	1	1	30 s	1.07	2.26	This Work
$\mathbf{1O}^{\mathbf{dmp}}$	767	399	0.05	1.53	1	1	85 s	0.62	1.13	This Work
$\mathbf{1O}^{\mathbf{tmp}}$	764	426	0.05	1.53	1	1	95 s	0.38	0.78	This Work

The k_2 values were measured at 233 K for hydrogen atom abstraction reactions of the oxoiron(IV) oxidants with external substrates.

D) Reactivity and kinetics studies of oxoiron(IV) complexes.

All experiments were conducted with 1-1.5 mL solutions of 1 mM iron(II) complexes in MeCN solutions at given temperatures for HAT (hydrogen-atom-transfer) substrates. The oxoiron(IV) complexes were generated using 1-2 eq. ArIO in 2,2,2-trifluoroethanol or DCM-d_2. The pseudo-first order decay curves from absorption-time profiles were obtained by monitoring the decrease in absorbance for the oxoiron(IV) complexes with time, from their absorbance spectra, when a known concentration of substrate is introduced. These profiles at various concentrations of substrates were fit with a first order exponential decay equation. The rates obtained (k_{obs}) were then plotted against different concentrations of substrates, and their slopes represent 2nd order rate constants (k_2). To isolate and identify the organic products after the reaction of $\mathbf{1O}^{\mathbf{R}}$ (1mM) with cyclohexane (100 mM), the reaction mixtures were allowed to rise to room temperature, esterified with acetic anhydride (1 mL) and 1-methylimidazole (0.1 mL), and subsequently filtered through a silica plug, eluting with ethyl acetate. The filtered solutions were then analyzed using gas chromatography with naphthalene as the internal standard.

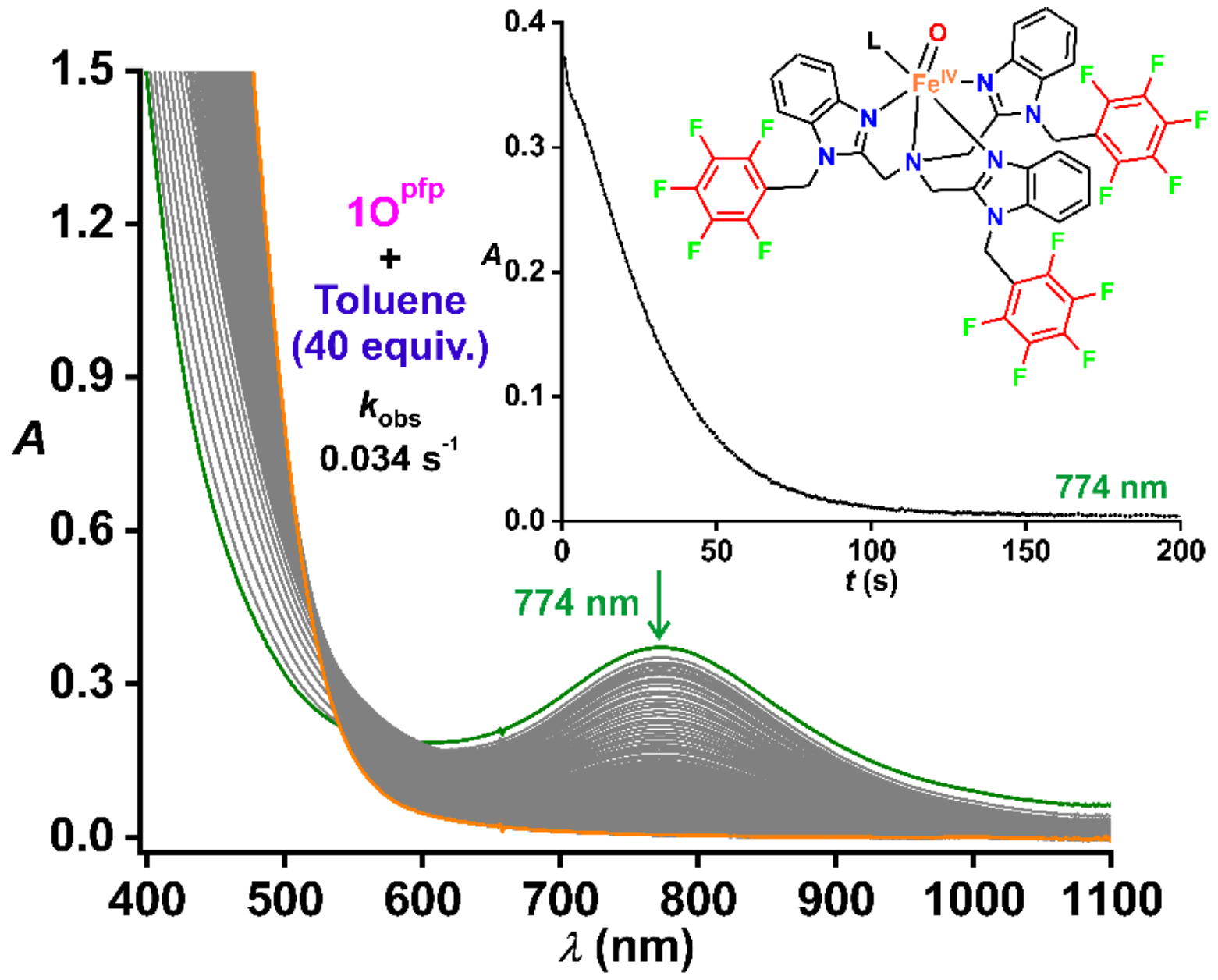

Figure S17 Decay of $\mathbf{1O^{pfp}}$ in the presence of toluene (40 equiv.) in CH_3CN at 233 K. Inset: time trace for the band at 774 nm.

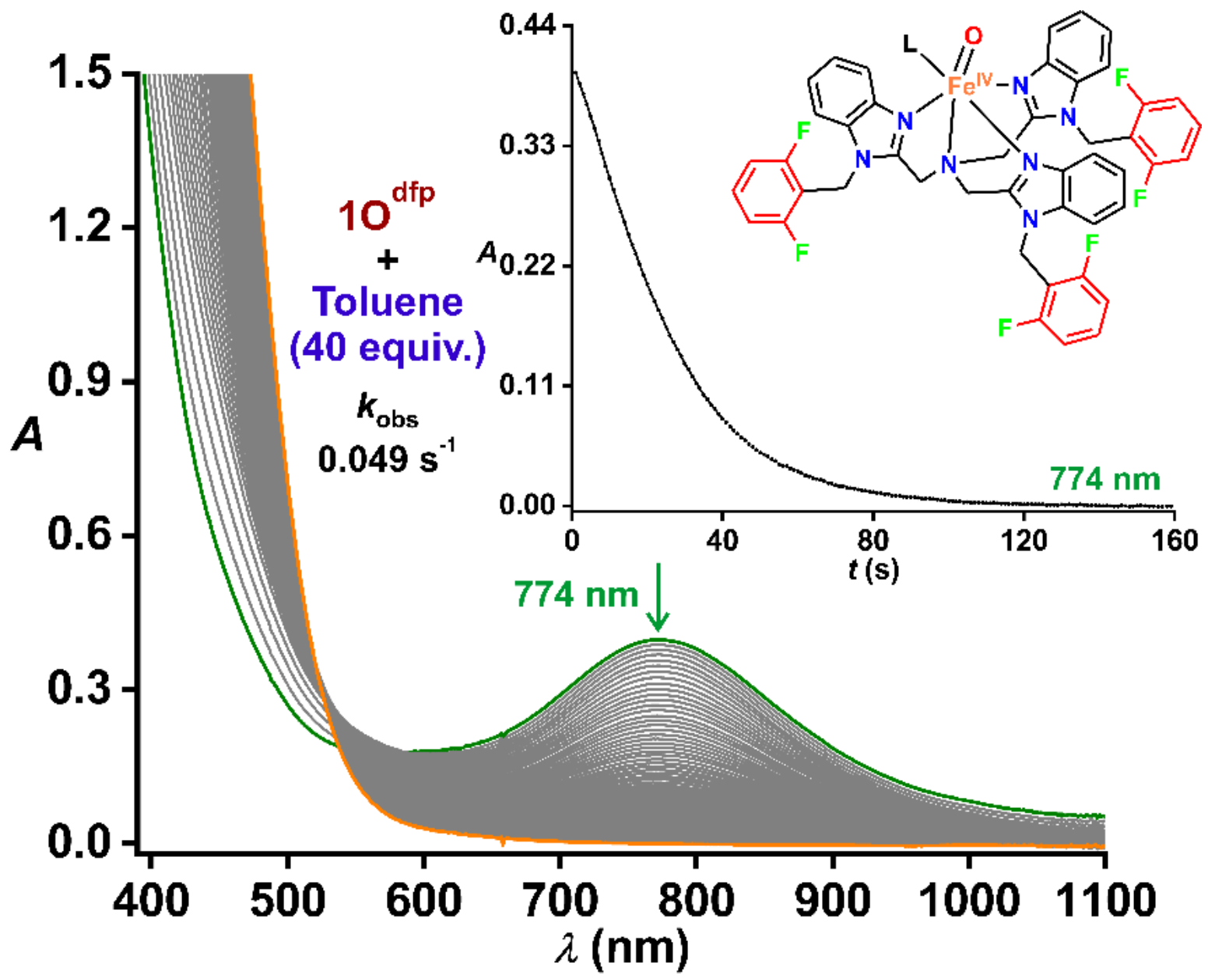

Figure S18 Decay of $\mathbf{1O^{dfp}}$ in the presence of toluene (40 equiv.) in CH_3CN at 233 K. Inset: time trace for the band at 774 nm.

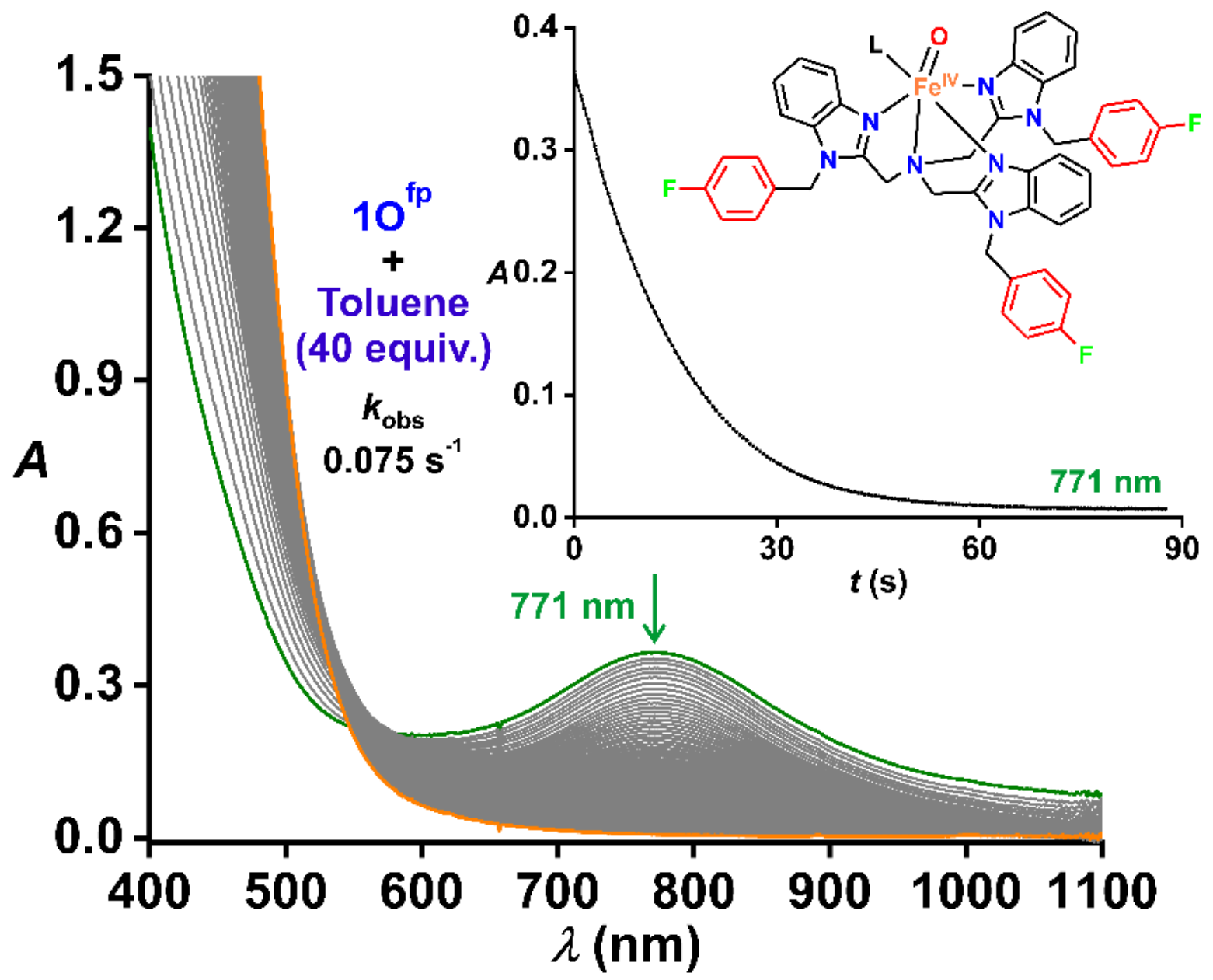

Figure S19 Decay of **1O**^{fp} in the presence of toluene (40 equiv.) in CH_3CN at 233 K. Inset: time trace for the band at 771 nm.

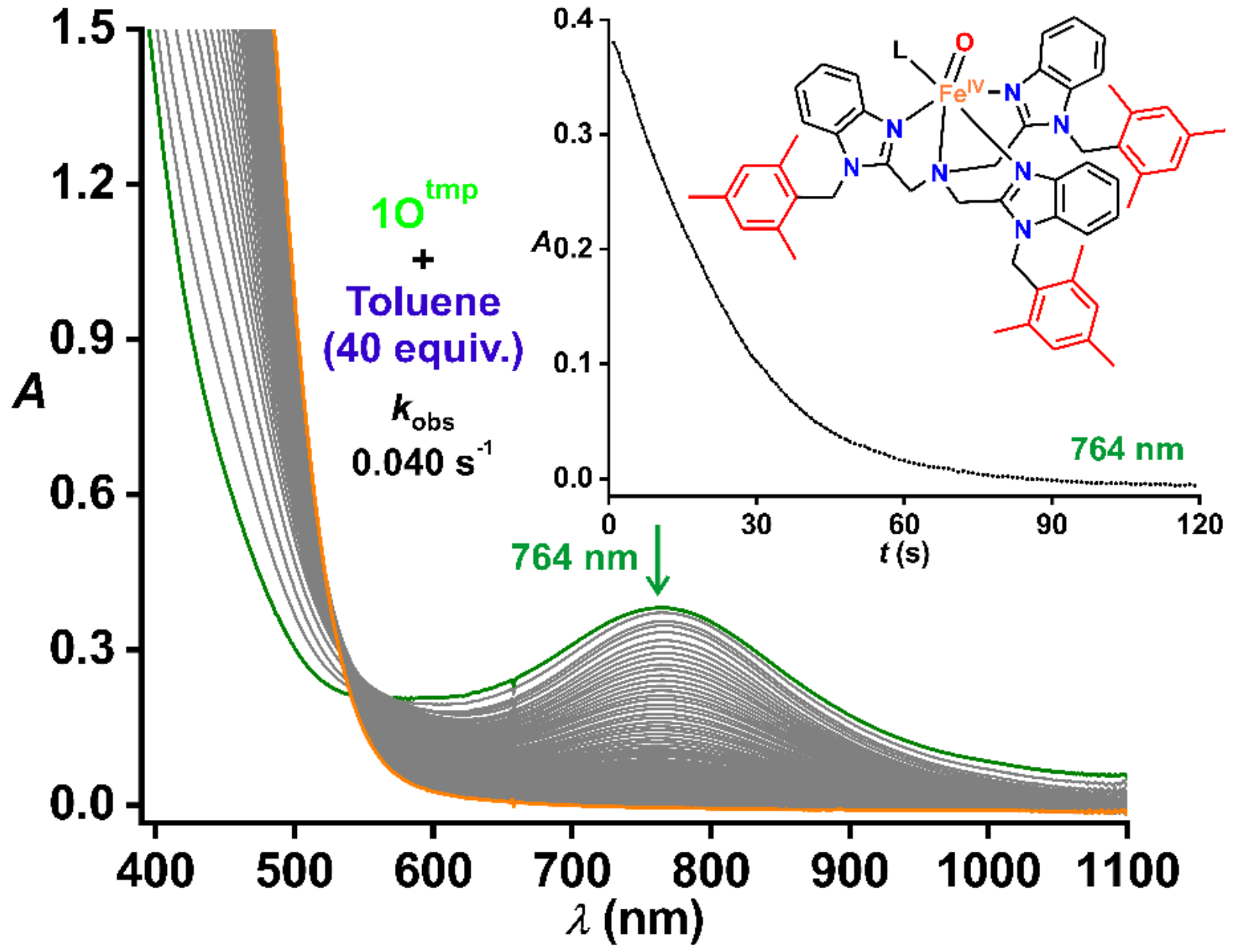

Figure S20 Decay of **1O**^{tmp} in the presence of toluene (40 equiv.) in CH_3CN at 233 K. Inset: time trace for the band at 764 nm.

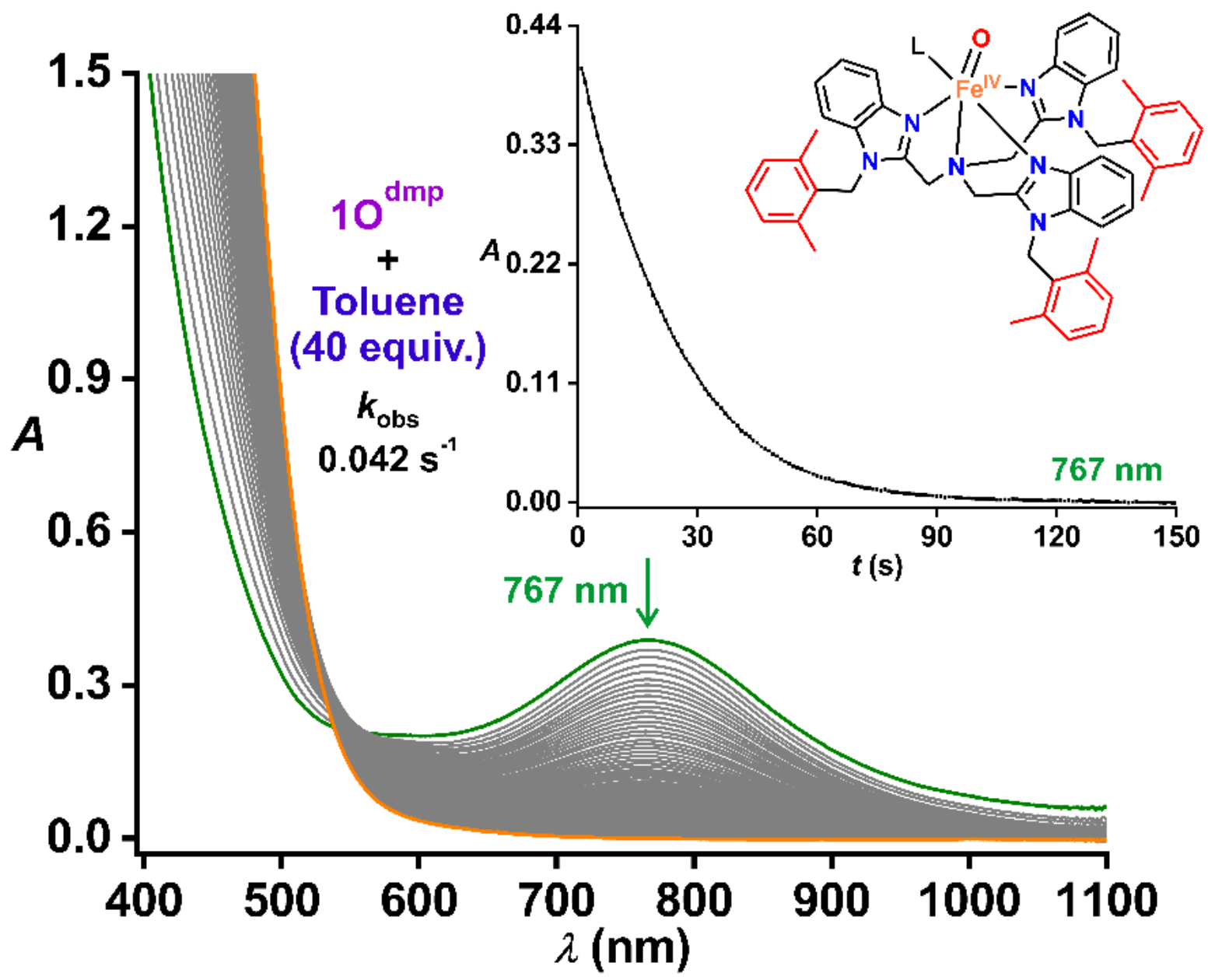

Figure S21 Decay of **1O**$^{\mathbf{dmp}}$ in the presence of toluene (40 equiv.) in CH_3CN at 233 K. Inset: time trace for the band at 767 nm.

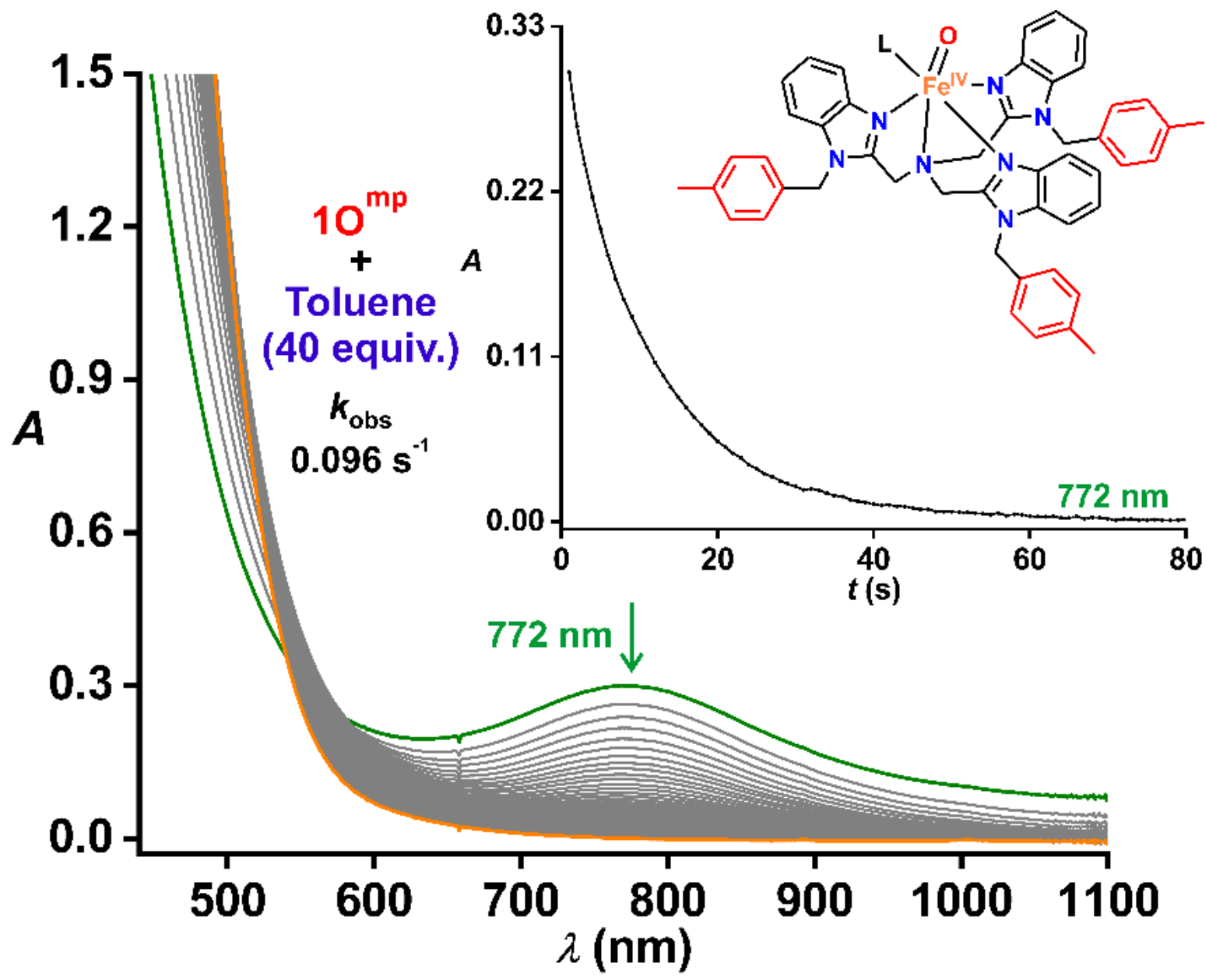

Figure S22 Decay of **1O**$^{\mathbf{mp}}$ in the presence of toluene (40 equiv.) in CH_3CN at 233 K. Inset: time trace for the band at 772 nm.

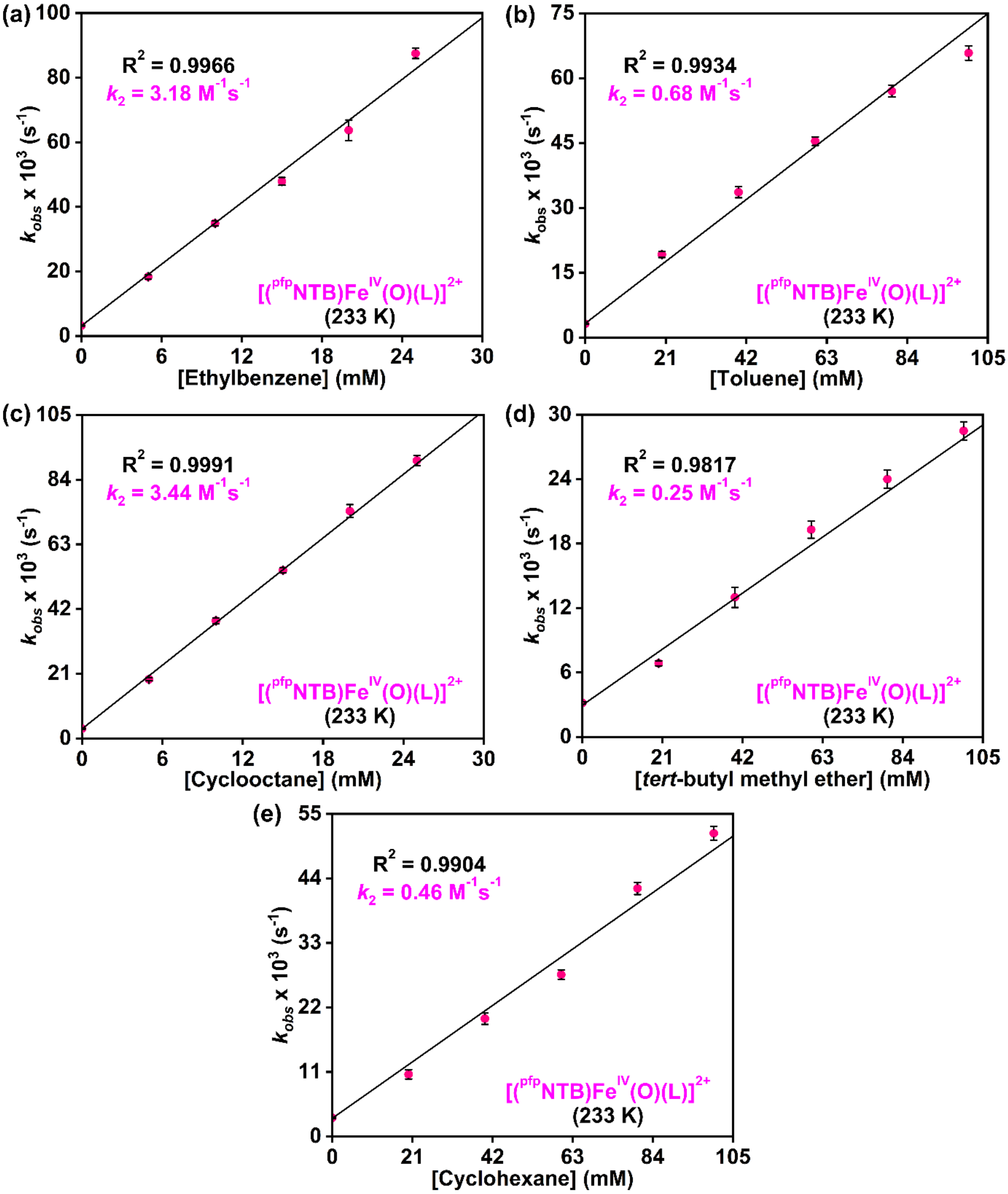

Figure S23 Plots of k_{obs} vs concentration of substrate for the reaction of $\mathbf{1O^{pfp}}$ with (a) ethylbenzene, (b) toluene, (c) cyclooctane, (d) *tert*-butyl methyl ether (TBME) and (e) cyclohexane at 233 K. Error bars represent ± SD (n=3).

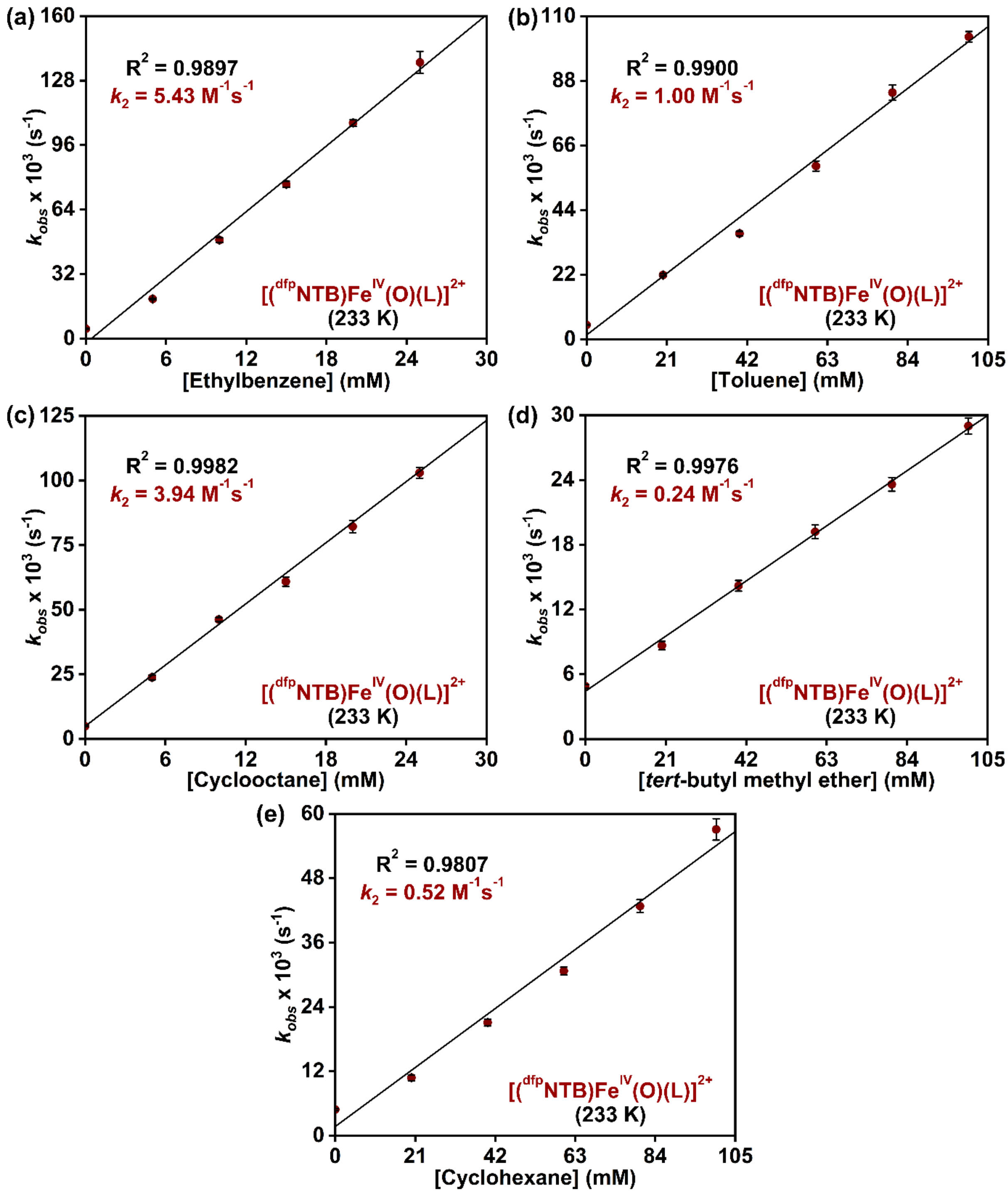

Figure S24 Plots of k_{obs} vs concentration of substrate for the reaction of **1O^{dfp}** with (a) ethylbenzene, (b) toluene, (c) cyclooctane, (d) *tert*-butyl methyl ether (TBME) and (e) cyclohexane at 233 K. Error bars represent ± SD (n=3).

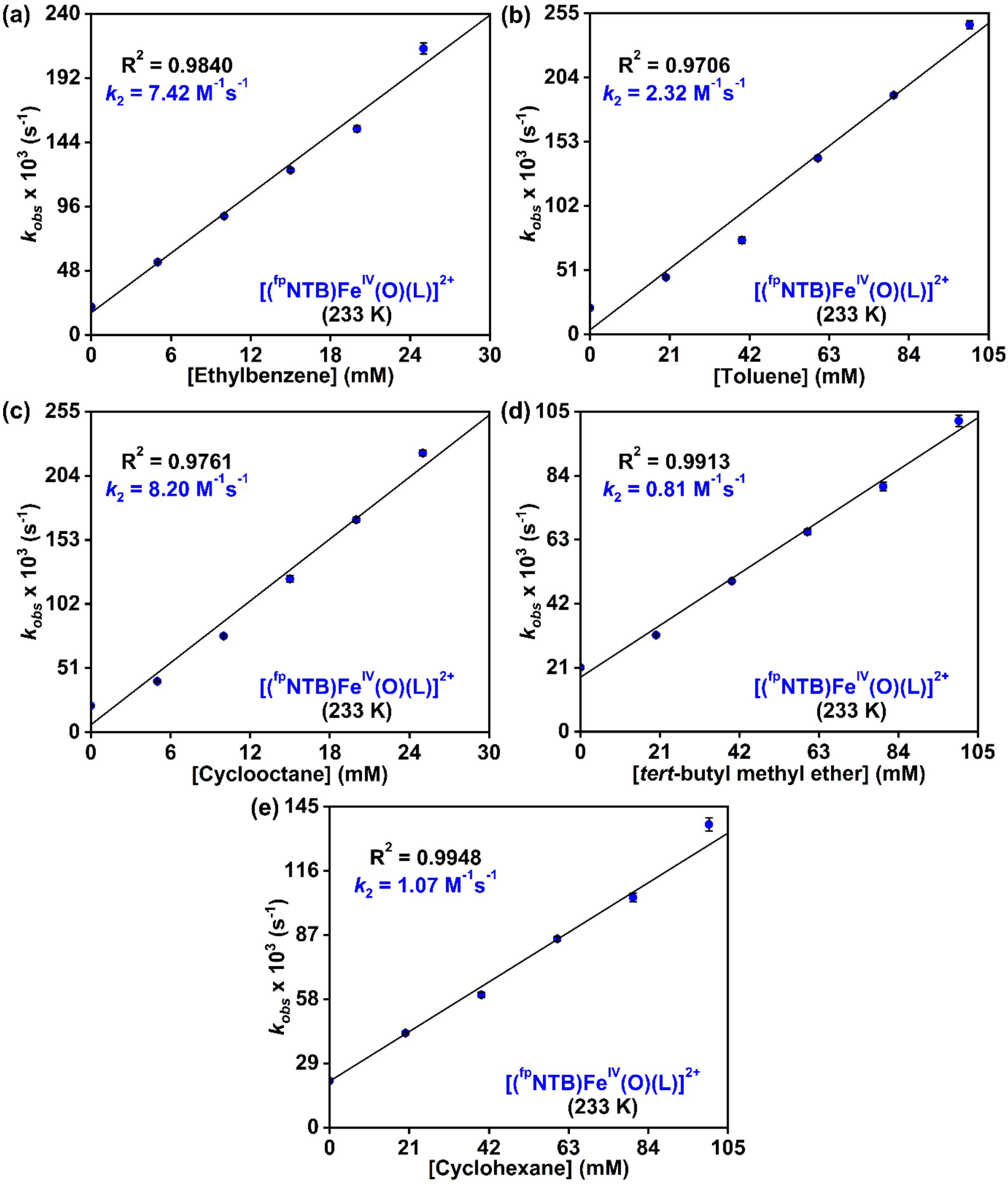

Figure S25 Plots of k_{obs} vs concentration of substrate for the reaction of **1O^{fp}** with (a) ethylbenzene, (b) toluene, (c) cyclooctane, (d) *tert*-butyl methyl ether (TBME) and (e) cyclohexane at 233 K. Error bars represent ± SD (*n*=3).

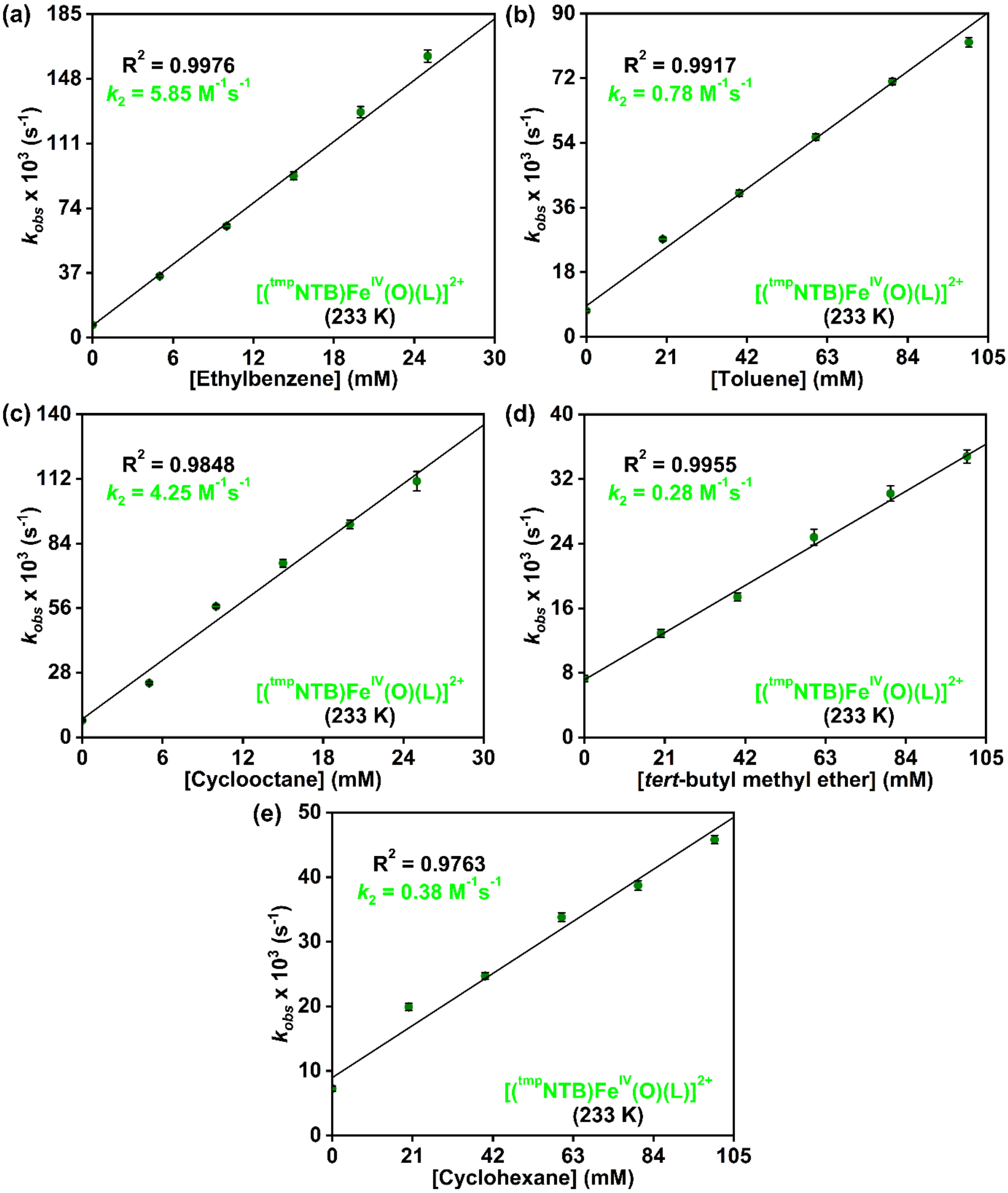

Figure S26 Plots of k_{obs} vs concentration of substrate for the reaction of $\mathbf{1O^{tmp}}$ with (a) ethylbenzene, (b) toluene, (c) cyclooctane, (d) *tert*-butyl methyl ether (TBME) and (e) cyclohexane at 233 K. Error bars represent ± SD (n=3).

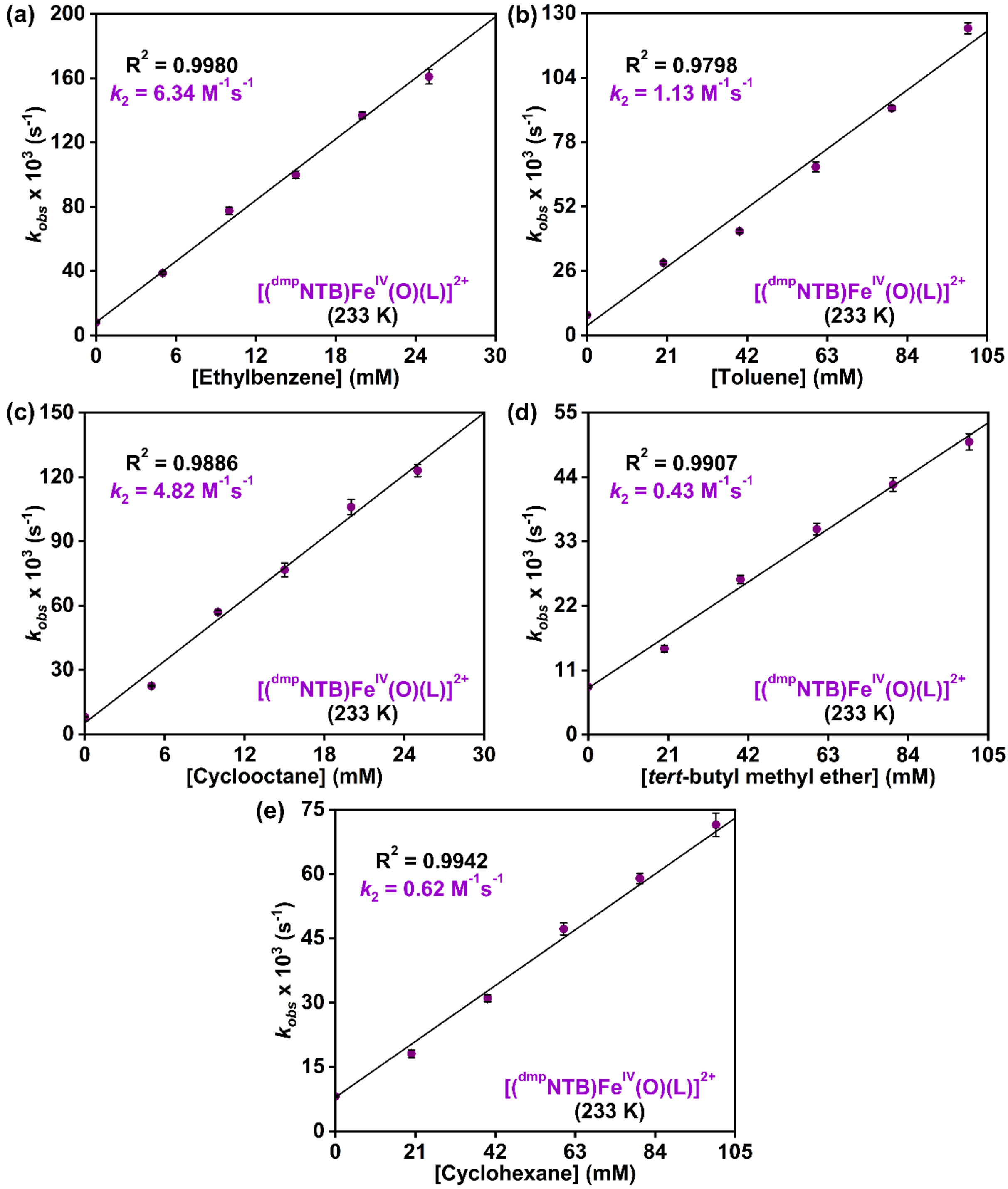

Figure S27 Plots of k_{obs} vs concentration of substrate for the reaction of $\mathbf{1O^{dmp}}$ with (a) ethylbenzene, (b) toluene, (c) cyclooctane, (d) *tert*-butyl methyl ether (TBME) and (e) cyclohexane at 233 K. Error bars represent ± SD (*n*=3).

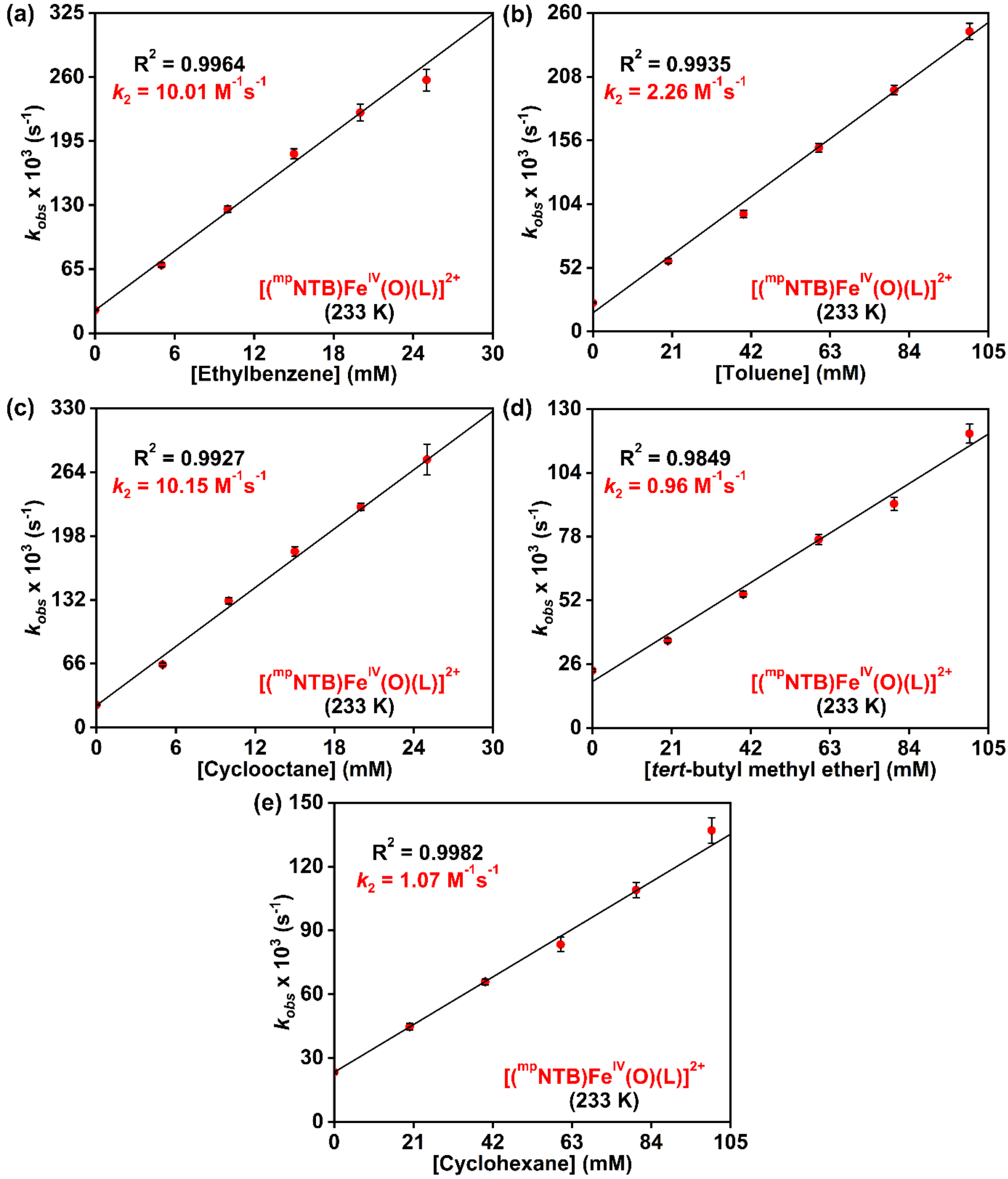

Figure S28 Plots of k_{obs} vs concentration of substrate for the reaction of **1O^{mp}** with (a) ethylbenzene, (b) toluene, (c) cyclooctane, (d) *tert*-butyl methyl ether (TBME) and (e) cyclohexane at 233 K. Error bars represent ± SD (n=3).

Table S6 Comparison of k_2 ($M^{-1}s^{-1}$) values in the oxidation of different substrates.

Complex	PhEt	PhMe	C_8H_{16}	TBME	*c*-C_6H_{12}
**1O^{pfp}**	3.181 ± 0.083	0.682 ± 0.025	3.443 ± 0.046	0.248 ± 0.015	0.458 ± 0.020
**1O^{dfp}**	5.429 ± 0.248	0.999 ± 0.045	3.944 ± 0.074	0.243 ± 0.005	0.524 ± 0.033
**1O^{fp}**	7.415 ± 0.422	2.319 ± 0.180	8.203 ± 0.572	0.810 ± 0.033	1.068 ± 0.034
**1O^{tmp}**	5.845 ± 0.129	0.777 ± 0.032	4.250 ± 0.236	0.278 ± 0.008	0.384 ± 0.027
**1O^{dmp}**	6.337 ± 0.127	1.132 ± 0.072	4.823 ± 0.232	0.432 ± 0.019	0.620 ± 0.021
**1O^{mp}**	10.01 ± 0.269	2.258 ± 0.081	10.15 ± 0.390	0.961 ± 0.053	1.065 ± 0.020
[a]**2O$^{c\text{-propyl}}$**	3.010 ± 0.071	0.916 ± 0.042	3.494 ± 0.170	0.215 ± 0.006	0.534 ± 0.013
[a]**2O$^{c\text{-butyl}}$**	3.647 ± 0.262	1.101 ± 0.057	3.774 ± 0.111	0.297 ± 0.015	0.617 ± 0.050
[a]**2O$^{c\text{-pentyl}}$**	6.715 ± 0.188	1.642 ± 0.067	4.497 ± 0.069	0.387 ± 0.019	1.079 ± 0.087
[a]**2O^{adamantyl}**	8.450 ± 0.386	1.956 ± 0.077	4.657 ± 0.199	0.531 ± 0.038	1.223 ± 0.052
[a]**2O^{oxanyl}**	12.43 ± 0.444	2.577 ± 0.130	8.894 ± 0.391	0.683 ± 0.038	1.556 ± 0.099
[a]**2O$^{c\text{-hexyl}}$**	13.08 ± 0.617	3.106 ± 0.299	9.803 ± 0.706	0.893 ± 0.018	1.748 ± 0.046
[b]**3O**	1.5	0.47	2.2	-	0.25
[c]**4O**	2.1	0.64	3.5	0.15	0.37

[a] Data were taken from reference[5], [b] Data were taken from reference[12], [c] Data were taken from reference[3].

Table S7. Comparison of hydrocarbon oxidation rates with other previously reported nonheme oxoiron(IV) complexes. All k_2 values are corrected towards 233 K for comparison.

Complex	Spin-State	k_2 (Toluene)	k_2 (*c*-C_6H_{12})	Reference
[a] $[Fe^{IV}(O)(N2Py2Pz)]^{2+}$	$S = 1$ (Oh)	1.56 x 10^{-3} (*0.15*)	3.0 x 10^{-3} (*0.29*)	[13]
[b] $[Fe^{IV}(O)(TPA)(MeCN)]^{2+}$	$S = 1$ (Oh)	3.8 x 10^{-4} (*0.0061*)	1.7 x 10^{-4} (*0.0027*)	[14]
[c] $[Fe^{IV}(O)(TMCO)(OTf)]^{+}$	$S = 1$ (Oh)	0.05 (1.3 x 10^{-2})	0.02 (4.7 x 10^{-3})	[15]
[d] $[Fe^{IV}(O)(^tBu_3tacn)]^{2+}$	$S = 2$ (Td)	0.46 (*0.057*)	Not Reported	[16]
[e] $[Fe^{IV}(O)(L1\text{-bispidine})(MeCN)]^{2+}$	$S = 1$ (Oh)	N. R.	N. R.	[17-18]
[f] $[Fe^{IV}(O)(L1\text{-bispidine})(Cl)]^{+}$	$S = 1$ (Oh)	N. R.	2.4 x 10^{4} (*7.6 x 10^{2}*)	[18-19]
$[Fe^{IV}(O)(Me_3NTB)(MeCN)]^{2+}$ (**3O**)	$S = 1$ (Oh)	0.47	0.25	[12]
$[Fe^{IV}(O)(TQA)(MeCN)]^{2+}$ (**4O**)	$S = 2$ (Oh)	0.64	0.37	[3]
**2O$^{c\text{-hexyl}}$**	$S = 1$ (Oh)	3.11	1.75	[5]
**1O^{fp}**	$S = 1$ (Oh)	2.32	1.07	This Work
**1O^{mp}**	$S = 1$ (Oh)	2.26	1.07	This Work

Experimental substrate oxidation rates mentioned within brackets and italics are reported at [a] 298 K, [b] 273 K, [c] 213 K, [d] 203 K, [f] 183 K and corrected for the temperature difference by doubling the rate for every 10 degrees rise in temperature. [e] k_2 value of 0.29 $M^{-1}s^{-1}$ reported for BzOH (benzyl alcohol) oxidation at 238 K. [N.B.: N.R. (not reported)]

Table S8. Experimental yields for cyclohexane oxidation products for **$1O^R$** complexes.

Complex	Cyclohexanol (equiv./Fe[a])	Cyclohexanone (equiv./Fe[a])
**$1O^{pfp}$**	0.18	0.21
**$1O^{dfp}$**	0.16	0.22
**$1O^{fp}$**	0.20	0.24
**$1O^{tmp}$**	0.15	0.18
**$1O^{dmp}$**	0.18	0.20
**$1O^{mp}$**	0.19	0.20

[a] Fe corresponds to the amount of 1^R used to generate **$1O^R$**.

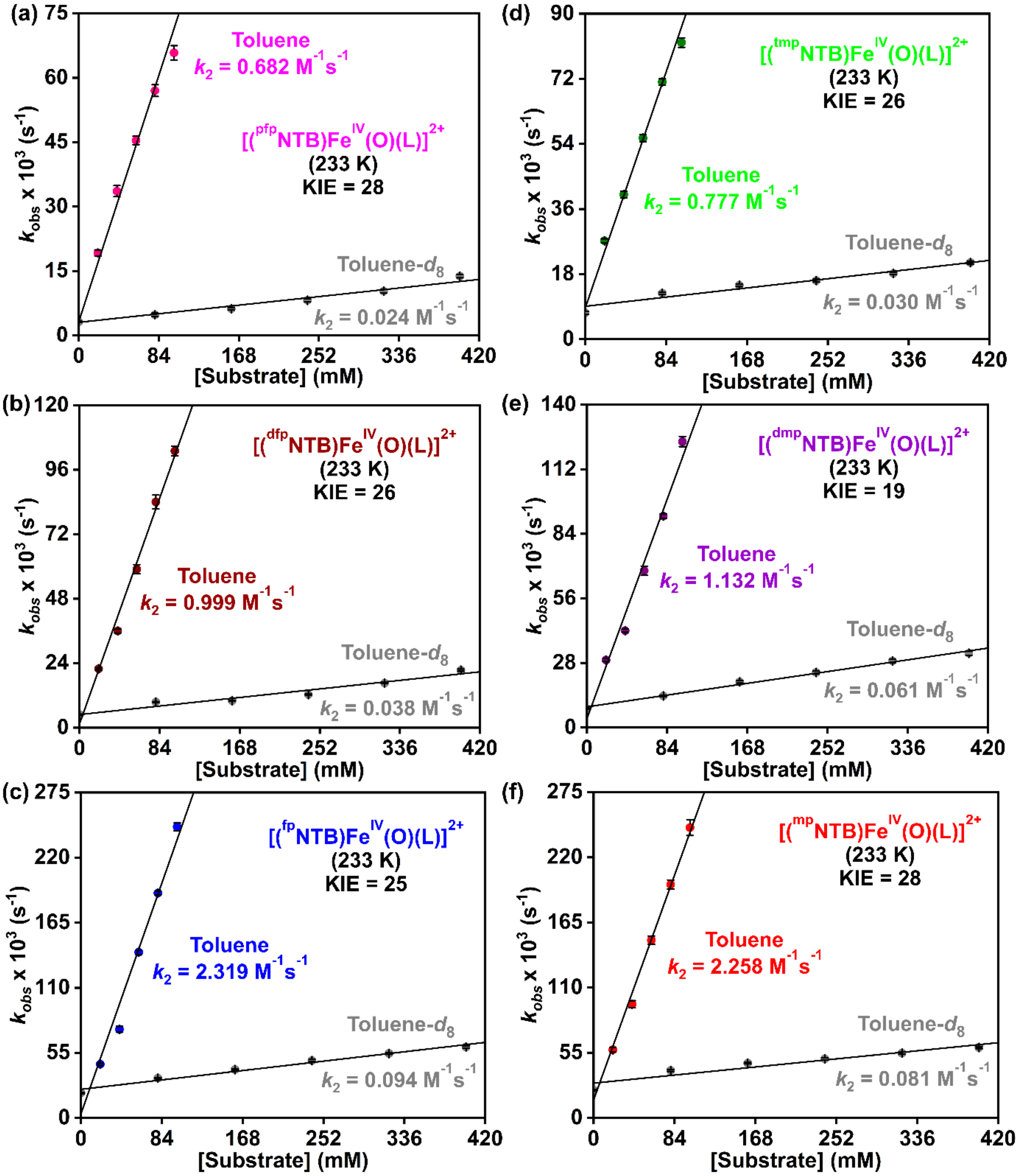

Figure S29 Kinetic isotope effect [obtained from the ratio of k_2 values of toluene (colour) and toluene-d_8 (grey) oxidation] in toluene oxidation by (a) $\mathbf{1O^{pfp}}$, (b) $\mathbf{1O^{dfp}}$, (c) $\mathbf{1O^{fp}}$, (d) $\mathbf{1O^{tmp}}$, (e) $\mathbf{1O^{dmp}}$ and (f) $\mathbf{1O^{mp}}$. Error bars represent ± SD (n=3).

E) <u>NMR spectroscopy of oxoiron(IV) complexes.</u>

1. **Generation of the ferryl complexes for NMR spectroscopic analysis**

4-mM / 2-mM stock solutions of starting iron(II) precursors were made within an air-free nitrogen-filled glovebox in acetonitrile-d_3 or acetone-d_6. Samples of the oxoiron(IV) complexes for NMR analysis were generated by transferring 0.5 mL of a precursor solution into an NMR tube (in glove box) and cooling it to the desired temperature in the NMR instrument for analysis; at this point a ^{1}H NMR spectrum of the iron(II) precursor was obtained, after locking to the residual proton signal in the deuterated solvent, tuning to the intended nucleus, and then shimming to obtain a uniform magnetic field around the sample. The following three steps were then performed in quick succession: 1) the NMR tube was ejected from the instrument, 2) 2 equivalents of ArIO in either 2,2,2-trifluoroethanol (TFE) or DCM-d_2 were quickly injected to generate the oxoiron(IV) intermediate; the tube was recapped with either a septum or its plastic cap (to minimize decay of the species) and 3) the tube was immediately inserted back into the instrument for data collection. Because the instrument had been locked to the solvent as well as tuned to the nucleus of interest, only quick shimming was performed again before collection of NMR data for the oxoiron(IV) species.

2. **General parameters for data collection and processing**

The following parameters were used to acquire the ^{1}H NMR data for paramagnetic compounds: acquisition time = 0.064 s / 0.032 s; relaxation delay = 0.03 s; sweep width = 400 ppm offset (centered) at 6 ppm; line broadening factor = 10-30 Hz. Chemical shifts (ppm) were referenced to residual protic solvent peaks. The NMR spectra obtained were processed using the NMR processing software MestReNova 12.0 or Bruker's Top-Spin 3.5pl7.

3. **NMR spectra of $1O^R$ complexes**

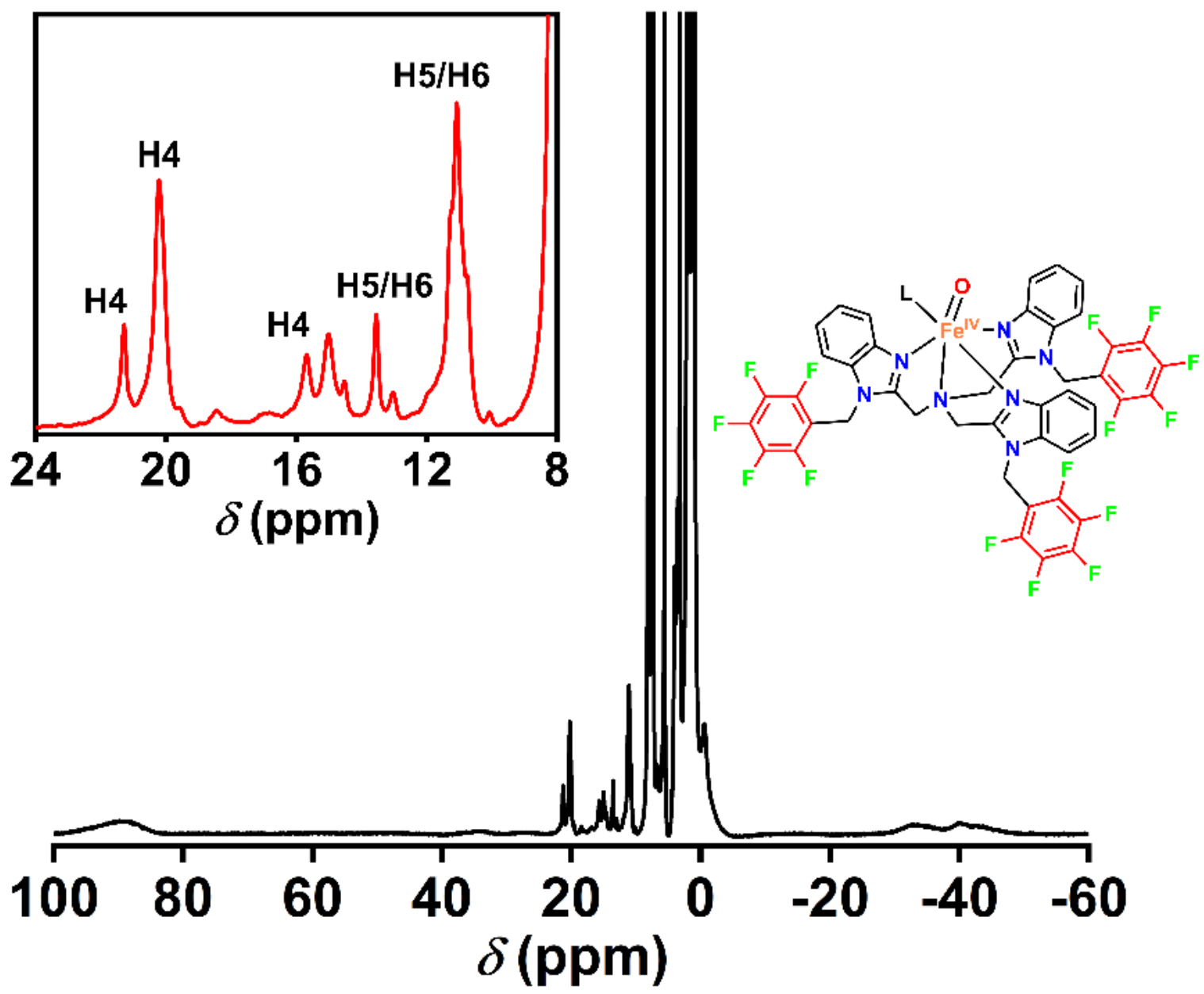

Figure S30 ^{1}H NMR spectra of complex **$1O^{pfp}$** [prepared by adding 2 equiv. of s-ArIO to a 2-mM solution made by dissolving solid **1^{pfp}** in acetone-d_6 : acetonitrile-d_3 (9:1)] at 193 K. Insets show the expanded region from 8 to 24 ppm.

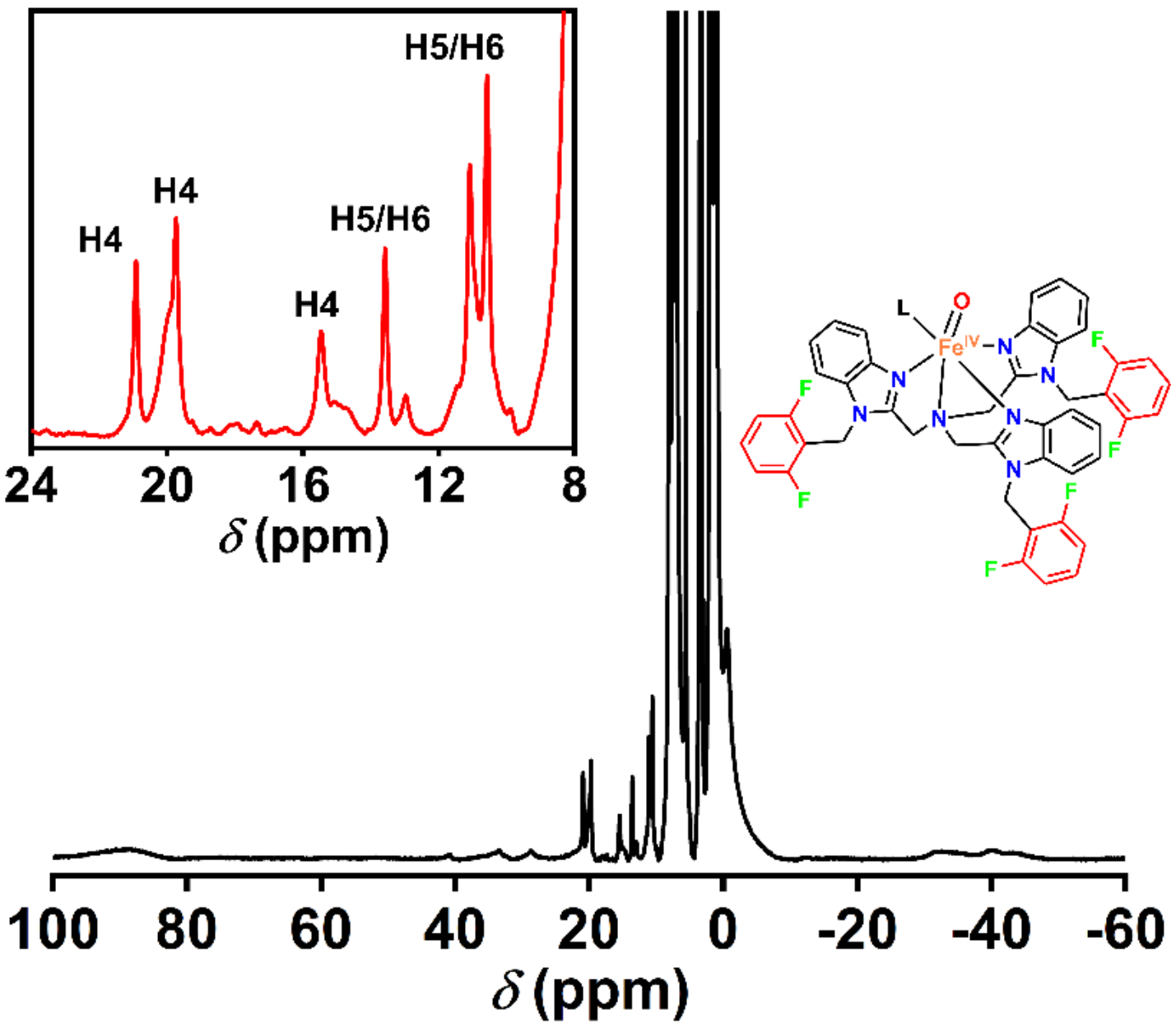

Figure S31 ^{1}H NMR spectra of complex **1O^{dfp}** [prepared by adding 2 equiv. of s-ArIO to a 2-mM solution made by dissolving solid **1^{dfp}** in acetone-d_6 : acetonitrile-d_3 (9:1)] at 193 K. Insets show the expanded region from 8 to 24 ppm.

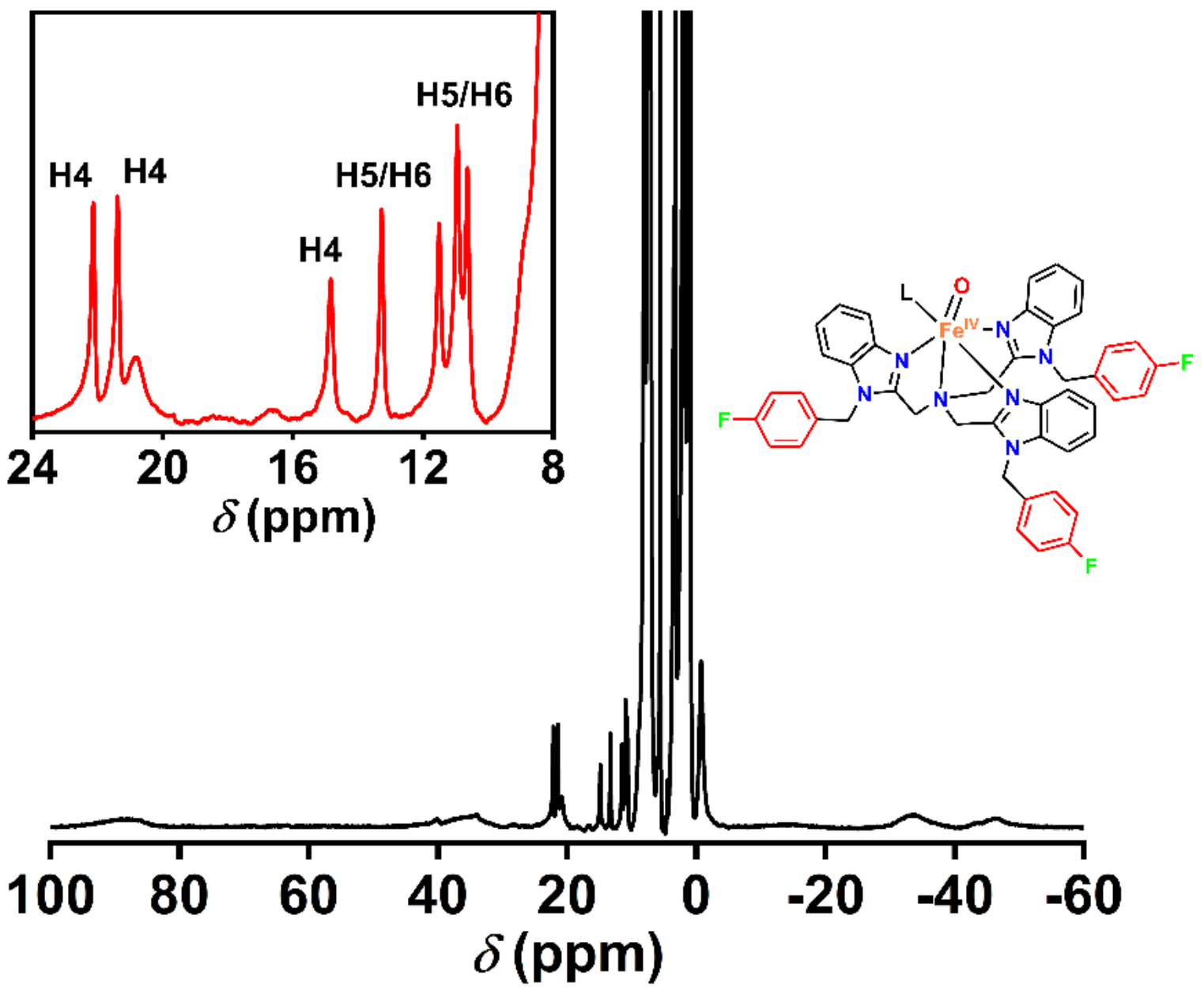

Figure S32 ^{1}H NMR spectra of complex **1O^{fp}** [prepared by adding 2 equiv. of s-ArIO to a 2-mM solution made by dissolving solid **1^{fp}** in acetone-d_6 : acetonitrile-d_3 (9:1)] at 193 K. Insets show the expanded region from 8 to 24 ppm.

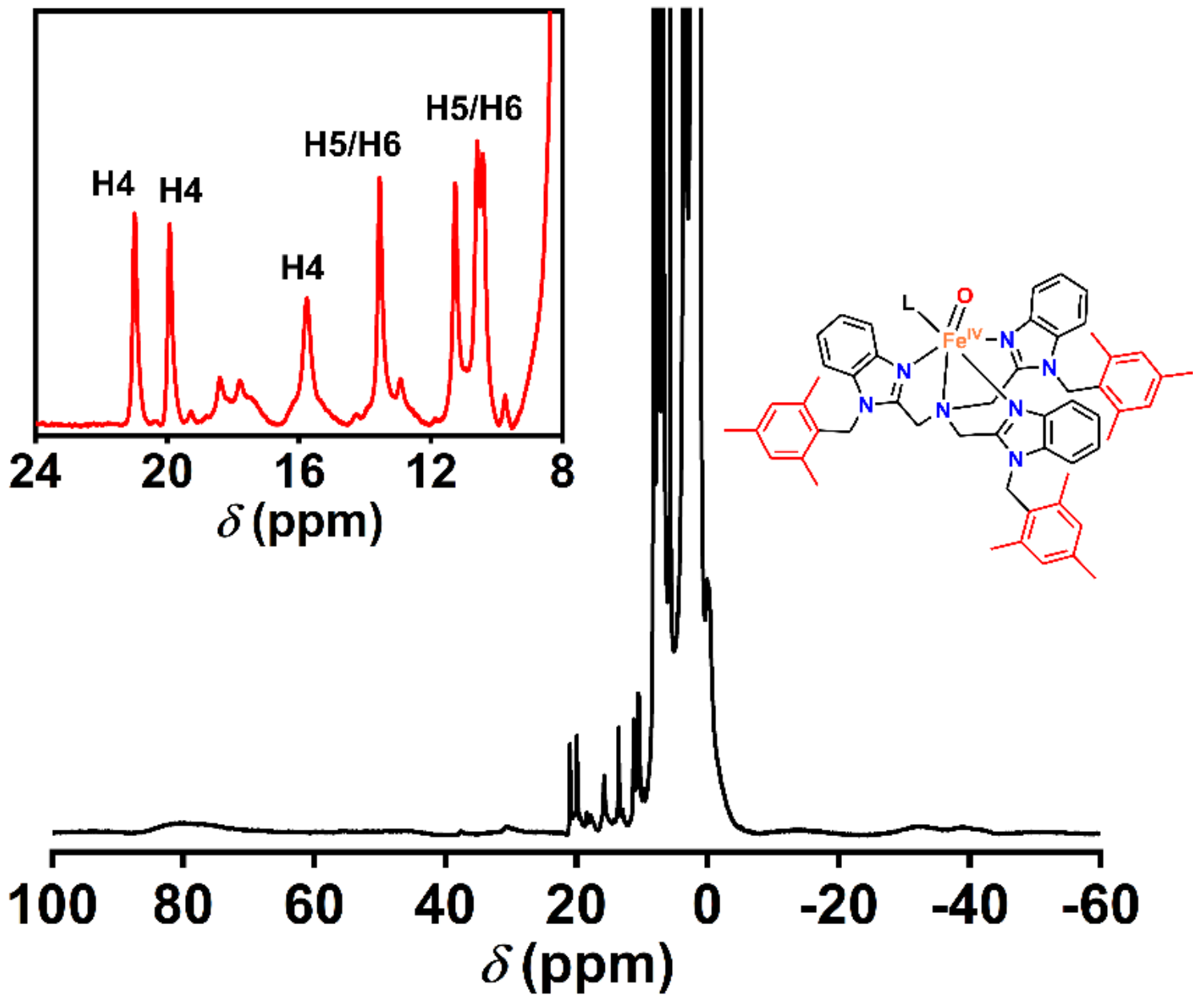

Figure S33 ^{1}H NMR spectra of complex **1O^{tmp}** [prepared by adding 2 equiv. of s-ArIO to a 2-mM solution made by dissolving solid **1^{tmp}** in acetone-d_6 : acetonitrile-d_3 (9:1)] at 193 K. Insets show the expanded region from 8 to 24 ppm.

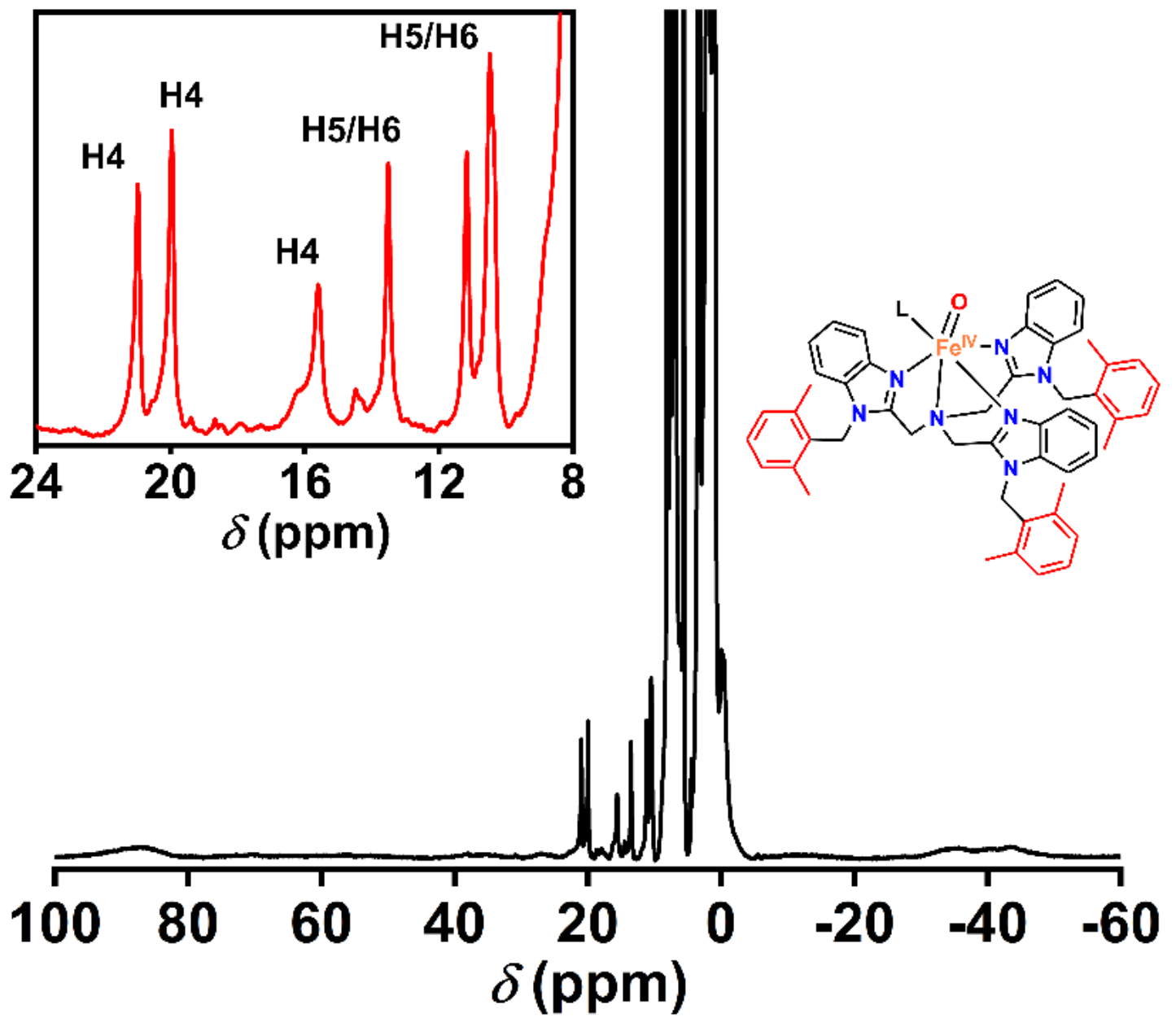

Figure S34 ^{1}H NMR spectra of complex **1O^{dmp}** [prepared by adding 2 equiv. of s-ArIO to a 2-mM solution made by dissolving solid **1^{dmp}** in acetone-d_6 : acetonitrile-d_3 (9:1)] at 193 K. Insets show the expanded region from 8 to 24 ppm.

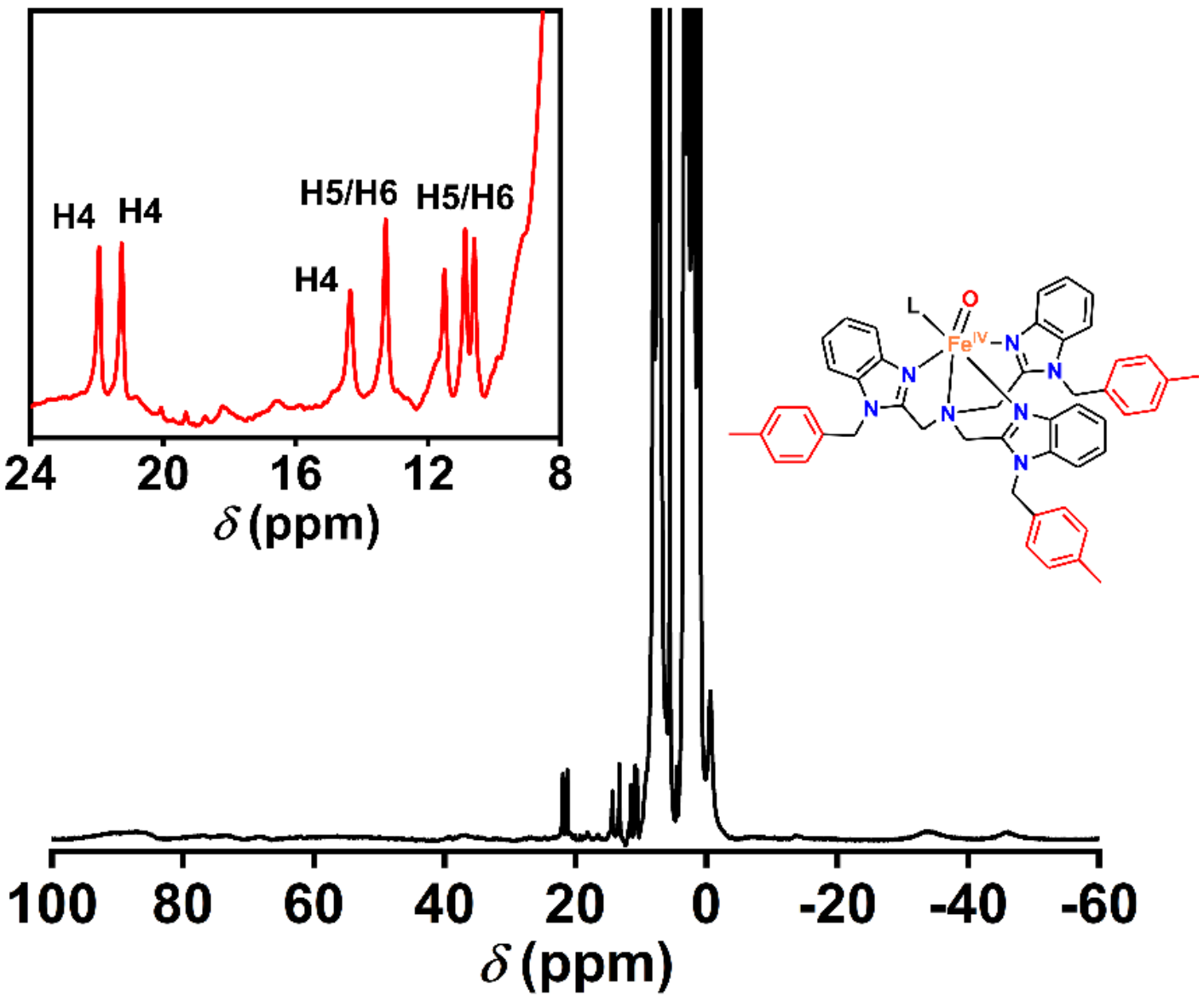

Figure S35 ^{1}H NMR spectra of complex **1O^mp** [prepared by adding 2 equiv. of s-ArIO to a 2-mM solution made by dissolving solid **1^mp** in acetone-d_6 : acetonitrile-d_3 (9:1)] at 193 K. Insets show the expanded region from 8 to 24 ppm.

Table S9. Table for ^{1}H NMR Signals of Benzimidazole Protons of [{(R-CH_2)$_3$NTB}Fe^{IV}(O)(L)]$^{2+}$ Complexes at 193 K (Where L represents a Solvent molecule)

Complex	H4 (ppm)	H5/H6 (ppm)
**1O^pfp**	21.3, 20.2, 15.8	13.6, 11.3, 11.1, 10.7
**1O^dfp**	20.9, 19.7, 15.5	13.6, 11.1, 10.9, 10.6
**1O^fp**	22.1, 21.4, 14.8	13.3, 11.5, 11.0, 10.6
**1O^mp**	22.0, 21.4, 14.5	13.4, 11.6, 11.0, 10.7
**1O^dmp**	21.0, 19.9, 15.6	13.5, 11.2, 10.5, 10.4
**1O^tmp**	21.0, 19.9, 15.8	13.6, 11.3, 10.6, 10.4
[a] **2O^*c*-propyl**	21.8, 21.1, 15.4	13.4, 11.1, 10.9, 10.6
[a] **2O^*c*-butyl**	21.7, 21.2, 15.9	13.3, 11.2, 11.0, 10.7
[a] **2O^*c*-pentyl**	21.8, 21.3, 15.8	13.3, 11.2, 11.1, 10.7
[a] **2O^*c*-hexyl**	22.3, 21.7, 14.8	13.3, 11.6, 11.1, 10.7
[a] **2O^adamantyl**	21.4, 20.5, 14.8	13.4, 12.0, 11.8, 10.7
[a] **2O^oxanyl**	22.2, 21.7, 15.4	13.3, 11.4, 11.2, 10.7
[a] **3O**	20.8, 20.0, 15.7	13.3, 11.0, 10.5, 10.1

[a] All the data have taken from reference [5].

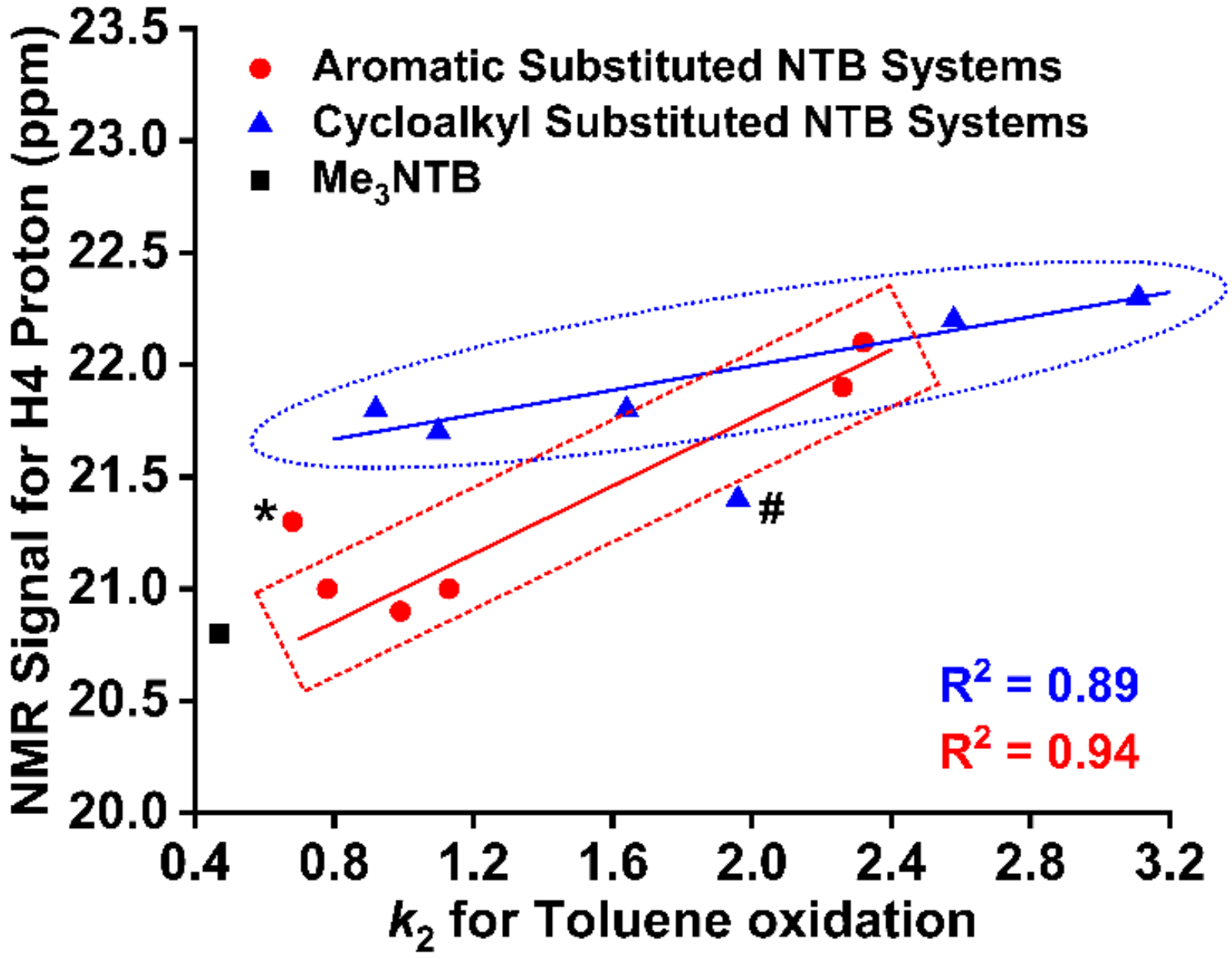

Figure S36 Correlation between the NMR signal of benzimidazole H4 proton with the toluene oxidation rates of various oxoiron(IV) complexes with NTB ligand backbones namely **1O**^{R}, **2O**^{R} and **3O**. Indicated are the outliers for each series: **1O**^{pfp} (*) and **2O**^{adamantyl} (#).

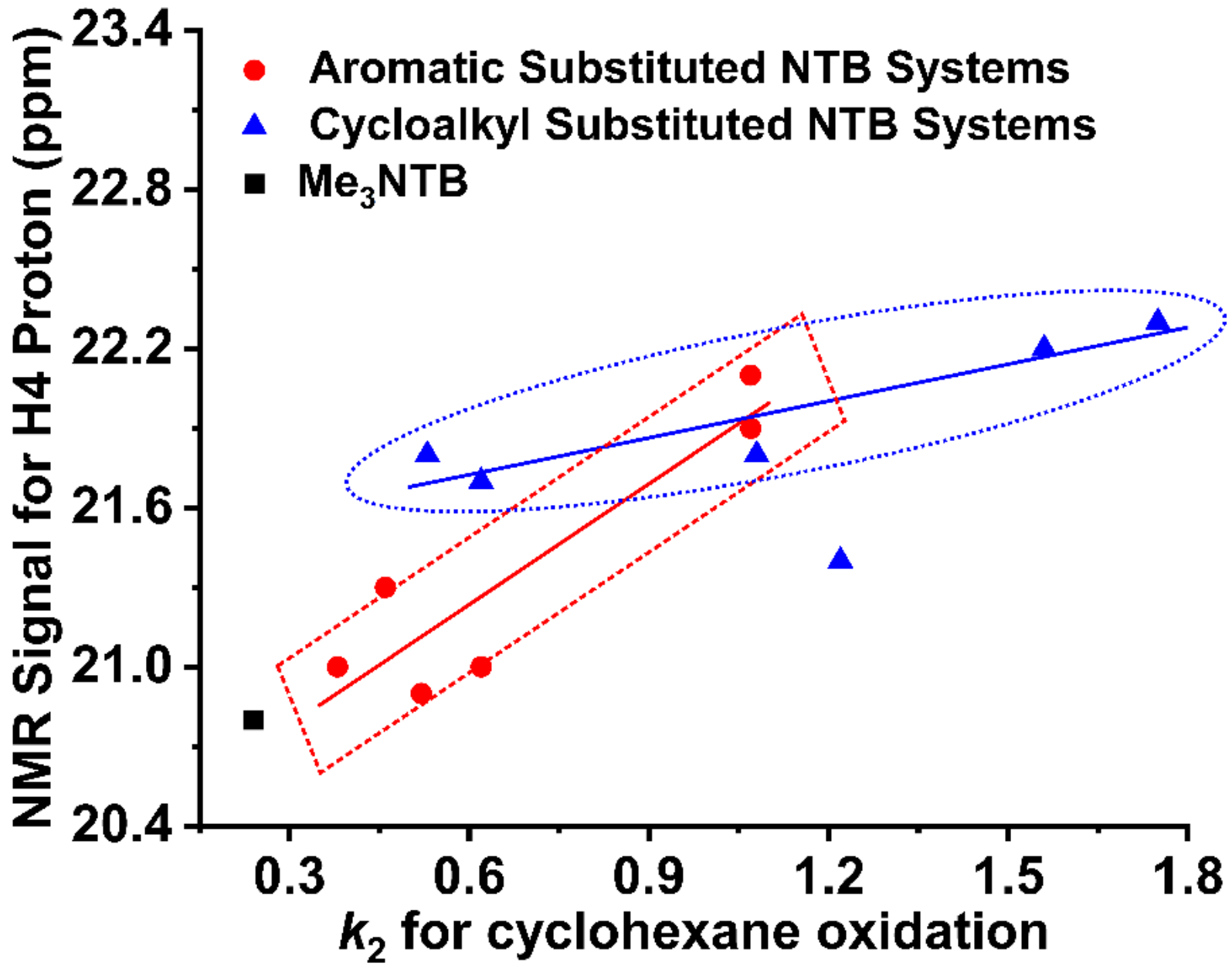

Figure S37 Correlation between the NMR signal of benzimidazole H4 proton with the cyclohexane oxidation rate of various iron(IV)-oxo complexes with NTB ligand backbone (**1O**^{R}, **2O**^{R} and **3O**).

F) Analysis of thermodynamic parameters using Eyring plots.

Temperature-dependent kinetic studies were performed to obtain the activation parameters for the reaction of iron-oxo intermediate with toluene. The decay of absorption band corresponding to the iron-oxo intermediate has been monitored. The Eyring plot has been plotted and it generates a straight line. From the slope and intercept of the straight line $\Delta H^{\ddagger}$ and $\Delta S^{\ddagger}$ parameters are calculated, respectively.

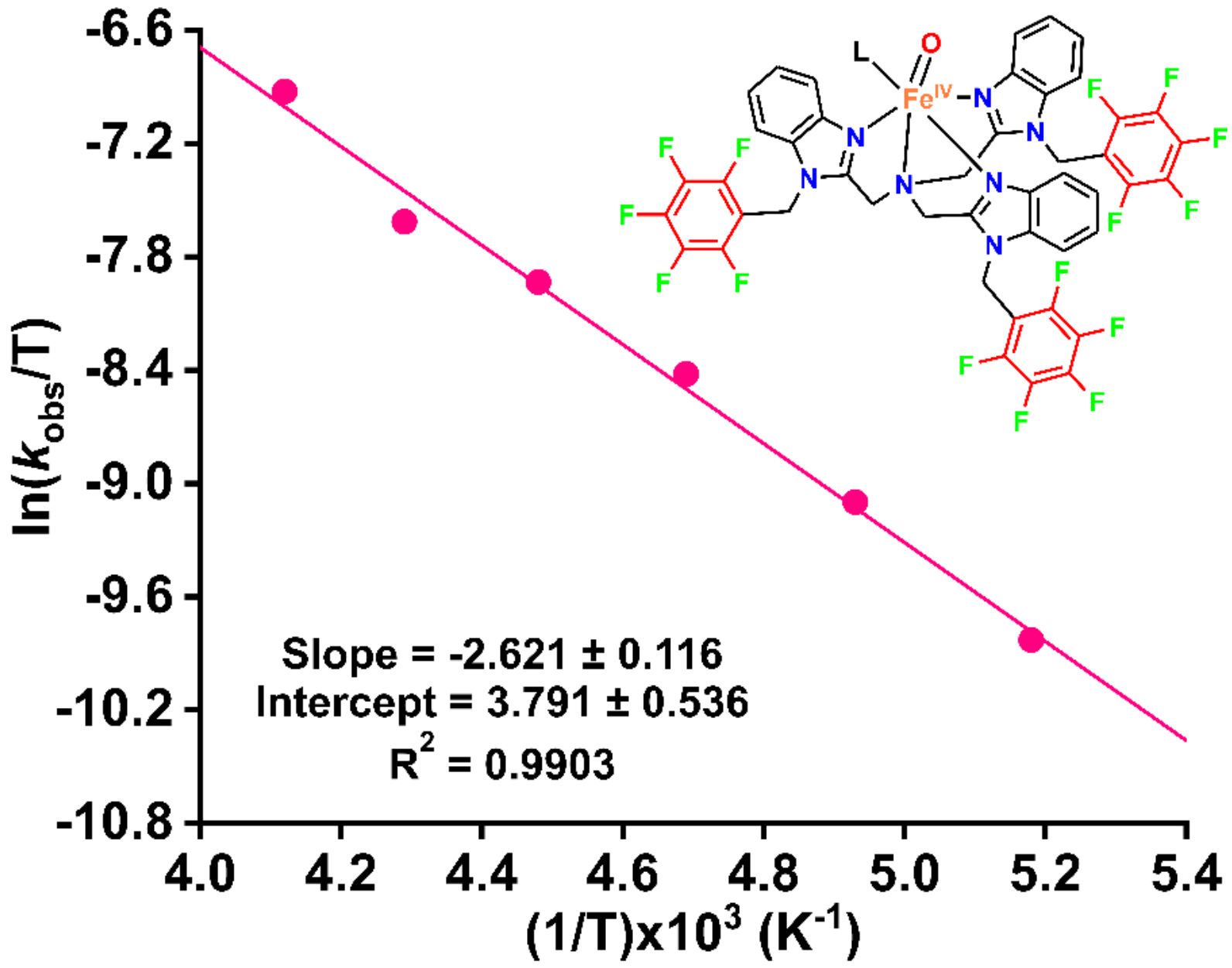

Figure S38 Eyring plot [ln(k/T) versus (1/T)] for the reaction of **1O^{pfp}** (1mM) with toluene (100 mM) in acetone-d_6 : acetonitrile-d_3 (9:1) [193 – 243 K] by following the decay of the 774 nm feature of **1O^{pfp}**.

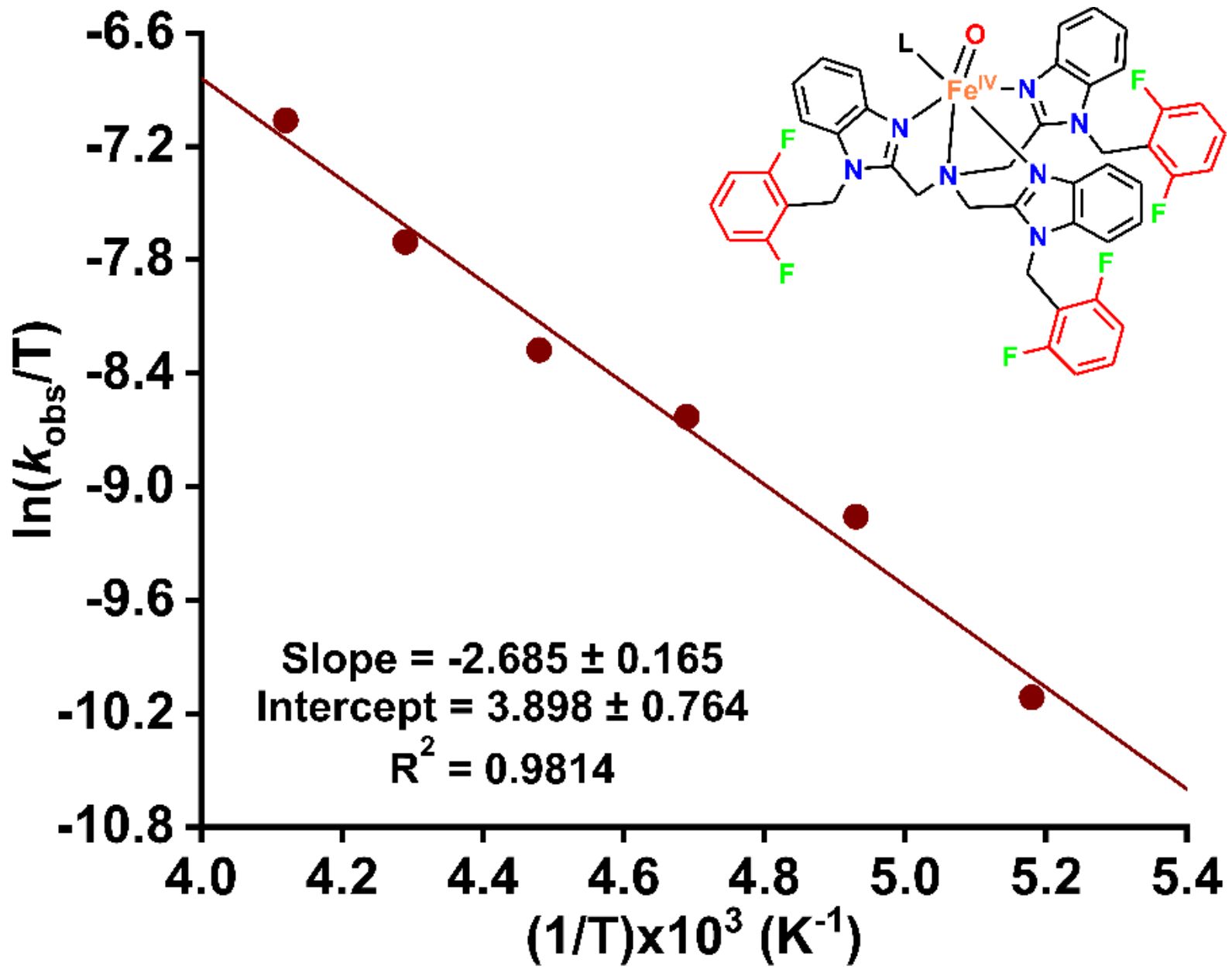

Figure S39 Eyring plot [ln(k/T) versus (1/T)] for the reaction of **1O^{dfp}** (1mM) with toluene (100 mM) in acetone-d_6 : acetonitrile-d_3 (9:1) [193 – 243 K] by following the decay of the 774 nm feature of **1O^{dfp}**.

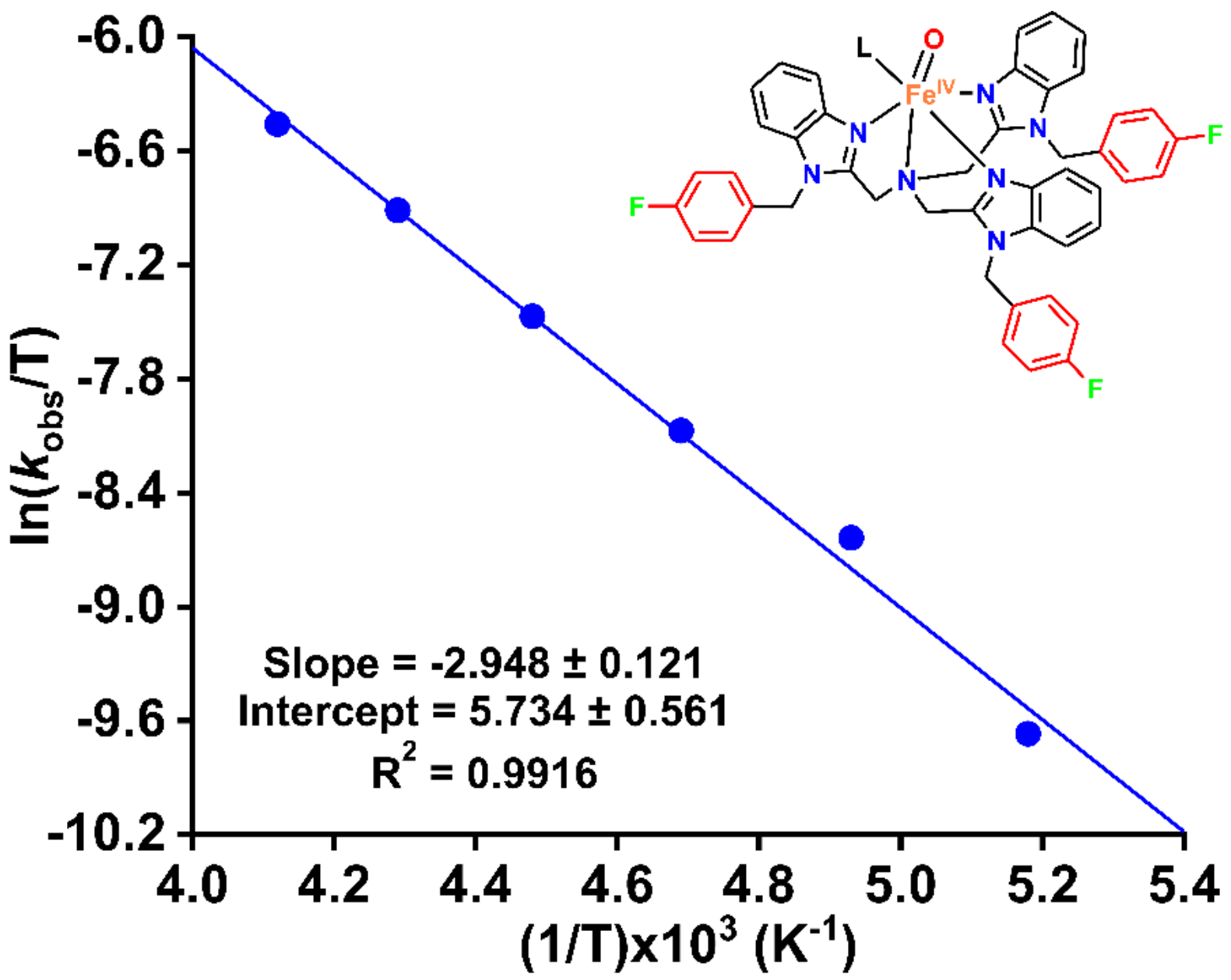

Figure S40 Eyring plot [ln(*k*/T) versus (1/T)] for the reaction of **1O^{fp}** (1mM) with toluene (100 mM) in acetone-d_6 : acetonitrile-d_3 (9:1) [193 – 243 K] by following the decay of the 771 nm feature of **1O^{fp}**.

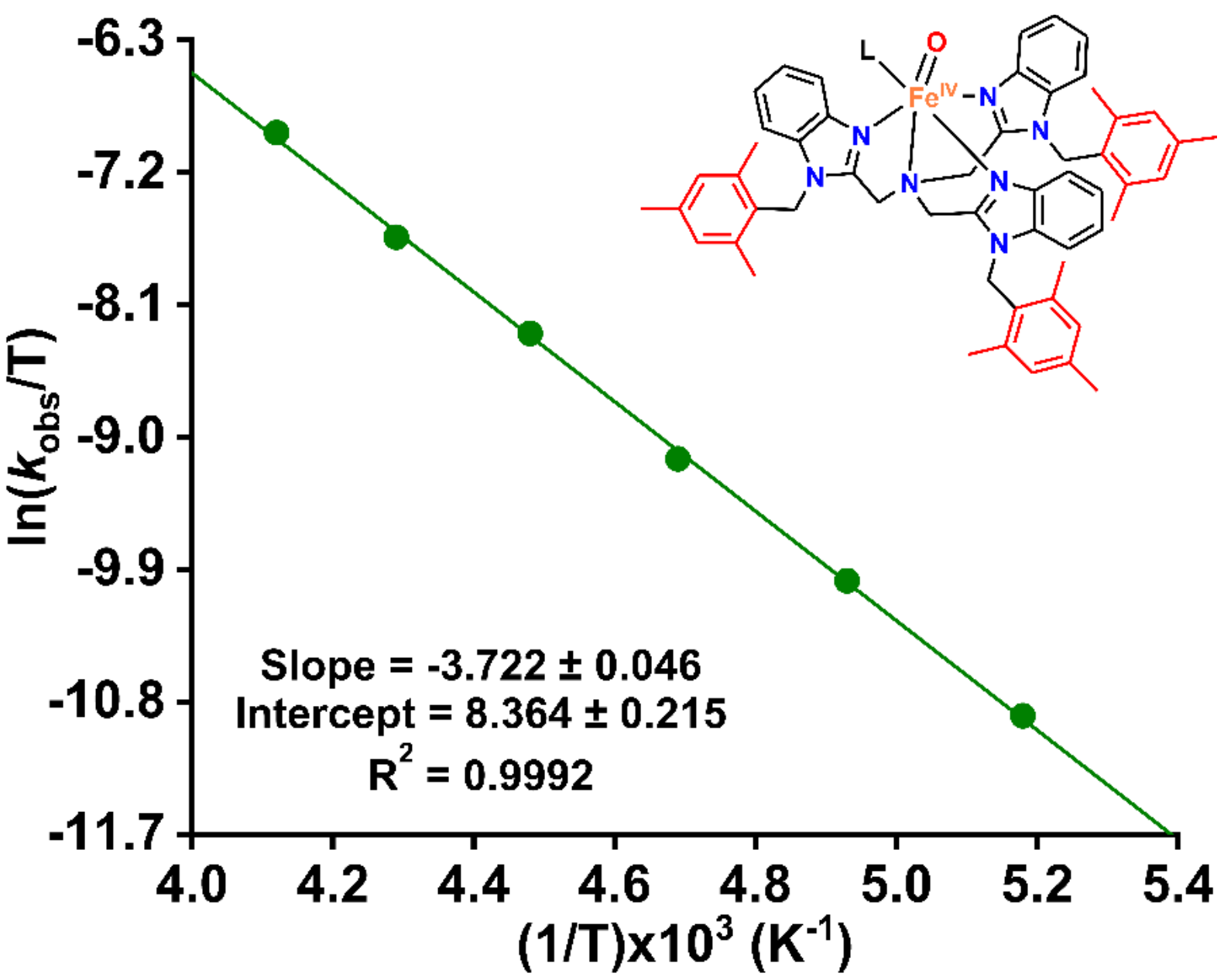

Figure S41 Eyring plot [ln(*k*/T) versus (1/T)] for the reaction of **1O^{tmp}** (1mM) with toluene (100 mM) in acetone-d_6 : acetonitrile-d_3 (9:1) [193 – 243 K] by following the decay of the 764 nm feature of **1O^{tmp}**.

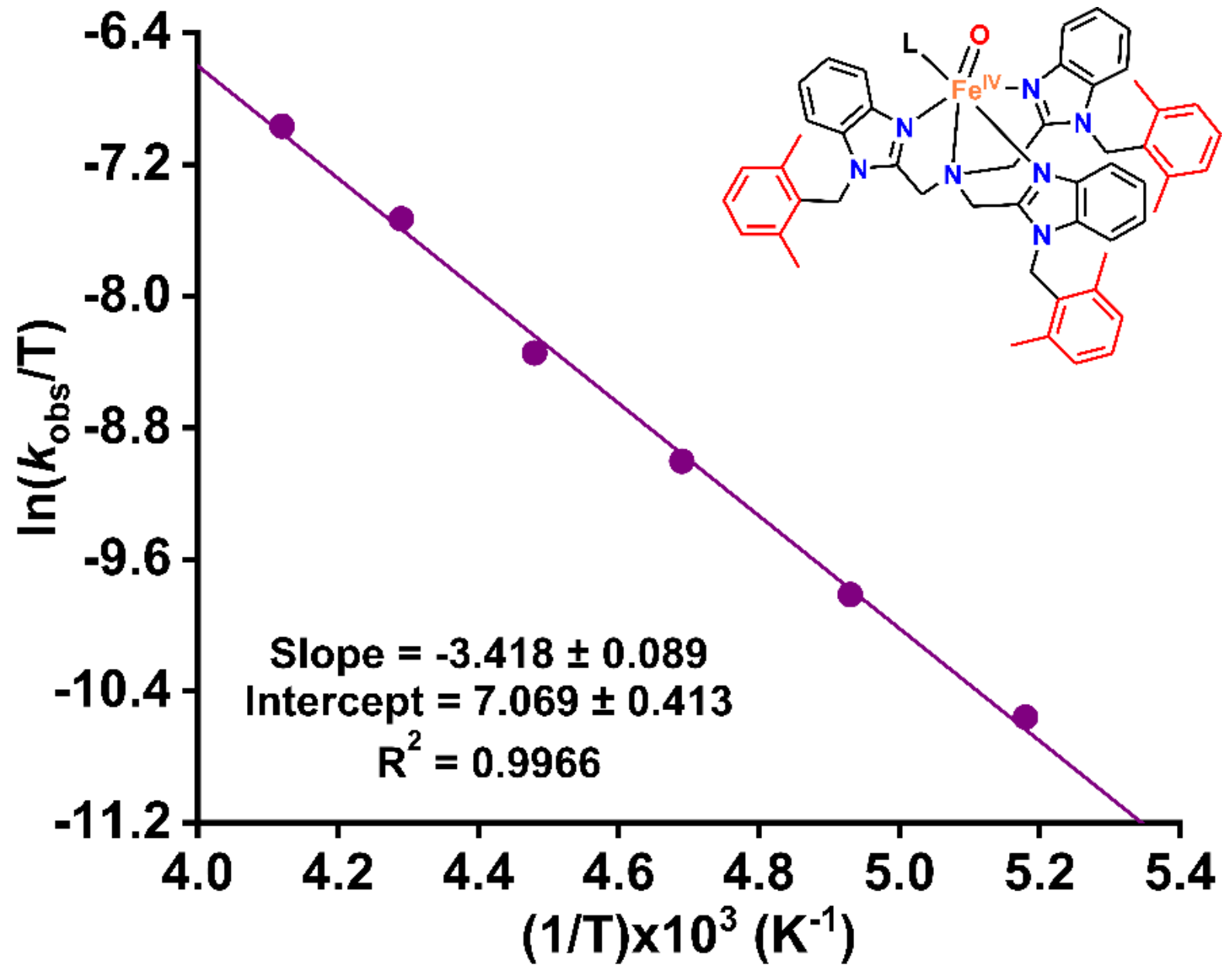

Figure S42 Eyring plot [ln(k/T) versus (1/T)] for the reaction of **1O**$^{\mathbf{dmp}}$ (1mM) with toluene (100 mM) in acetone-d_6 : acetonitrile-d_3 (9:1) [193 – 243 K] by following the decay of the 767 nm feature of **1O**$^{\mathbf{dmp}}$.

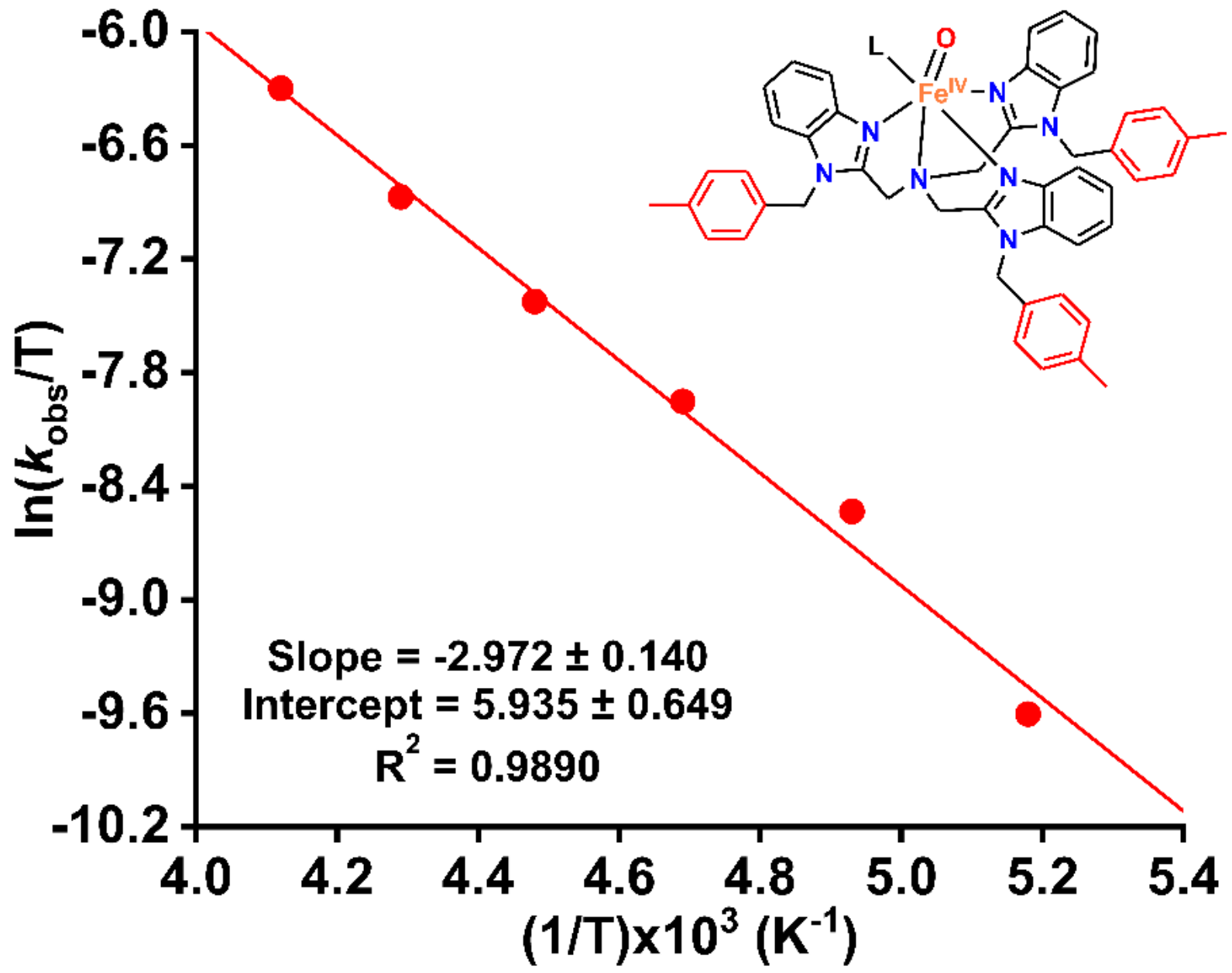

Figure S43 Eyring plot [ln(k/T) versus (1/T)] for the reaction of **1O**$^{\mathbf{mp}}$ (1mM) with toluene (100 mM) in acetone-d_6 : acetonitrile-d_3 (9:1) [193 – 243 K] by following the decay of the 772 nm feature of **1O**$^{\mathbf{mp}}$.

Table S10. Comparison of Thermodynamic Parameters for Reaction of $\mathbf{1O^R}$ Complexes with Toluene at 233 K. [Units of $\Delta H^{\ddagger}$ and $\Delta G^{\ddagger}$: $kJmol^{-1}$; Units of $\Delta S^{\ddagger}$: $Jmol^{-1}$ K^{-1}].

	Experimental			From k_2[a]
Complex	$\Delta H^{\ddagger}$	$\Delta S^{\ddagger}$	$\Delta G^{\ddagger}$ at 233 K	$\Delta G^{\ddagger}$ at 233 K
$1O^{pfp}$	21.79 ± 0.97	-166.02 ± 4.45	60.47	61.46
$1O^{dfp}$	22.32 ± 1.37	-165.13 ± 6.35	60.79	60.73
$1O^{fp}$	24.51 ± 1.01	-149.87 ± 4.67	59.43	59.08
$1O^{mp}$	24.71 ± 0.39	-148.20 ± 5.40	59.24	59.13
$1O^{dmp}$	28.42 ± 0.74	-138.77 ± 3.43	60.75	60.47
$1O^{tmp}$	30.94 ± 1.16	-128.00 ± 1.79	60.76	61.19
[b] $2O^{c\text{-propyl}}$	21.90 ± 0.82	-164.48 ± 3.84	60.23	60.92
[b] $2O^{c\text{-butyl}}$	21.43 ± 0.87	-165.55 ± 4.03	59.99	60.57
[b] $2O^{c\text{-pentyl}}$	21.63 ± 0.80	-164.07 ± 3.69	59.86	59.79
[b] $2O^{adamantyl}$	24.08 ± 0.39	-153.04 ± 1.78	59.74	59.45
[b] $2O^{oxanyl}$	23.70 ± 0.49	-152.92 ± 2.22	59.33	58.92
[b] $2O^{c\text{-hexyl}}$	22.99 ± 0.44	-155.80 ± 2.02	59.29	58.56
3O	15.12 ± 1.14	-195.69 ± 5.31	60.71	62.22

[a] Calculated using the Eyring-Polanyi equation[20]; transmission coefficient κ: 8.4. [b] Data obtained from reference [5].

5. Figures Related to Discussion Section

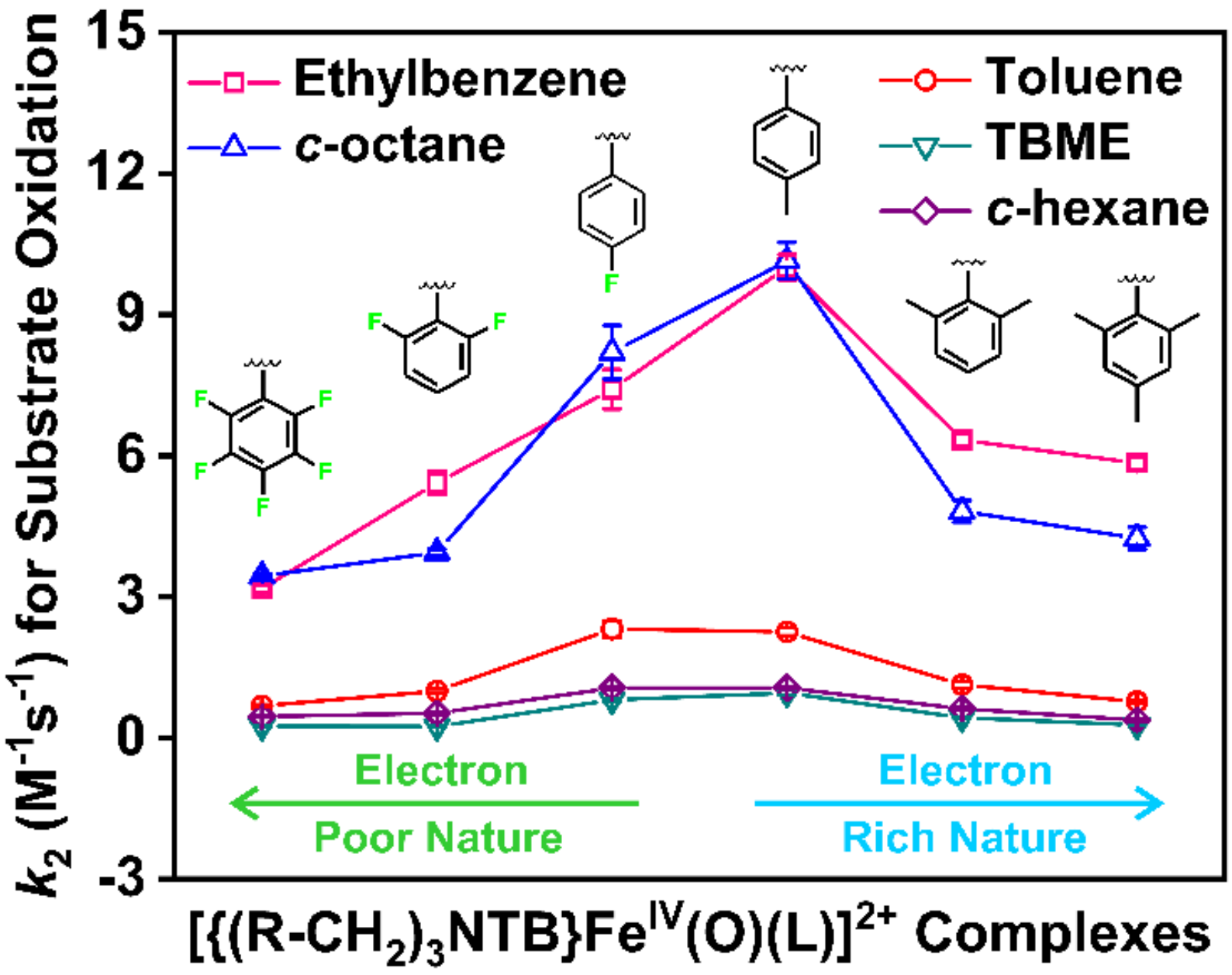

Figure S44 Graphical representation of the substrate oxidation rates for the series of $[\{R\text{-}CH_2)_3NTB\}Fe^{IV}(O)(L)]^{2+}$ complexes, where R refers to different aromatic substituents. Error bars represent ± SD (n=3).

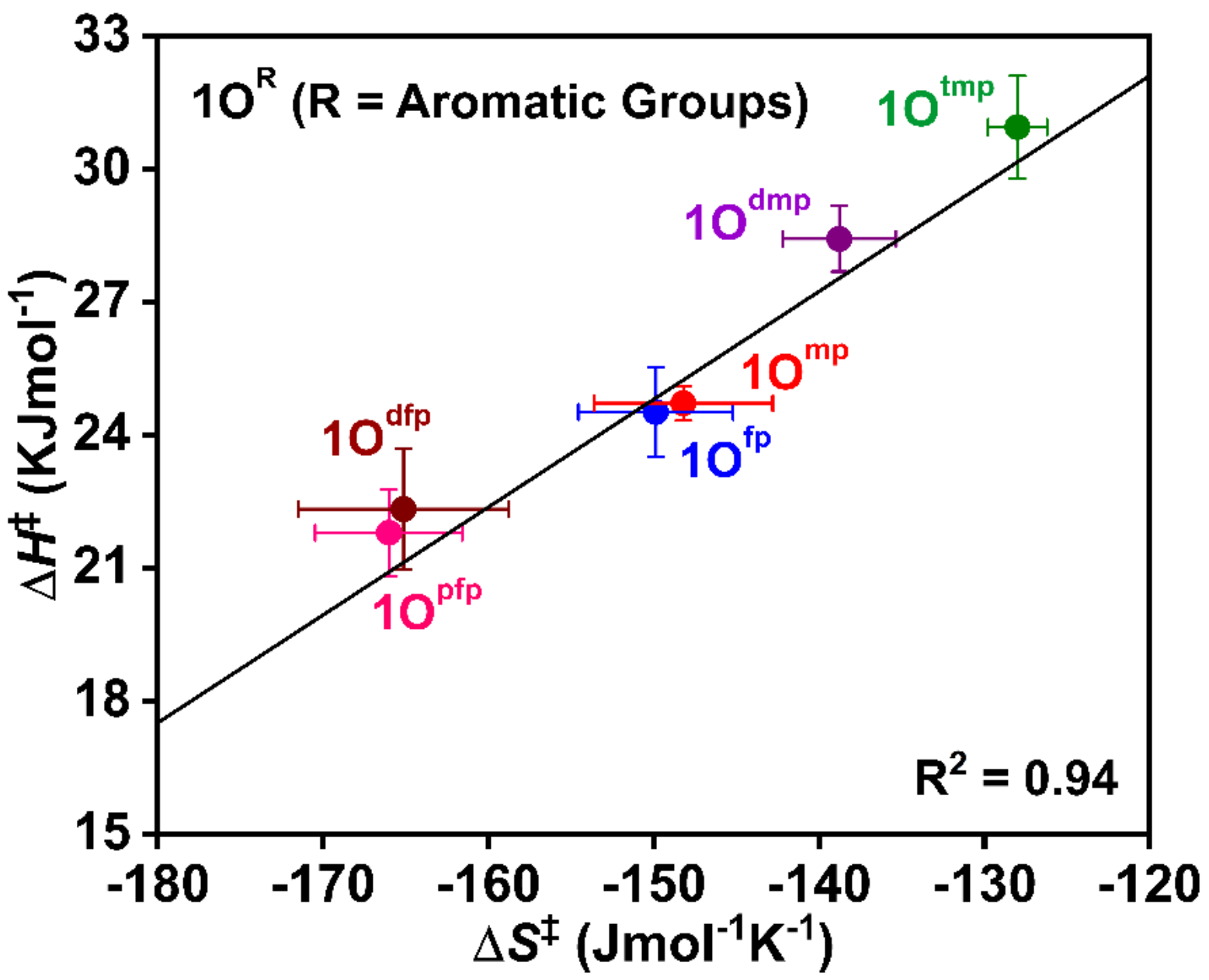

Figure S45 Plot of $\Delta H^{\ddagger}$ against $\Delta S^{\ddagger}$ in the C-H activation of toluene by the **1O^R** series of complexes. Error bars represent ± SD (n=3).

6. Computational Details

All DFT calculations were performed with the Amsterdam Density Functional (ADF),[21-22] and QUILD[23] programs. Molecular orbitals were expanded in an uncontracted set of Slater-type orbitals (STOs) of triple-ζ quality with double polarization functions (TZ2P).[24-25] Core electrons were not treated explicitly during the geometry optimizations (frozen core approximation[21]). An auxiliary set of s, p, d, f, and g STOs was used to fit the molecular density and to represent the Coulomb and exchange potentials accurately for each SCF cycle.

Geometries were optimized with the QUILD[23] program using adapted delocalized coordinates until the maximum gradient component was less than 10^{-4} a.u. Energies, gradients, Hessians[26] (for vibrational frequencies) were calculated using S12g,[27] in all cases by including solvation effects through the COSMO[28] dielectric continuum model with appropriate parameters for the solvent (acetone).[29] For computing Gibbs free energies, all small frequencies were raised to 100 cm^{-1} in order to compensate for the breakdown of the harmonic oscillator model.[30-31] Scalar relativistic corrections have been included self-consistently in all calculations by using the zeroth-order regular approximation (ZORA).[32] Most S12g calculations were performed with a Becke grid[33-34] of VeryGood quality, except the frequencies which were computed with a Normal grid. All DFT calculations were performed using the unrestricted Kohn-Sham scheme.

All computational data will be uploaded onto the IOCHEM-BD platform (www.iochem-bd.org), to facilitate data exchange and dissemination, according to the FAIR principles[35] of OpenData sharing.

Table S11. Experimental and computed Mössbauer parameters ($mm \cdot s^{-1}$) for **1O^R** Fe(IV)=O complexes, computed at S12g/TZ2P (COSMO, ZORA)

Complex	IS^{exp}	$\|QS^{exp}\|$	IS^{par} [a]	QS^{par} [a]	IS^{perp} [b]	QS^{perp} [b]
**1O^pfp**	0.05	1.52	-0.016	1.213	0.047	-1.714
**1O^dfp**	0.05	1.53	0.073	1.626	-0.026	1.270
**1O^fp**	0.04	1.52	0.067	1.569	-0.028	1.303
**1O^mp**	0.05	1.55	0.059	1.674	-0.026	1.307
**1O^dmp**	0.05	1.53	0.053	-1.719	-0.029	1.296
**1O^tmp**	0.05	1.53	0.055	-1.712	-0.030	1.305

[a] with aromatic rings in parallel orientation; [b] with aromatic rings in perpendicular orientation.

Parallel (left) vs. perpendicular (right) orientations of the aromatic rings w.r.t. Fe=O axis

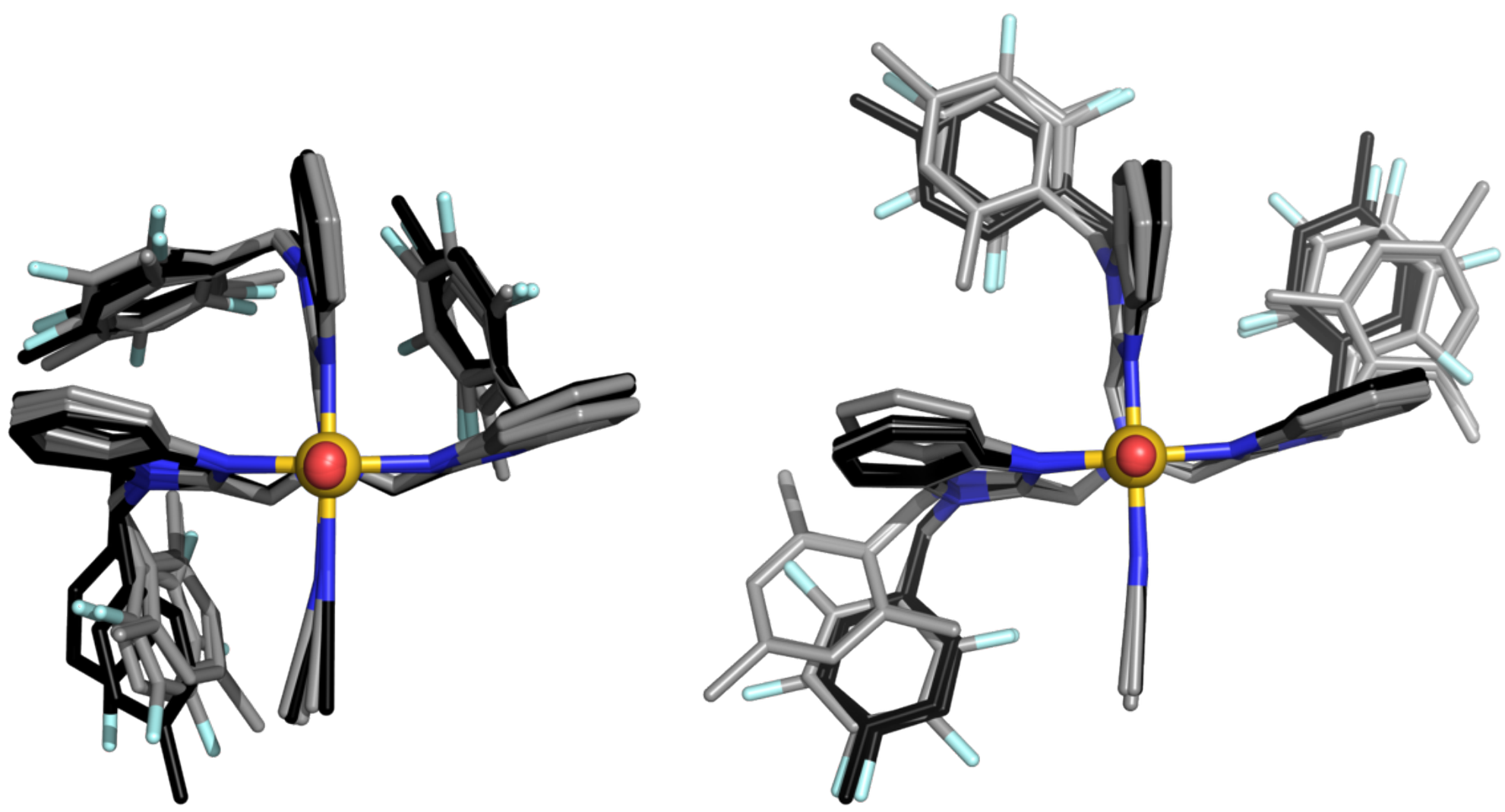

Table S12. Comparison of thermodynamics ($\Delta H^‡$ and $\Delta G^‡$ in kJ· mol^{-1}, $\Delta S^‡$ in J·mol^{-1} K^{-1}) for reaction with cyclohexane at 233 K, computed at S12g/TZ2P (COSMO, ZORA)

parallel	w.r.t. Fe^{IV}=O			w.r.t. RC		
Complex	$\Delta H^‡$	$\Delta S^‡$	$\Delta G^‡$	$\Delta H^‡$	$\Delta S^‡$	$\Delta G^‡$
1O^{dfp}	*17.95*	*-187.19*	*36.56*	*74.35*	*23.26*	*44.84*
**1O^{fp} [a]**	21.34	-186.59	41.54	67.38	22.32	38.56
**1O^{mp} [a]**	18.97	-187.73	37.09	68.63	19.82	40.23
**1O^{dmp} [a]**	34.13	-176.79	55.02	68.58	15.66	41.61
perpend.	w.r.t. Fe^{IV}=O			w.r.t. RC		
Complex	$\Delta H^‡$	$\Delta S^‡$	$\Delta G^‡$	$\Delta H^‡$	$\Delta S^‡$	$\Delta G^‡$
**1O^{dfp} [a]**	34.62	-171.20	54.42	78.01	22.82	48.94
1O^{fp}	*8.71*	*-181.42*	*31.86*	*68.09*	*21.46*	*39.74*
1O^{mp}	*1.36*	*-171.77*	*22.45*	*70.22*	*21.78*	*41.62*
1O^{dmp}	*38.57*	*-174.85*	*59.16*	*77.43*	*21.01*	*48.72*
cycloalkyls	w.r.t. Fe^{IV}=O			w.r.t. RC		
Complex	$\Delta H^‡$	$\Delta S^‡$	$\Delta G^‡$	$\Delta H^‡$	$\Delta S^‡$	$\Delta G^‡$
**2O$^{c\text{-propyl}}$ [b]**	15.10	-190.41	59.46	48.36	10.99	45.80
**2O$^{c\text{-hexyl}}$ [b]**	10.52	-173.16	50.87	45.78	31.59	38.42
**3O [b]**	23.07	-183.65	65.86	49.84	8.97	47.75
**4O [b]**	13.10	-190.11	57.40	45.87	3.30	45.10

[a] Lowest energy confomer according to DFT; [b] Data from reference [5].

Note that according to our DFT calculations, the **1O^{dfp}** and **1O^{pfp}** complexes are most stable in the perpendicular orientation. For the **1O^{pfp}** complex this is consistent with the computed Mössbauer parameter.

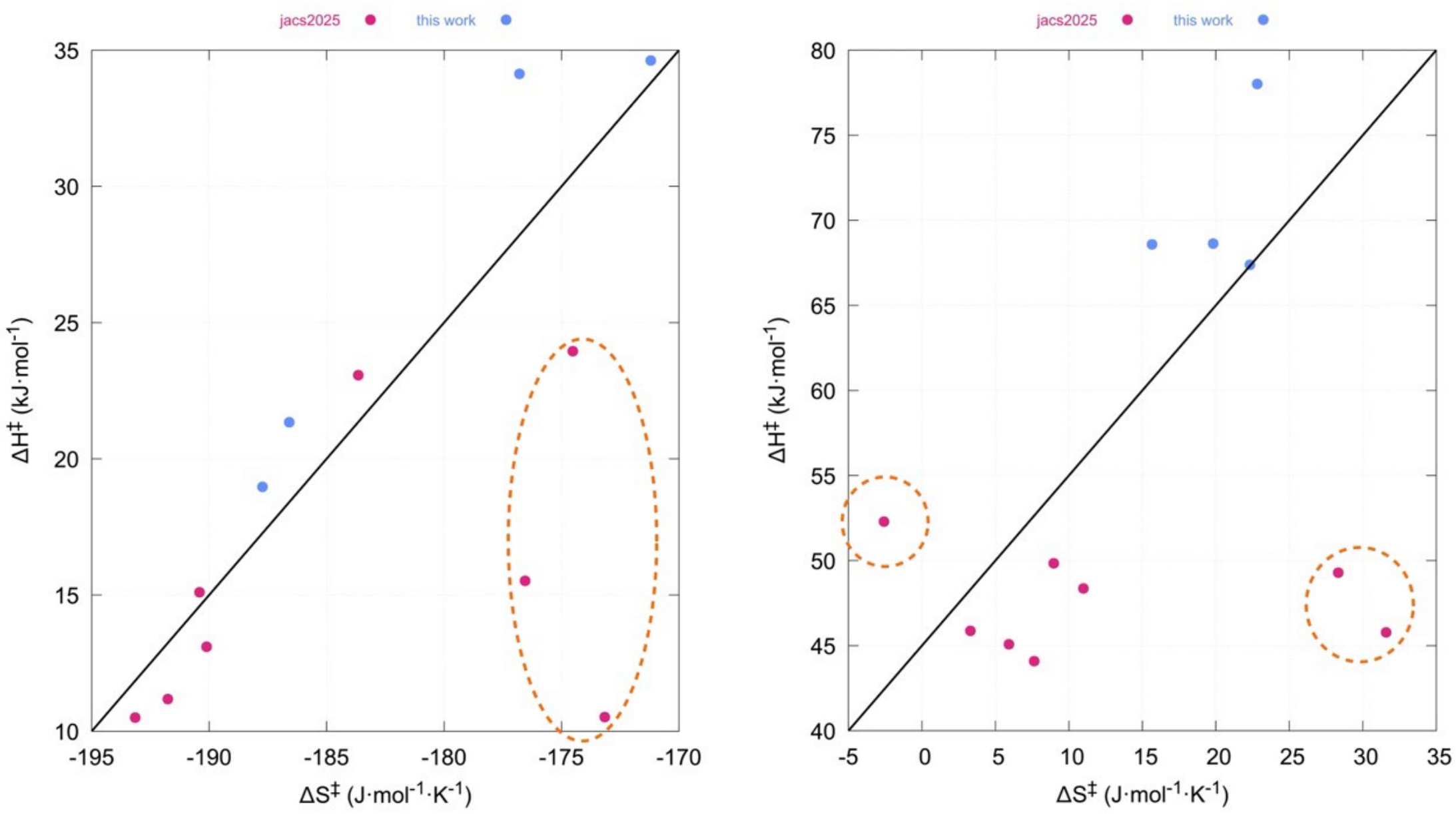

Figure S46. Plot of $\Delta H^{\ddagger}$ against $\Delta S^{\ddagger}$ in the C-H activation of cyclohexane, as computed by S12g/TZ2P (COSMO, ZORA) for the **2O$^{\mathbf{c\text{-}hexyl}}$** complexes (data from ref. [5]) and the four most reactive **1O$^{\mathbf{R}}$** complexes. Shown on the left are the data w.r.t. Fe^{IV}=O (R^2=0.91 for the diagonal line, if the three outliers from ref. [5] are not taken into account), on the right w.r.t. RC (R^2=0.73 for diagonal line, without the outliers). Note that the three outliers are the three largest cycloalkyl substituents (cyclohexyl, oxanyl, adamantyl). The reason for them being outliers in calculations, but not in experiments, is most likely to their bulky character and conformational flexibility. Because of the system sizes of these structures, a detailed study on the effect of this conformational flexibility on the barriers is beyond the available computational resources at our lab.

References

[1] Petasis, D. T., Hendrich, M. P. 2015, "Quantitative Interpretation of Multifrequency Multimode EPR Spectra of Metal Containing Proteins, Enzymes, and Biomimetic Complexes." in *Methods in Enzymology*. edited by Qin, P. Z., Warncke, K.: Academic Press. ISBN 0076-6879.

[2] Chen, B.-T., Morlanés, N., Adogla, E., Takanabe, K., Rodionov, V. O., "An Efficient and Stable Hydrophobic Molecular Cobalt Catalyst for Water Electro-oxidation at Neutral pH," *ACS Catal.* 6, no. 7 (2016), https://doi.org/10.1021/acscatal.6b01237.

[3] Biswas, A. N., Puri, M., Meier, K. K., et al., "Modeling TauD-J: A High-Spin Nonheme Oxoiron(IV) Complex with High Reactivity toward C–H Bonds," *J. Am. Chem. Soc.* 137, no. 7 (2015), https://doi.org/10.1021/ja511757j.

[4] Macikenas, D., Skrzypczak-Jankun, E., Protasiewicz, J. D., "A New Class of Iodonium Ylides Engineered as Soluble Primary Oxo and Nitrene Sources," *J. Am. Chem. Soc.* 133, no. 11 (2011), https://doi.org/10.1021/ja200554v.

[5] Das, A., Pal, N., Xiong, J., et al., "10-Fold Increase in Hydrogen Atom Transfer Reactivity for a Series of S = 1 FeIV═O Complexes Over the S = 2 [(TQA)FeIV═O]2+ Complex via Entropic Lowering of Reaction Barriers by Secondary Sphere Cycloalkyl Substitution," *J. Am. Chem. Soc.* 147, no. 1 (2025), https://doi.org/10.1021/jacs.4c10120.

[6] Banerjee, S., Rasheed, W., Fan, R., et al., "NMR Reveals That a Highly Reactive Nonheme FeIV=O Complex Retains Its Six-Coordinate Geometry and S=1 State in Solution," *Chem. Eur. J.* 25, no. 41 (2019), https://doi.org/https://doi.org/10.1002/chem.201902048.

[7] APEX5 2014, "APEX5, Bruker Analytical X-ray Systems, Madison, WI (2014)." Madison, WI: Bruker Analytical X-ray Systems.

[8] SADABS 2014, "SADABS, Bruker Analytical X-ray Systems, Madison, WI (2014)." Madison, WI: Bruker Analytical X-ray Systems.

[9] SAINT 2014, "SAINT Bruker Analytical X-ray Systems, Madison, WI (2014)." Madison, WI: Bruker Analytical X-ray Systems.

[10] Sheldrick, G., "A short history of SHELX," *Acta Cryst. A.* 64, no. 1 (2008), https://doi.org/doi:10.1107/S0108767307043930.

[11] SHELXTL 2013, "SHELXTL 2018, Bruker Analytical X-Ray Systems, Madison, WI (2013)." 2018 Ed. Madison, WI: Bruker Analytical X-Ray Systems.

[12] Seo, M. S., Kim, N. H., Cho, K.-B., et al., "A mononuclear nonheme iron(iv)-oxo complex which is more reactive than cytochrome P450 model compound I," *Chem. Sci.* 2, no. 6 (2011), https://doi.org/10.1039/C1SC00062D.

[13] Pal, N., Xiong, J., Jahja, M., et al., "A 5,000-fold increase in the HAT reactivity of a nonheme Fe(IV)=O complex simply by replacing two pyridines of the pentadentate N4Py ligand with pyrazoles," *Proc. Natl. Acad. Sci. U. S. A.* 122, no. 6 (2025), https://doi.org/doi:10.1073/pnas.2414962122.

[14] Rasheed, W., Pal, N., Aboelenen, A. M., et al., "NMR and Mössbauer Studies Reveal a Temperature-Dependent Switch from S = 1 to 2 in a Nonheme Oxoiron(IV) Complex with Faster C–H Bond Cleavage Rates," *J. Am. Chem. Soc.* 146, no. 6 (2024), https://doi.org/10.1021/jacs.3c10694.

[15] Monte Pérez, I., Engelmann, X., Lee, Y.-M., et al., "A Highly Reactive Oxoiron(IV) Complex Supported by a Bioinspired N3O Macrocyclic Ligand," *Angew. Chem. Int. Ed.* 56, no. 46 (2017), https://doi.org/https://doi.org/10.1002/anie.201707872.

[16] Warm, K., Paskin, A., Kuhlmann, U., et al., "A Pseudotetrahedral Terminal Oxoiron(IV) Complex: Mechanistic Promiscuity in C−H Bond Oxidation Reactions," *Angew. Chem. Int. Ed.* 60, no. 12 (2021), https://doi.org/https://doi.org/10.1002/anie.202015896.

[17] Comba, P., Fukuzumi, S., Koke, C., Martin, B., Löhr, A.-M., Straub, J., "A Bispidine Iron(IV)–Oxo Complex in the Entatic State," *Angew. Chem. Int. Ed.* 55, no. 37 (2016), https://doi.org/https://doi.org/10.1002/anie.201605099.
[18] Comba, P., Nunn, G., Scherz, F., Walton, P. H., "Intermediate-spin iron(iv)-oxido species with record reactivity," *Faraday Discuss.* 234, no. 0 (2022), https://doi.org/10.1039/D1FD00073J.
[19] Abu-Odeh, M., Bleher, K., Johnee Britto, N., et al., "Pathways of the Extremely Reactive Iron(IV)-oxido complexes with Tetradentate Bispidine Ligands," *Chem. Eur. J.* 27, no. 44 (2021), https://doi.org/https://doi.org/10.1002/chem.202101045.
[20] Cho, K.-B., Kim, E. J., Seo, M. S., Shaik, S., Nam, W., "Correlating DFT-Calculated Energy Barriers to Experiments in Nonheme Octahedral FeIVO Species," *Chem. Eur. J.* 18, no. 33 (2012), https://doi.org/https://doi.org/10.1002/chem.201200096.
[21] te Velde, G., Bickelhaupt, F. M., Baerends, E. J., et al., "Chemistry with ADF," *J. Comput. Chem.* 22, no. 9 (2001), https://doi.org/https://doi.org/10.1002/jcc.1056.
[22] Baerends, E. J., Ziegler, T., Atkins, A. J., et al. 2019, "ADF 2019." 2019.3 Ed. SCM, Theoretical Chemistry, Vrije Universiteit, Amsterdam, The Netherlands.
[23] Swart, M., Bickelhaupt, F. M., "QUILD: QUantum-regions interconnected by local descriptions," *J. Comput. Chem.* 29, no. 5 (2008), https://doi.org/https://doi.org/10.1002/jcc.20834.
[24] van Lenthe, E., Baerends, E. J., "Optimized Slater-type basis sets for the elements 1–118," *J. Comput. Chem.* 24, no. 9 (2003), https://doi.org/https://doi.org/10.1002/jcc.10255.
[25] Chong, D. P., van Lenthe, E., van Gisbergen, S. J. A., Baerends, E. J., "Even-tempered slater-type orbitals revisited: From hydrogen to krypton," *J. Comput. Chem.* 25, no. 8 (2004), https://doi.org/https://doi.org/10.1002/jcc.20030.
[26] Wolff, S. K., "Analytical second derivatives in the Amsterdam density functional package," *Int. J. Quantum Chem.* 104, no. 5 (2005), https://doi.org/https://doi.org/10.1002/qua.20653.
[27] Swart, M., "A new family of hybrid density functionals," *Chem. Phys. Lett.* 580(2013), https://doi.org/https://doi.org/10.1016/j.cplett.2013.06.045.
[28] Klamt, A., Schüürmann, G., "COSMO: a new approach to dielectric screening in solvents with explicit expressions for the screening energy and its gradient," *J. Chem. Soc., Perkin Trans. 2*, no. 5 (1993), https://doi.org/10.1039/P29930000799.
[29] Swart, M., Rösler, E., Bickelhaupt, F. M., "Proton Affinities in Water of Maingroup-Element Hydrides – Effects of Hydration and Methyl Substitution," *Eur. J. Inorg. Chem.* 2007, no. 23 (2007), https://doi.org/https://doi.org/10.1002/ejic.200700228.
[30] Averkiev, B. B., Truhlar, D. G., "Free energy of reaction by density functional theory: oxidative addition of ammonia by an iridium complex with PCP pincer ligands," *Catal. Sci. Technol.* 1, no. 8 (2011), https://doi.org/10.1039/C1CY00227A.
[31] Klein, J. E. M. N., Dereli, B., Que, L., Jr., Cramer, C. J., "Why metal–oxos react with dihydroanthracene and cyclohexadiene at comparable rates, despite having different C–H bond strengths. A computational study," *Chem. Commun.* 52, no. 69 (2016), https://doi.org/10.1039/C6CC05395E.
[32] van Lenthe, E., Baerends, E. J., Snijders, J. G., "Relativistic regular two-component Hamiltonians," *J. Chem. Phys.* 99, no. 6 (1993), https://doi.org/10.1063/1.466059 %J The Journal of Chemical Physics.
[33] Becke, A. D., "A multicenter numerical integration scheme for polyatomic molecules," *J. Chem. Phys.* 88, no. 4 (1988), https://doi.org/10.1063/1.454033 %J The Journal of Chemical Physics.

[34] Franchini, M., Philipsen, P. H. T., Visscher, L., "The Becke Fuzzy Cells Integration Scheme in the Amsterdam Density Functional Program Suite," *J. Comput. Chem.* 34, no. 21 (2013), https://doi.org/https://doi.org/10.1002/jcc.23323.
[35] Wilkinson, M. D., Dumontier, M., Aalbersberg, I. J., et al., "The FAIR Guiding Principles for scientific data management and stewardship," *Scientific Data* 3, no. 1 (2016), https://doi.org/10.1038/sdata.2016.18.
